# The Secret Life of Tidal Marshes and Mangroves: Camera Trapping as a Window Into Wildlife Using North American Coastal Wetlands

**DOI:** 10.1002/ece3.72872

**Published:** 2026-01-15

**Authors:** Kenneth B. Raposa, Kimberly Cressman, Danika vanProosdij, Jason Goldstein, Rachel A. Stevens, Megan Tyrrell, Brian DeGasperis, Kari St. Laurent, R. Kyle Derby, Scott Lerberg, Elizabeth Fox Pinnix, Jennifer Plunkett, Jessica Kinsella, Colby Peffer, Candace Killian, Jay Black, Katie Swanson, Christopher Biggs, Emily Kuzmick, Angel Dieppa‐Ayala, Kristin Wilson Grimes, Allie Durdall, Jacob Argueta, Thomas Reid, Roger Fuller, Jennifer Schmitt, Matthew C. Ferner, Mônica Almeida, Héctor Manuel Sánchez Márquez, Yoshimi M. Rii, A. Nālani Olguin, Maureen Dewire, Kerstin Wasson

**Affiliations:** ^1^ Narragansett Bay NERR Prudence Island Rhode Island USA; ^2^ Catbird Stats, LLC Gautier Mississippi USA; ^3^ Department of Geography and Environmental Studies Saint Mary's University Halifax Nova Scotia Canada; ^4^ Wells NERR Wells Maine USA; ^5^ Great Bay NERR New Hampshire Fish and Game Department Greenland New Hampshire USA; ^6^ Waquoit Bay NERR Waquoit Massachusetts USA; ^7^ New York State Department of Environmental Conservation, Hudson River NERR Staatsburg New York USA; ^8^ Delaware NERR and National Oceanic and Atmospheric Administration, National Centers for Coastal Ocean Science Silver Spring Maryland USA; ^9^ Chesapeake Bay NERR – Maryland, Chesapeake and Coastal Service, Maryland Department of Natural Resources Annapolis Maryland USA; ^10^ Chesapeake Bay NERR – Virginia at the Virginia Institute of Marine Science Gloucester Point Virginia USA; ^11^ North Carolina Coastal Reserve and NERR Wilmington North Carolina USA; ^12^ North‐Inlet Winyah Bay NERR Georgetown South Carolina USA; ^13^ Ace Basin NERR, South Carolina Department of Natural Resources Green Pond South Carolina USA; ^14^ Sapelo Island NERR, Georgia Department of Natural Resources Sapelo Island Georgia USA; ^15^ GTM NERR Ponte Vedra Beach Florida USA; ^16^ Rookery Bay NERR Naples Florida USA; ^17^ Mission‐Aransas NERR University of Texas Marine Science Institute Port Aransas Texas USA; ^18^ Old Woman Creek NERR Huron Ohio USA; ^19^ Jobos Bay NERR Puerto Rico Department of Natural and Environmental Resources Aguirre Puerto Rico; ^20^ Center for Marine & Environmental Studies University of the Virgin Islands St. Thomas US Virgin Islands; ^21^ Kachemak Bay NERR, Alaska Center for Conservation Science University of Alaska Anchorage Homer Alaska USA; ^22^ The Nature Trust of British Columbia Nanaimo British Columbia Canada; ^23^ Padilla Bay NERR Mount Vernon Washington USA; ^24^ South Slough NERR Charleston Oregon USA; ^25^ San Francisco Bay NERR and San Francisco State University Tiburon California USA; ^26^ Tijuana River NERR Imperial Beach California USA; ^27^ Terra Peninsular Ensenada Baja California Mexico; ^28^ Heʻeia NERR, Hawaiʻi Institute of Marine Biology University of Hawaiʻi at Mānoa Kāneʻohe Hawaii USA; ^29^ Heʻeia NERR, Marine Biology Graduate Program University of Hawaiʻi at Mānoa Honolulu Hawaii USA; ^30^ Elkhorn Slough NERR Watsonville California USA; ^31^ Ecology and Evolutionary Biology, University of California Santa Cruz California USA

## Abstract

The crucial role of coastal wetlands supporting diverse terrestrial wildlife is often asserted but has not been demonstrated in broad‐scale field evaluations; a comprehensive assessment of wildlife use of these vital ecosystems is therefore needed. Our goal was to conduct the first coordinated assessment of terrestrial wildlife across North America's vegetated coastal wetlands. We elucidated spatial patterns related to geographic and landscape differences and temporal patterns of wildlife diversity and abundance. Using camera traps deployed with a consistent methodology across 25 National Estuarine Research Reserves and 7 additional sites in North America, we documented 146 species (104 birds, 36 mammals, 6 herpetofauna) using wetlands for foraging, resting, and as nursery habitat. Most species were native, though non‐native species dominated island sites. Wetlands with greater landscape heterogeneity attracted distinctive wildlife assemblages, as did wetland–upland ecotones. Many species, particularly mammals, used wetlands almost exclusively at night, and wildlife abundance was low when wetlands were flooded. Our findings demonstrate the significant role coastal wetlands play in wildlife support, a service that may decline with accelerating sea‐level rise. This coordinated approach offers a model for broad‐scale wildlife studies and highlights the importance of incorporating top‐down perspectives and a landscape approach into coastal conservation planning.

## Graphical abstract

We conducted the first coordinated assessment of terrestrial wildlife across North America's vegetated coastal wetlands. Our study demonstrates the significant role coastal wetlands play in wildlife support, offers a model for broad‐scale wildlife studies, and highlights the importance of incorporating top‐down perspectives and a landscape approach into coastal conservation planning.

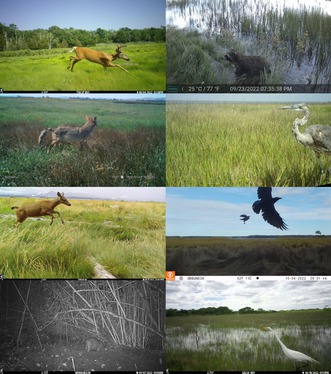

## 1 Introduction

Coastal wetlands are of great economic value (Mazzocco et al. 2022; Wang et al. 2022), providing ecosystem services such as coastal protection, carbon sequestration, fisheries enhancement, and human recreation (Adams et al. 2021; Barbier et al. 2011; zu Ermgassen et al. 2021). Despite their value, extensive coastal wetlands have been lost, in some regions reduced by as much as 80% (Gedan et al. 2009; Goldberg et al. 2020). Conservation and restoration of coastal wetlands have mostly applied a bottom‐up approach, focusing on dominant plants and the physical factors that support them, including restoring hydrology (Besterman et al. 2022; Burdick and Roman 2012) and raising elevation via sediment addition (Raposa et al. 2022; Thorne et al. 2019).

While most restoration efforts have focused on bottom‐up approaches, there is increasing recognition that top‐down factors can play a very strong role in shaping coastal wetland resilience (Bertness and Silliman 2008). Many studies have focused on small invertebrates with strong effects on wetland resilience, such as crabs (Crotty et al. 2020) and snails (Silliman et al. 2005) driving marsh die‐back. And yet vertebrates can also have strong direct effects on coastal wetlands through grazing and trampling (Davidson et al. 2017; Fischman et al. 2023; Motta et al. 2020; Sharp and Angelini 2019). Vertebrates can also indirectly affect coastal wetland vegetation through trophic cascades (Atwood and Hammill 2018; Nifong and Silliman 2013). Nevertheless, the impacts of wild megafauna on coastal vegetation remain relatively understudied (Gaskins et al. 2020). Terrestrial mammals also can function as predators in the intertidal zone and serve an important function transferring energy from marine to terrestrial systems (Carlton and Hodder 2003).

In addition to having strong effects on coastal wetland ecosystems, populations of vertebrate animals can also be strongly affected by the condition and extent of wetlands. Wildlife support is often listed as a key ecosystem service of coastal wetlands (Barbier et al. 2011). While there have been some literature reviews of vertebrate diversity in coastal wetlands (Canepuccia et al. 2023; Greenberg and Maldonado 2006), there actually have been very few field characterizations of combined mammal and bird use of marshes, and none at a continental scale. Understanding wildlife use of wetlands is critical for informing management strategies. For species that have obligate associations with coastal wetlands, loss of their wetland habitat due to factors such as accelerated sea‐level rise will have strong negative consequences (Krebs et al. 2023; Roberts et al. 2019; Smith et al. 2014) that must be considered in conservation planning. Management planning also should incorporate an understanding of how invasive animal species use coastal wetlands, such as nutria ( *Myocastor coypus* ) or feral pigs ( *Sus scrofa* ) in marshes (Sasser et al. 2018; Sharp and Angelini 2019) or black rats ( *Rattus rattus* ) or green iguanas ( *Iguana iguana* ) in mangroves (Govender et al. 2012; Harper et al. 2014). Conservation planning to support declining populations of keystone species also should incorporate their use of wetlands; for instance, coastal forests can be critical for black bears ( *Ursus americanus* ) (Helfield and Naiman 2006), and coastal marshes for diamondback terrapins ( *Malaclemys terrapin* ) (Selman et al. 2019).

We conducted a coordinated study of wildlife use of vegetated North American coastal wetlands—the first broad‐scale, multi‐taxa field investigation of vertebrates other than fish using tidal marshes and other coastal wetlands (hereafter, we use the term ‘wildlife’ to specifically refer to mammals, birds, and herpetofauna). We used non‐invasive camera traps and a standardized sampling protocol at sites across the U.S. National Estuarine Research Reserve System (NERRS), as well as at selected partner sites. The NERRS is an ideal platform for evaluations of coastal wetland resilience (Endris et al. 2024; Raposa et al. 2016; Stevens et al. 2023), and while crab abundance and communities have been evaluated across the NERRS (Wasson et al. 2019), vertebrate wildlife communities have not. Thus, one goal of our study was to provide the first characterization of the diversity of terrestrial vertebrates using coastal wetlands in North America. We examined wildlife community composition and abundance across wetland types and regions and documented different ways wildlife use these wetlands.

A second goal of our study was to examine spatial patterns in terrestrial wildlife occurrence across different scales. At the broadest scale, we compared wildlife diversity in different regions across North American coasts. At a local scale, we explored various hypotheses about possible correlates of wildlife abundance and diversity. We hypothesized that sites with open water and/or pannes and vegetation support more wildlife species and higher abundance than uniformly vegetated areas because landscape heterogeneity provides opportunities for different species (McKinney et al. 2009; Pianka 1966). We also hypothesized that the ecotones between wetlands and uplands concentrate wildlife and have higher abundance and diversity than wetlands themselves (Kark and van Rensburg 2006).

A third and final goal of our study was to examine temporal variation in terrestrial wildlife use of wetlands. We hypothesized that some animals avoid marshes by day, due to real predation risk or real or perceived risk from humans (Casazza et al. 2012). We also predicted that many animals would avoid marshes during periods of inundation, due to a decrease in foraging or resting opportunities or increased risk of drowning (Smith et al. 2014). Understanding species‐specific use of wetlands across diel and tidal cycles is important to design future studies in ways to optimize detections, and for wildlife management.

In summary, we conducted the first coordinated assessment of terrestrial vertebrate wildlife across North America's coastal wetlands. We collected and synthesized camera‐trapping data from 32 sites to elucidate spatial patterns related to geographic and landscape differences, as well as temporal patterns of wildlife diversity and abundance. Since vertebrate animals affect—and are affected by—coastal wetlands, this is a critical first step towards understanding wildlife biodiversity in these ecosystems.

## 2 Methods

### 2.1 Study Sites

**FIGURE 1:**
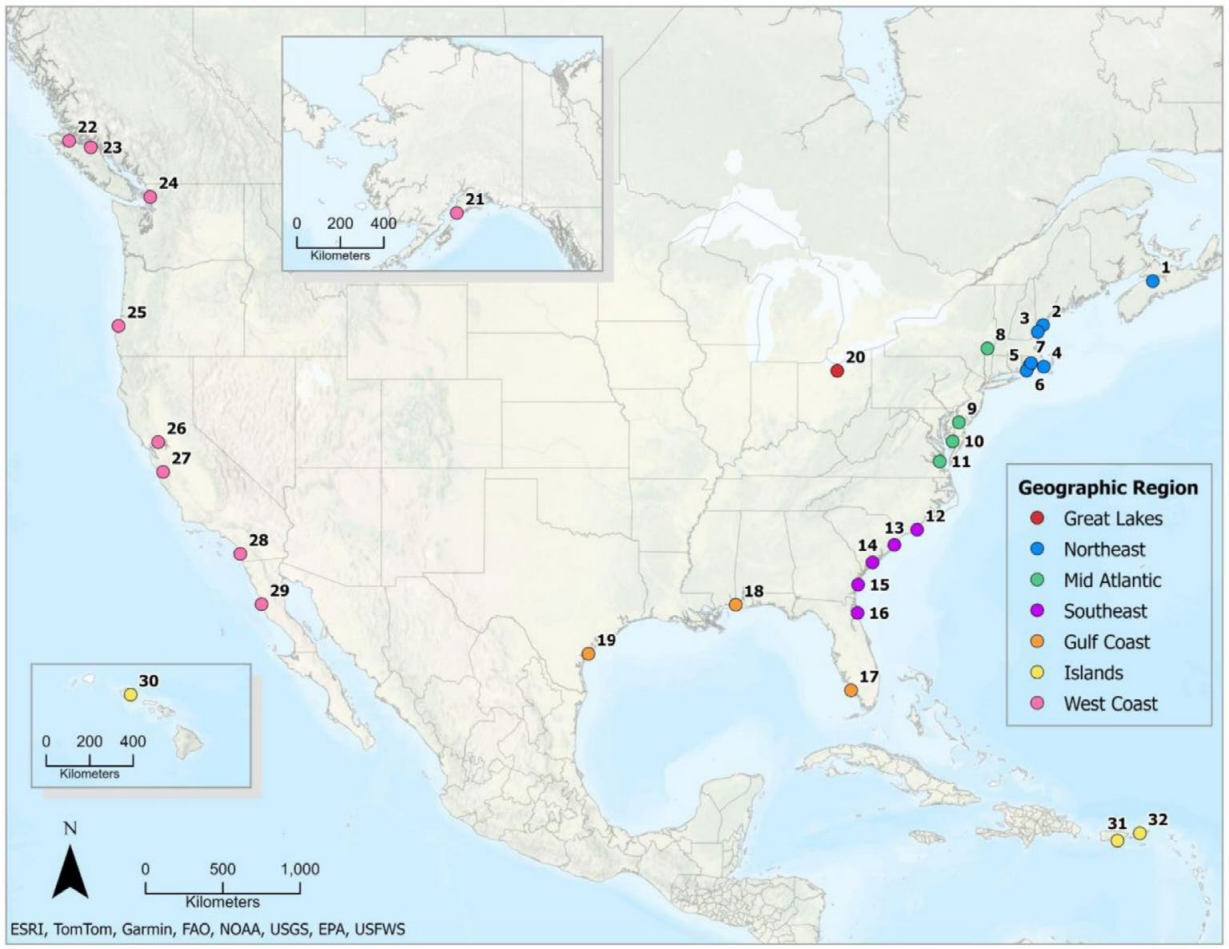
Map of the 32 sites included in this continent‐wide camera‐trap study. Sites are grouped into geographic regions, with number codes corresponding to site names shown in Appendix S1.

**FIGURE 2:**
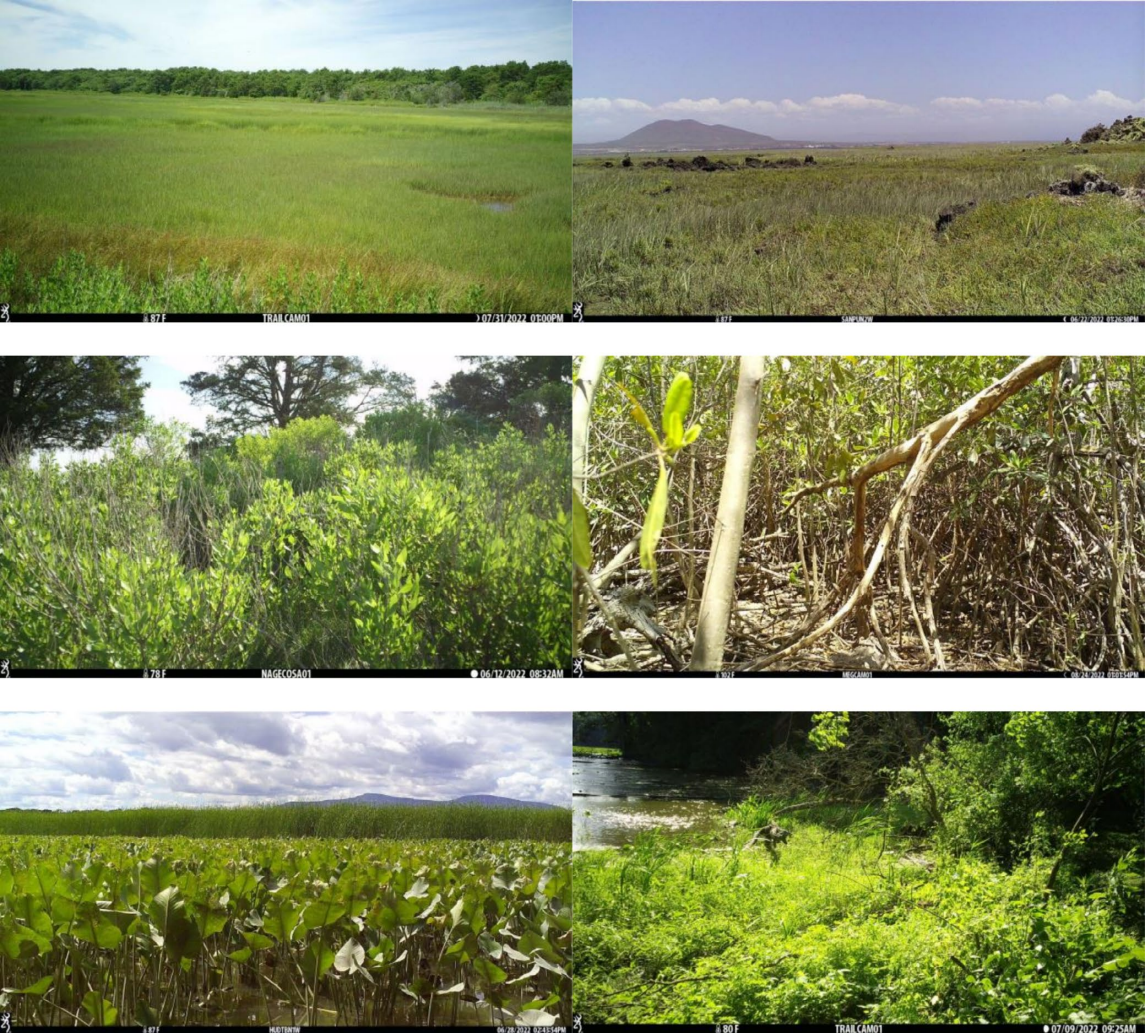
Representative images of wetland types where camera trapping was conducted in this study, including (from top‐left) East Coast salt marsh (Waquoit Bay MA NERR), West Coast salt marsh (Reserva Natural Punta Mazo Mexico), Ecotone (Narragansett Bay RI NERR), mangrove (Magen's Bay, St. Thomas VI), tidal freshwater (Hudson River NY NERR), and nontidal freshwater (Old Woman Creek OH NERR) wetlands. Images are from a continent‐wide study using camera traps in 2022.

Wildlife data were collected from diverse vegetated coastal wetlands across North America. Here, we define a site as the general location within or associated with one of the NERRs or partner organizations that participated in our study and a station as a discrete location at a site where a camera trap was deployed. Data were collected from 32 sites (25 in the NERRS) distributed across seven major geographic regions defined by NERRS convention (Figure 1). In total, 109 camera trap stations were established across the sites; 74% of stations were located in salt marsh, 7% in mangrove, and 4% in tidal and nontidal freshwater marsh coastal wetlands, with the remaining 15% of stations in ecotones adjacent to the wetlands (Table 1, Figure 2).

**TABLE 1:** Summary of the scope of a continent‐wide study of wildlife in coastal wetlands using camera traps in 2022. Wetland cover values are means across camera trap stations. See Appendix S1 for more details.

Category	Parameter	Number
Geography	# geographic regions	7
	# sites	32
	# camera trap stations	109
Wetland type	# salt marsh stations	81
	# mangrove stations	8
	# freshwater marsh stations	4
	# wetland‐upland ecotone stations	16
Wetland cover	Mean vegetation % cover	89
	Mean water % cover	6
	Mean bare % cover	5
Effort	Total hours sampled	202,146
	Mean hours sampled/station	1855
Cameras	# stations with Browning	74
	# stations with Bushnell	21
	# stations with other	14

### 2.2 Experimental Design

We developed and implemented a standardized protocol to foster consistency when establishing stations and conducting field work. Stations were located within vegetated wetlands, near (within 2 m) the landward boundary, with cameras facing the water to capture wildlife using the coastal vegetation. Small areas of land‐locked pocket marsh that might concentrate wildlife and inflate wildlife abundance estimates were avoided. Stations were only established adjacent to natural uplands (developed upland edges were avoided) and in areas with > 70% wetland vegetation cover within the camera trap viewfield (i.e., < 30% water or bare ground). Almost all camera trap stations were established as described above, but a few deviated somewhat from this design due to local conditions (Appendix S1 in Supporting Information). Camera trap stations were always a minimum distance of 100 m apart to help ensure data independence.

The target was for each site to establish at least three camera trap stations in wetlands, but this varied depending on camera availability. Ultimately, 24 of 32 sites deployed 3–5 wetland camera traps, 7 sites deployed 2 wetland traps, and 1 site deployed 1 wetland trap. Sites with more than three camera traps available could optionally deploy the additional traps in other wetland locations or use them to collect data from ecotones adjacent to the wetland. Ecotone cameras were always paired with a pre‐existing wetland camera (one facing landward, the other seaward at the same station). Initially, 148 camera traps in total were deployed across the 32 sites but 39 of these stations were not used in the study due to one or more issues that compromised camera effectiveness and rendered data suspect. This resulted in 109 camera trap stations in our study, with 93 wetland cameras across all sites and 16 ecotone cameras across 8 sites (Table 1; Appendix S1).

### 2.3 Camera Trapping and Image Processing

Camera trapping occurred during summer 2022. Most sites were sampled within the June 1–September 30 window, although two sites (San Francisco Bay NERR and Tijuana River NERR in California) started late and continued sampling into October 2022 (at these sites, peak temperatures occur in late summer/early fall). All camera traps collected data for a minimum of 3 weeks within the sampling window, but almost every station was deployed for longer than this, with just one camera deployed for slightly less than 3 weeks (mean deployment = 77 days; range = 20–122 days; Appendix S1). In the field, each camera was deployed at a target of 1 m above the wetland surface to help minimize false triggers from moving wetland vegetation.

Image processing was conducted by the same four staff members at the Narragansett Bay NERR for consistency. All image interpretation and wildlife identification were done manually for each individual image. Independent detections were defined using a 30 min window between consecutive images of the same species (window duration varies among species and studies; we chose a common intermediate value because we were dealing with many diverse species [Burton et al. 2015; Lepard et al. 2018]). Additional images of the same species within this window after the first image was taken were removed. We also removed all images of humans and of dogs clearly associated with humans (e.g., being taken for a walk). Finally, for each photo with wildlife we recorded if the wetland surface was flooded by tidal water or dry and if it was day, crepuscular (dawn/dusk), or night for later temporal analyses. For additional information specific to camera trapping methods (number and type of camera models used, programming setting, detailed methods used for deploying camera traps in the field) and processing of collected images, see Appendix S2.

### 2.4 Data Analysis

We focused analyses on a few key wildlife metrics. For each species, we calculated the relative abundance index (RAI) as the number of detections per 100 camera trapping days (detections/total camera days × 100) as an indicator of relative species abundance (Jenks et al. 2011; O'Brien et al. 2003; Palmer et al. 2018), and naïve occupancy (the proportion of sites where a species was present) as an indicator of spatial distribution and extent (Rovero et al. 2016). For wildlife communities, we focused on community composition, richness (S; count of species per station or site), and total RAI (all species combined). The specific metrics and data used varied for each analysis conducted (Table S2.2).

To examine broad patterns in wildlife biodiversity across the entire study, we tallied overall and site‐specific richness and, for each species, calculated mean RAI (across all sites) and naïve occupancy. We used similarity percentages (SIMPER), which accounts for both relative abundance and frequency of occurrence, to identify species and families that most typified wildlife communities across the entire study and within each geographic region (Clarke and Gorley 2015); analyses were conducted using square root‐transformed data with sites as replicates (RAI for each species/family was averaged across stations within each site). We also quantified the prevalence of U.S. federally listed threatened and endangered species as an indicator of conservation status, apex and mesopredators, all native and non‐native species (i.e., not present in North America prior to European colonization) and a subset of feral/domesticated non‐natives at sites and across the entire study.

To compare species composition of wildlife communities among regions, we used two‐way nested PERMANOVA, which is a non‐parametric alternative to multivariate ANOVA (site as random factor nested within region as fixed; Table S2.2). Tests were performed using Bray–Curtis resemblance matrices and species RAI data that were square‐root transformed to reduce the influence of highly‐abundant species; significant results were followed by pairwise comparisons. Non‐metric multidimensional scaling (nMDS) was then used to visualize site and region associations in two‐dimensional space based on wildlife community similarities among stations. Kruskal–Wallis tests, using the median of untransformed station values to represent each site, were used to compare richness and total RAI (all species combined) among regions. When a significant difference (*p* < 0.05) among regions was identified, a post hoc Dunn's test was used to examine pairwise differences. Last, maps were made to illustrate patterns in the distribution of major species across sites and regions.

For within‐site spatial analyses, we used two‐way crossed PERMANOVA (site as random factor, landscape heterogeneity as fixed factor) to compare community composition between homogeneous (vegetation only) and heterogeneous (vegetation intermixed with pools, pannes, or bare areas) landscapes. Species RAI data were used from seven sites that had stations in both homogeneous and heterogeneous landscapes and the model was run using square‐root‐transformed data with stations as replicates and site and landscape heterogeneity as factors. Paired Wilcoxon tests (also known as Wilcoxon‐signed rank tests) were used to compare species richness and total RAI between homogeneous and heterogeneous landscapes, with the median value of stations for each landscape type within a site representing the site. These same analyses were also used for a second spatial analysis comparing wildlife between wetlands and adjacent ecotones, using data from seven sites that collected data in both environments.

Temporal analyses focused on comparing wildlife across different diel (day, night, crepuscular) and tidal (wetland surfaces flooded or dry) periods. Two‐way crossed PERMANOVA, Kruskal–Wallis/Dunn's tests (diel), and paired Wilcoxon tests (tidal) were used as described above for spatial analyses except using raw species counts for abundance instead of RAI (we did not track camera trapping effort for different diel and tidal periods and were thus unable to calculate RAI). Even though they do not account for differences in camera‐trapping effort, analyses using count data can be useful for illustrating absolute differences in richness and abundance between diel and tidal periods.

All PERMANOVA tests were performed using stations as replicates and we report results for fixed factors only; all other tests used sites as replicates. Community‐level multivariate analyses and visualizations were performed using PRIMER 7.0 (Clarke and Gorley 2015). Nonparametric tests were performed in R version 4.2.2 (R Core Team 2022), using the “stats” package in base R for Kruskal–Wallis and Wilcoxon tests and the “FSA” package v. 0.9.5 (Ogle et al. 2023) for Dunn's tests. Paired box plots to illustrate paired Wilcoxon results were made with the “ggpubr” package v. 0.6.0 (Kassambara 2023). All other figures were created with SigmaPlot 15.0 (Inpixon) and maps were made using ArcGIS Pro 3.2 (ESRI).

## 3 Results

### 3.1 Biodiversity

A species‐rich wildlife community was documented in North American vegetated coastal wetlands, with 146 total species identified across all wetland and wetland‐upland ecotone stations (Appendix S1). The community was dominated by birds (104 species), followed by mammals (36) and herpetofauna (6).

**FIGURE 3:**
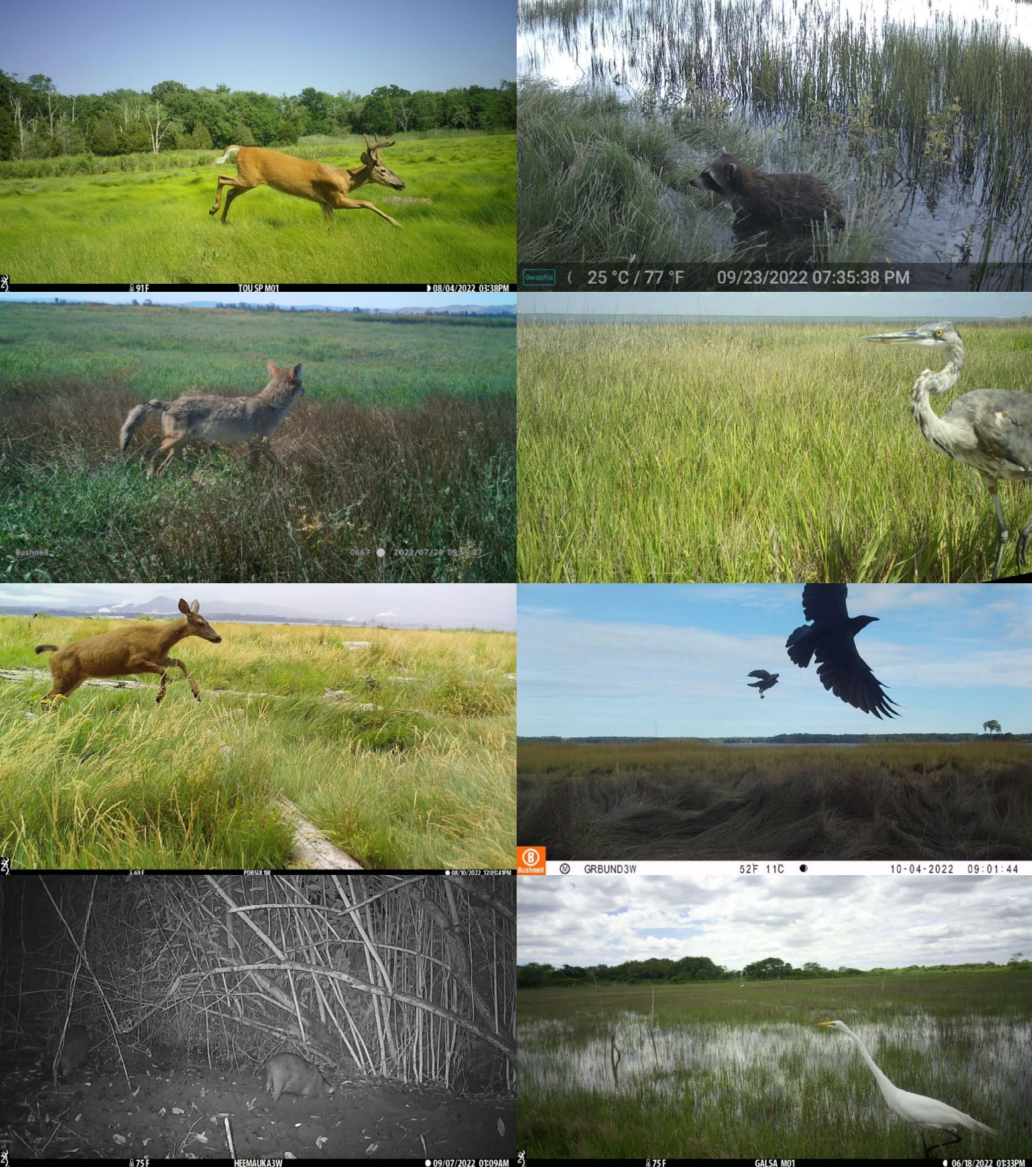
Example images of species that typify North American coastal wetlands. From top‐left: White‐tailed deer (Touisset Refuge RI), raccoon (North‐Inlet Winyah Bay NERR SC), coyote (San Francisco Bay NERR CA), great blue heron (Mission‐Aransas NERR TX), Columbian black‐tailed deer (Padilla Bay NERR WA), Corvus spp. (Great Bay NERR NH), feral pig (Heʻeia NERR HI), and great egret (Galilee Bird Sanctuary RI). Images are from a continent‐wide study using camera traps in 2022.

**FIGURE 4:**
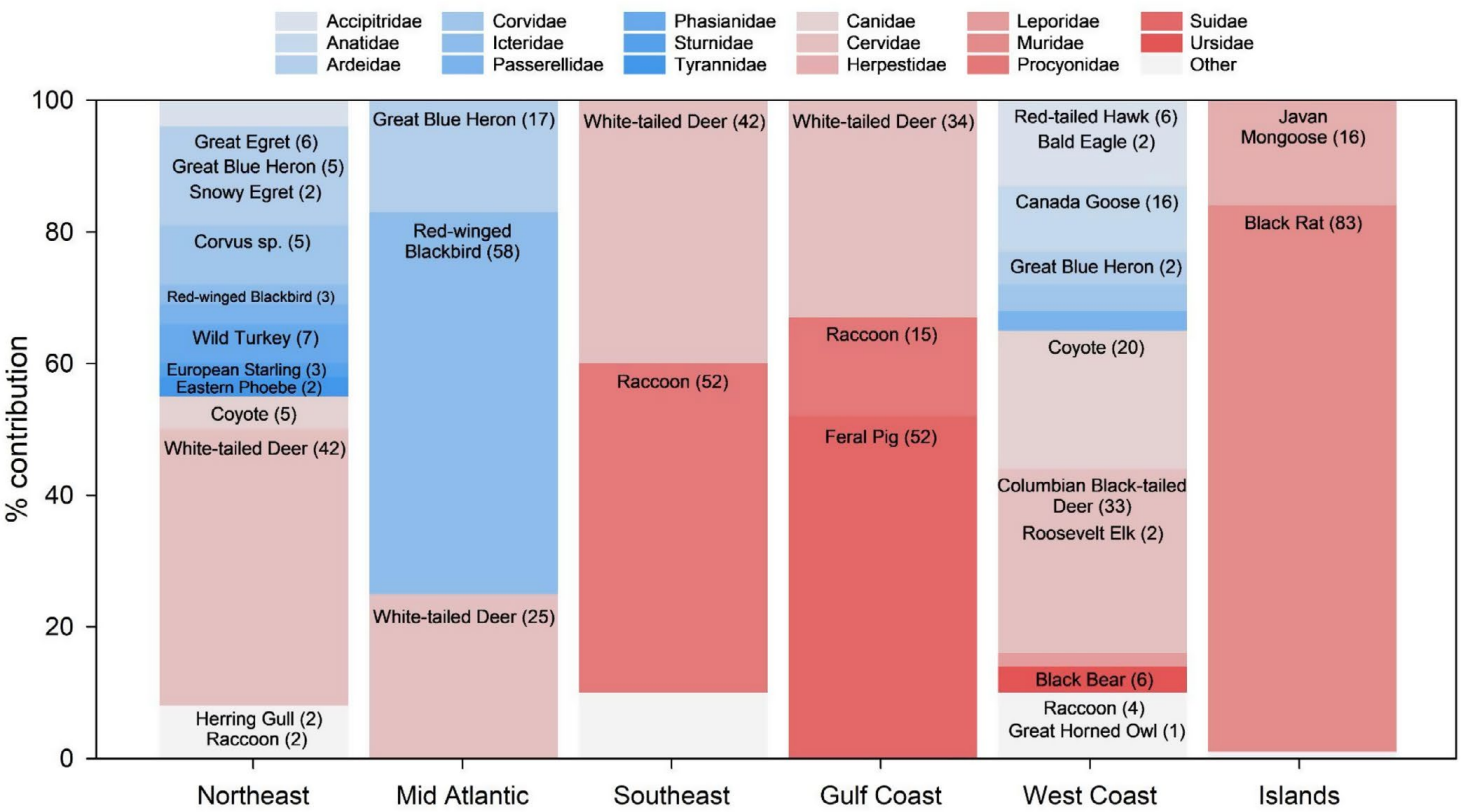
Characteristic wildlife in North American coastal wetlands. Stacked bars are % contributions of families to wildlife community similarity for each geographic region in this study from SIMPER analysis. Superimposed on each bar are the % contributions of individual species (from a separate SIMPER analysis) to community similarity for each region, with species listed within their representative family. Birds (blue shades) are important components of Northeast, Mid Atlantic and West Coast wetland wildlife communities whereas mammals dominate in other regions. Only species/families contributing to 90% total similarity are shown, resulting in some bar sections remaining unlabeled. Great Lakes region (just one site) not shown. See Appendix S1 for scientific names. Data are from a continent‐wide study using camera traps in 2022.

When considering wetlands only (i.e., no ecotones) 129 species were observed across all sites, with a median richness of 9 species/site (range = 1–25). Averaged across all sites, most species were present at very low abundances, but a few were highly abundant, including white‐tailed deer ( *Odocoileus virginianus* ; mean RAI = 14.17), raccoon ( *Procyon lotor* ; 9.67), European starling ( *Sturnus vulgaris* ; 8.0), feral pig (5.0), Javan mongoose (*Urva javanica*; 4.90; extremely abundant at Heʻeia NERR only), and Canada goose ( *Branta canadensis* ; 3.82) (Appendix S1). Site occupancy of most species was also very low (80% of species were found at three or fewer sites), but a few widely‐distributed species were found in wetlands at many sites, including white‐tailed deer (53% of sites), raccoon (44%), coyote ( *Canis latrans* ) and great egret ( *Ardea alba* ; 41% each), and great blue heron ( *Ardea herodias* ; 38%). From SIMPER, the wildlife species that most typify North American coastal wetlands are white‐tailed deer (contributed 37% to overall community similarity), raccoon (12%), coyote (9%), great blue heron (7%), Columbian black‐tailed deer ( *Odocoileus hemionus columbianus* ) and corvid spp. (4% each), and feral pig and great egret (3% each) (Figures 3 and 4).

Many of the species using coastal wetlands were predators (Appendix S3). Mammalian apex predators (gray wolf [ *Canis lupus* ], black bear, mountain lion [ *Puma concolor* ]) were only found at sites in the Pacific Northwest, but mammalian mesopredators (e.g., coyote, bobcat [ *Lynx rufus* ], raccoon) were widely distributed, with at least one mesopredator species found at 75% of wetland sites (Appendix S1). Avian predators (raptors, order Accipitriformes, and owls, order Strigiformes) were also common, with at least one species found at 53% of sites, almost exclusively in the Northeast, Mid‐Atlantic and West Coast regions. From SIMPER, the predators that most typify North American coastal wetlands are raccoon (contributed 12% to overall community similarity), coyote (9%), red‐tailed hawk ( *Buteo jamaicensis* ; 2%), and great‐horned owl ( *Bubo virginianus* ) and black bear (1% each).

Herbivorous and omnivorous mammals were near‐ubiquitous in coastal wetlands, with a site occupancy of 84% (present in 27 of 32 sites; Appendix S1). Herbivores were primarily deer, rabbits, and various rodents, but SIMPER identified white‐tailed deer (contributed 37% to community similarity across all sites), Columbian black‐tailed deer (4%), and feral pig (3%) as the most characteristic herbivorous species using coastal wetlands.

Native species dominated communities in most coastal wetlands; 88% of all species detected were native to North America. Most (19 of 29) continental sites were composed entirely of native species, but the inverse was true in mangroves at two island sites; 12 of 13 wildlife species detected at Heʻeia NERR were not originally native to Hawaiʻi, and three of five species at the U.S. Virgin Islands site were non‐native. Overall, non‐native species were found at just 12 sites (Appendix S1). Feral domestic species (a subset of non‐natives including pigs, cows [ *Bos taurus* ], dogs [ *Canis lupus familiaris* ], and cats [ *Felis catus* ]) were found at nine sites overall and half the sites along the U.S. Southeast and Gulf coasts (Appendices S1 and S3). Feral pigs in particular were highly abundant at GTM NERR FL, Grand Bay NERR MS, and Heʻeia NERR HI. None of the species found in North American coastal wetlands were federally listed threatened and endangered (U.S. Fish & Wildlife Service, Environmental Conservation Online System), although the saltmarsh sparrow (*Ammospiza caudacuta*; found at the Galilee and Touisset RI sites) is under review by the U.S. Fish & Wildlife Service to make a listing determination (https://www.fws.gov/species/saltmarsh‐sparrow‐ammodramus‐caudacutus).

### 3.2 Functions of Wetlands for Wildlife

**FIGURE 5:**
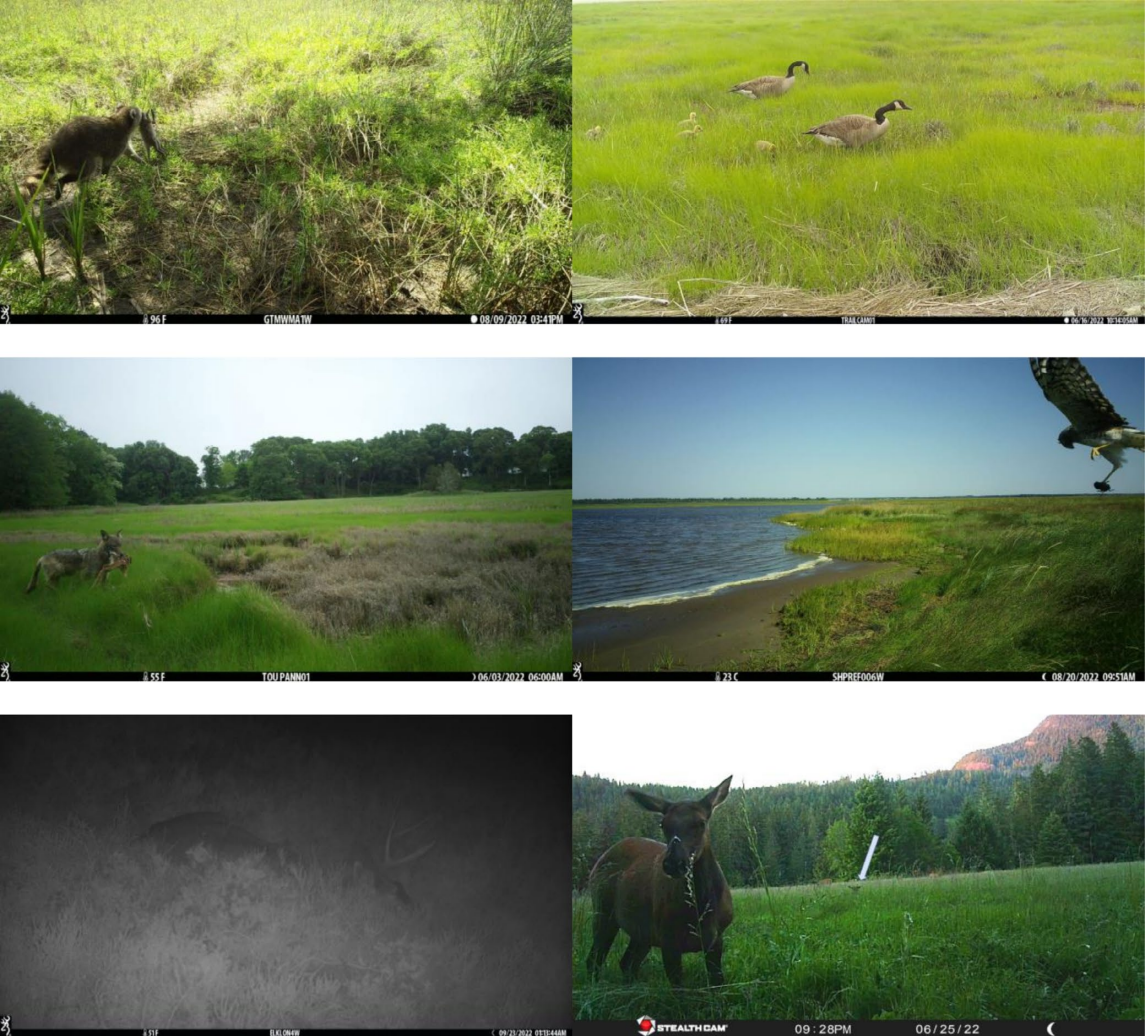
Wildlife use coastal wetlands as a nursery to raise young (top row; raccoon carrying young at GTM NERR FL and Canada goose with goslings at Wells NERR ME), to forage for food (middle row; coyote with prey at Touisset Refuge RI and northern harrier [ *Circus cyaneus* ] with prey in Atlantic Canada), and as a refuge and to rest (bottom row; Columbian black‐tailed deer resting at Elkhorn Slough NERR CA and Roosevelt elk [ *Cervus elaphus canadensis* ; white line] resting at Salmon River BC Canada). Images are from a continent‐wide study using camera traps in 2022.

Our study demonstrated that wildlife species use coastal wetland vegetation for a variety of key functions (Figure 5; Appendix S3). We detected many animals foraging in coastal wetlands, such as raptors and ardeids hunting and coyotes obtaining small prey. Camera trapping also revealed coastal wetlands serving as a nursery, including for white‐tailed deer, raccoon, bobcat, and Canada goose. Numerous observations also provided evidence of coastal vegetation used as refuge and resting habitat.

### 3.3 Wildlife Variability Among Regions

**FIGURE 6:**
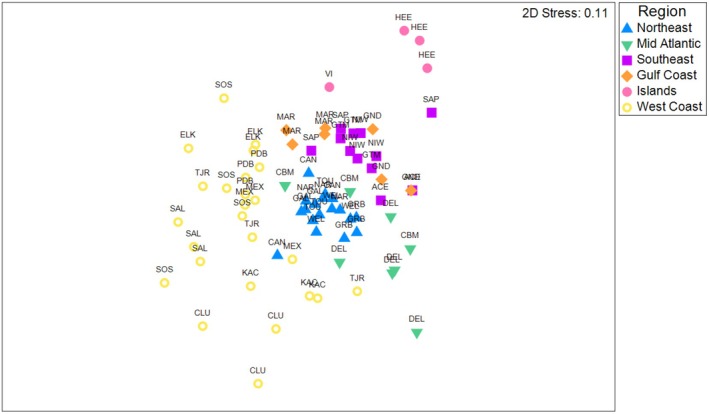
nMDS plot of wetland wildlife community similarity among geographic regions based on species composition. Points are individual camera trap stations. Symbols represent regions sampled in this study and are labeled with three‐digit site codes (Appendix S1). Plot is scaled for clarity (two outlier stations, one each at SOS and VI, not visible). Results indicate that coastal wetlands in most North American regions support distinct wildlife communities (PERMANOVA, *p* = 0.001). Data are from a continent‐wide study using camera traps in 2022.

**FIGURE 7:**
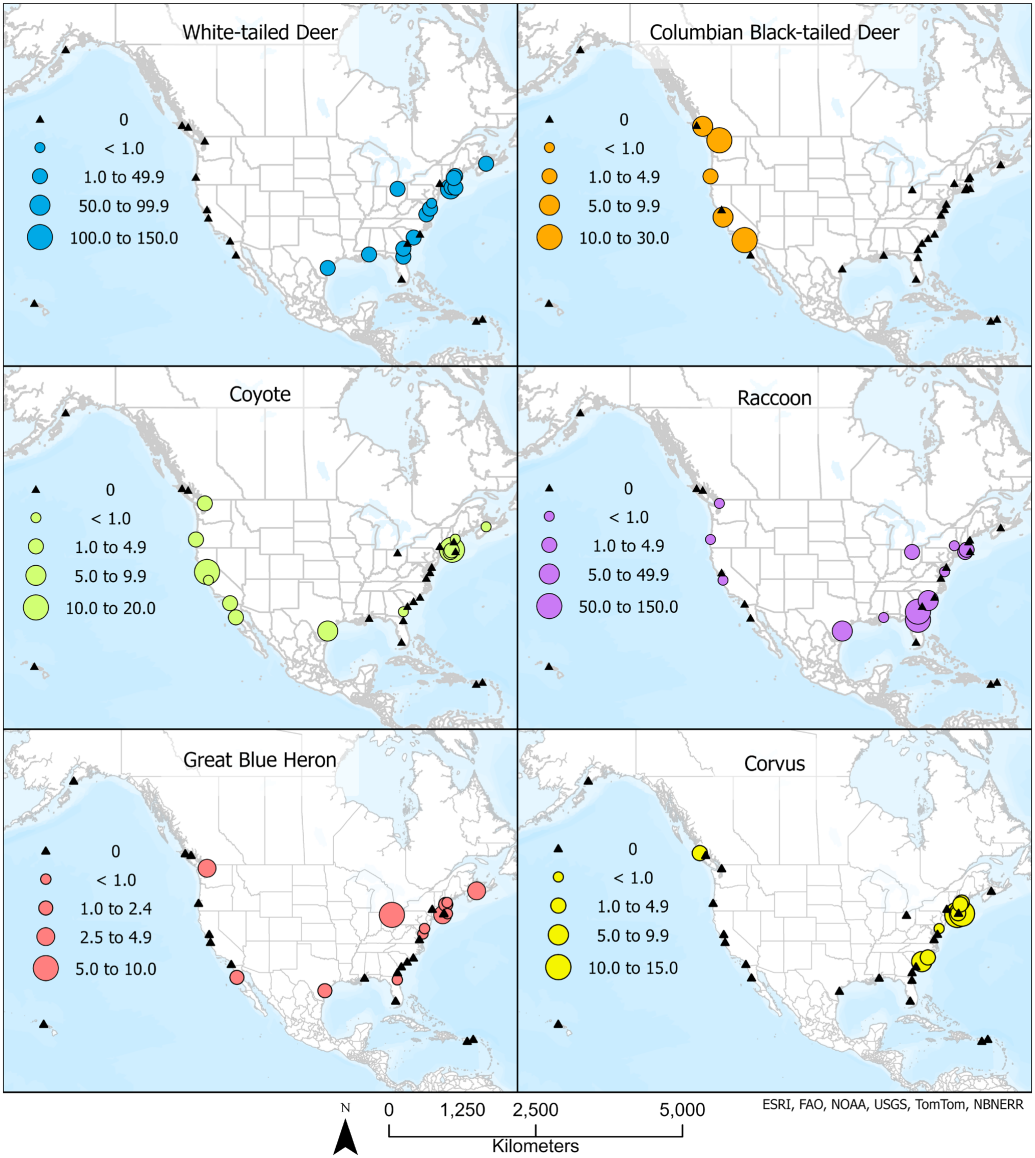
Distribution and abundance (RAI) of key wildlife species in North American coastal wetlands. Distributions shown for the top six species (white‐tailed deer, raccoon, coyote, great blue heron, Columbian black‐tailed deer, and Corvus spp.) identified with SIMPER as most characteristic of the wetlands across all study sites. Data are from a continent‐wide study using camera traps in 2022.

Wildlife species composition in coastal wetlands was significantly different across North American geographic regions (PERMANOVA, *p* = 0.001) with all regional pairwise comparisons significant (*p* < 0.05) except Mid‐Atlantic vs. Islands (*p* = 0.15) and Gulf Coast versus Mid Atlantic (*p* = 0.18), Southeast (*p* = 0.79), and Islands (*p* = 0.19). Separation between West Coast, Island/mangrove and most East Coast sites was especially apparent (Figure 6). These differences were due in part to varying geographic distributions of key species, such as disparate overall distributions of white‐tailed and black‐tailed deer and an inverse pattern for coyote and raccoon, particularly in the southeast (i.e., where the abundance of one was high, the other was low; Figure 7). SIMPER analysis indicated that although characteristic species varied among regions, many wetlands in our study were dominated by the same major groups of wildlife, primarily families Cervidae, Ardeidae, Canidae, and Procyonidae (Figure 4). Exceptions were mangrove wetlands at island sites, which were dominated by non‐native species in families Muridae and Herpestidae.

There was a significant difference in species richness among regions (Kruskal–Wallis, *χ* ^2^ = 19.1, df = 5, *p* = 0.0018) with higher richness in the Northeast compared to the Mid Atlantic, Southeast, and West Coast (Dunn's test, *p* < 0.05). Total abundance was also significantly different among regions (Kruskal–Wallis, *χ* ^2^ = 16.7, df = 5, *p* = 0.005) with higher abundance in the Northeast compared to the Mid‐Atlantic and West Coast (Dunn's test, *p* < 0.05).

### 3.4 Comparing Wildlife Between Homogeneous Versus Heterogeneous Landscapes

**FIGURE 8:**
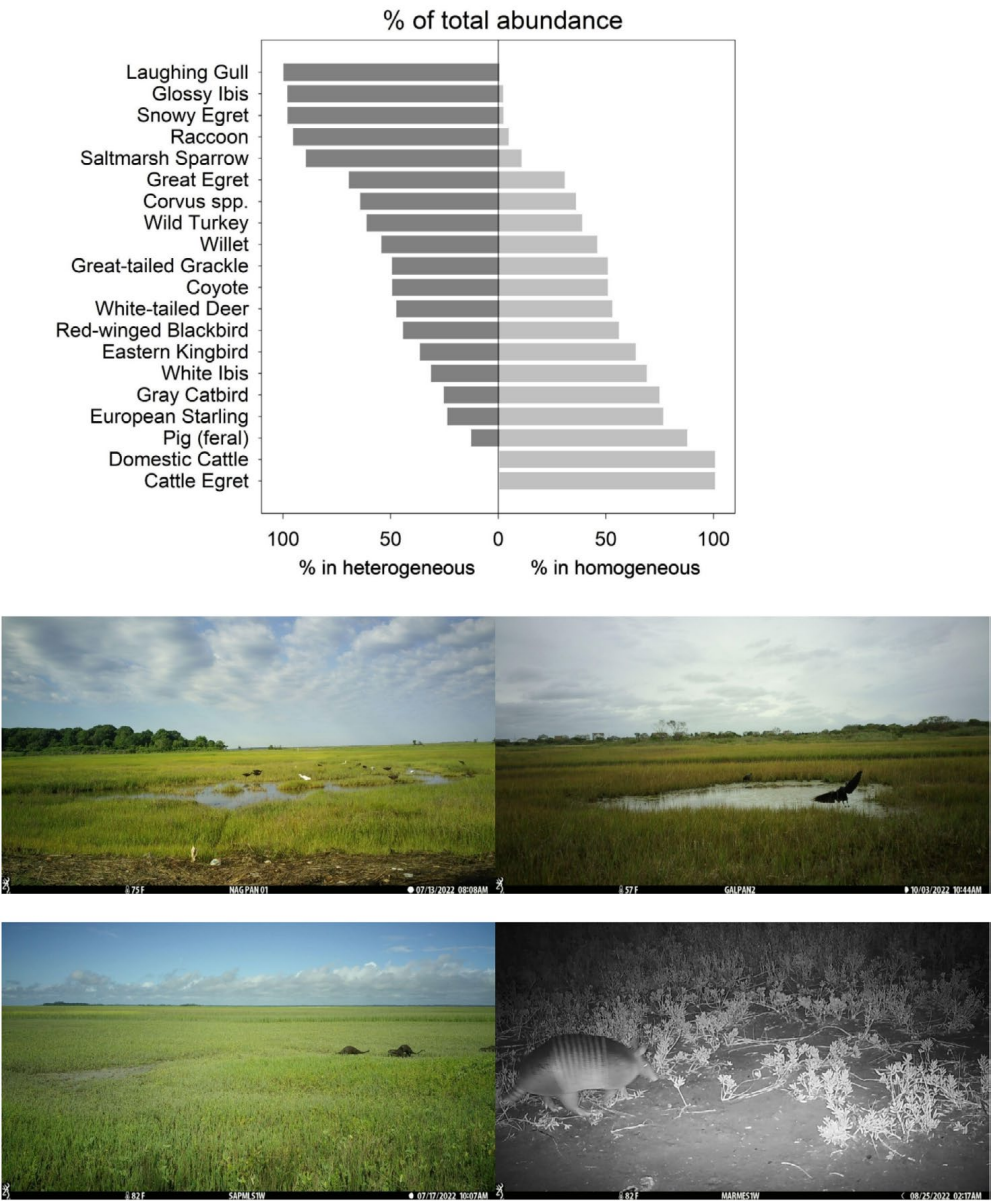
Effects of landscape diversity on wildlife in coastal wetlands. Top: Relative (%) abundance of wildlife in heterogeneous (vegetation with water or bare) and homogeneous (vegetation only) landscapes across seven sites that collected data in both types (see Appendix S1 for scientific names). Bottom: Wildlife using heterogeneous wetland landscapes (from top‐left: Glossy ibis and snowy egret at Narragansett NERR RI, Corvus sp. at Galilee Bird Sanctuary RI, river otter [ *Lontra canadensis* ] at Sapelo Island NERR GA, and nine‐banded armadillo [ *Dasypus novemcinctus* ] at Mission‐Aransas NERR TX). Data and images are from a continent‐wide study using camera traps in 2022.

The abundance of some species was markedly different in heterogeneous (vegetation mixed with pannes, pools, or bare ground) versus homogeneous (vegetation only) landscapes in the wetlands. Raccoon, glossy ibis ( *Plegadis falcinellus* ), snowy egret ( *Egretta thula* ), laughing gull ( *Leucophaeus atricilla* ), and saltmarsh sparrow in particular were much more abundant in heterogeneous landscapes and were often observed foraging in or on the edge of pannes, pools, and bare areas (Figure 8). Overall, however, wildlife species composition was not significantly different between homogeneous and heterogeneous landscapes (PERMANOVA, *p* = 0.47). Total abundance (paired Wilcoxon, *V* = 19, *p* = 0.45) and richness (paired Wilcoxon, *V* = 21, *p* = 0.27) were also not significantly different between the two landscape types (Figure S2.2).

**FIGURE 9:**
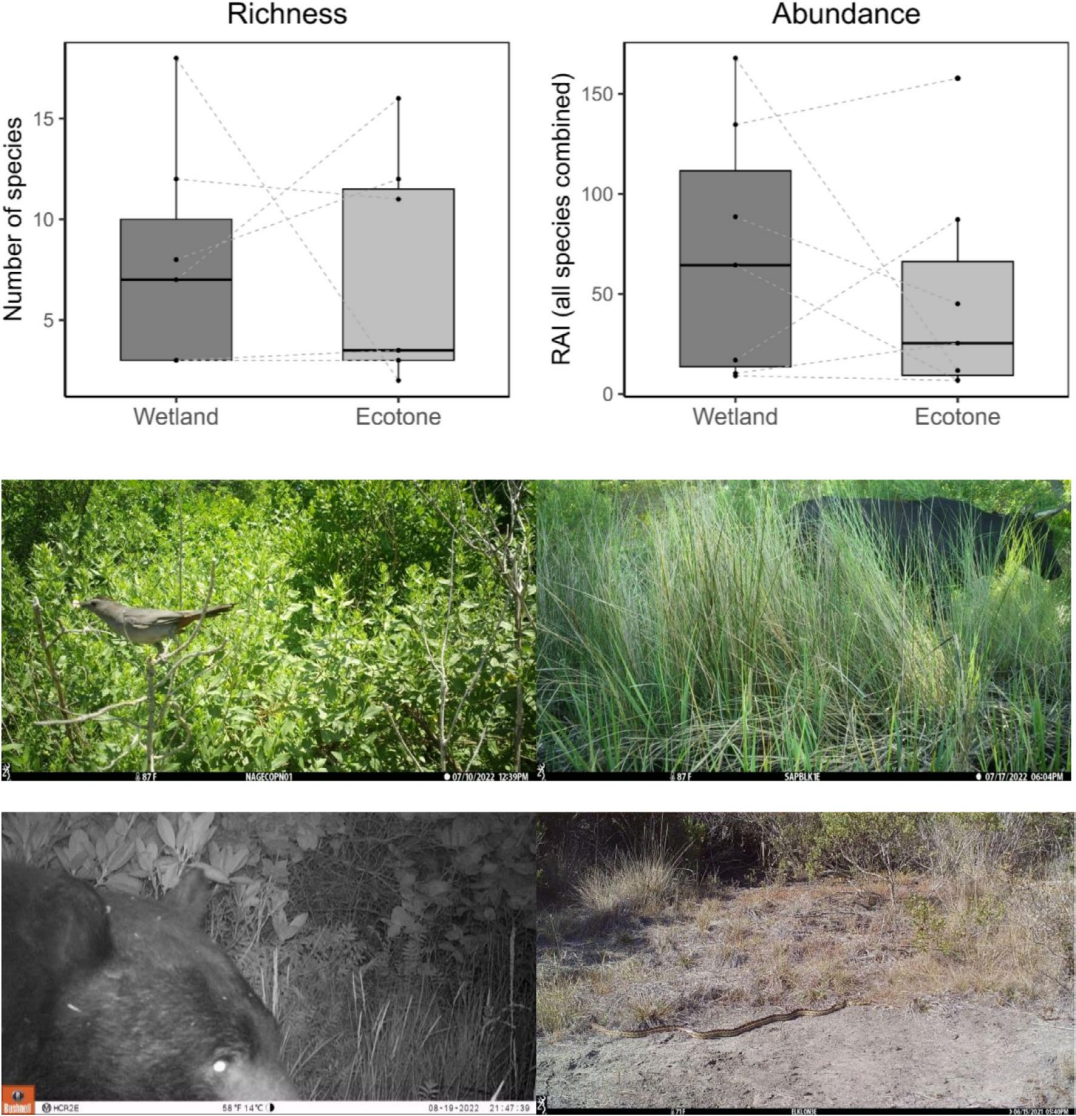
Wildlife use of coastal wetlands and adjacent ecotones. Top: Paired box plots for species richness and total abundance (as RAI) in wetlands and ecotones across seven sites that collected data in both. Dashed lines connect points from wetland and ecotone within the same site. Neither parameter was significantly different between wetlands and ecotones (paired Wilcoxon, *p* = 1 and 0.67 for richness and abundance, respectively). Bottom: Examples of wildlife in ecotones (gray catbird [ *Dumetella carolinensis* ] at Narragansett Bay NERR RI, domestic cow at Mission‐Aransas NERR TX, black bear at South Slough NERR OR, and gopher snake at Elkhorn Slough NERR CA). Note the disparity in ecotone vegetation types. Data and images are from a continent‐wide study using camera traps in 2022.

There were no significant differences in wildlife species composition (PERMANOVA, *p* = 0.44), richness (paired Wilcoxon, *V* = 8, *p* = 1) or total abundance (paired Wilcoxon, *V* = 11, *p* = 0.67) between wetlands and adjacent wetland‐upland ecotones (Figure 9). Total wildlife abundance exhibited contrasting patterns among sites—it was higher in wetlands compared to ecotones at four sites, but the inverse at three other sites. Although wildlife community composition did not differ between wetlands and ecotones, many upland‐associated species were only detected using ecotones (e.g., yellow warbler [ *Setophaga petechia* ], house finch [ *Haemorhous mexicanus* ], striped skunk [ *Mephitis mephitis* ], California ground squirrel [ *Otospermophilus beecheyi* ], western fence lizard [ *Sceloporus occidentalis* ], and gopher snake [ *Pituophis catenifer* ]).

### 3.5 Diel and Tidal Patterns in Wildlife

**FIGURE 10:**
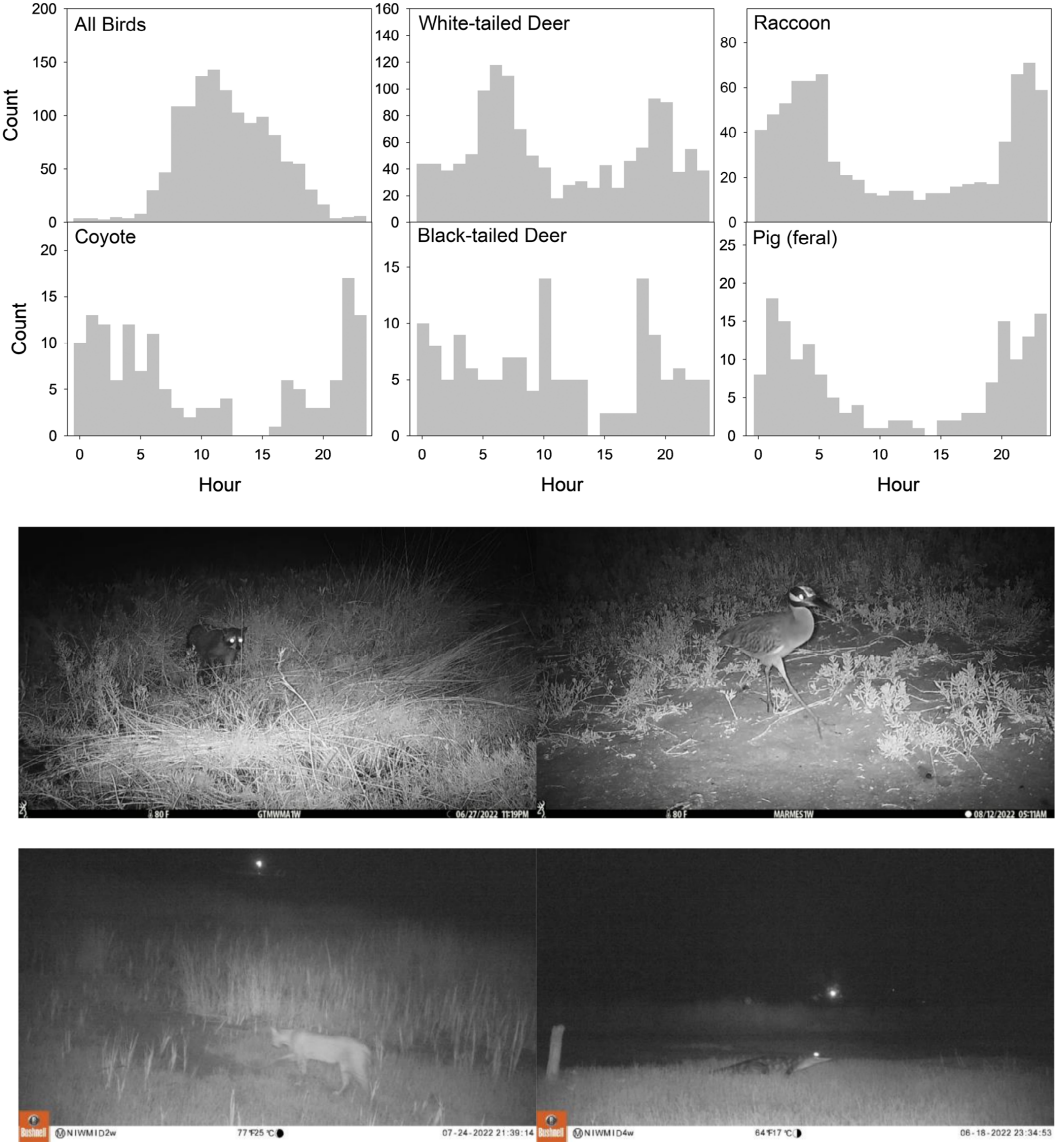
Top: Histograms showing patterns of wildlife in North American coastal wetlands (no ecotone stations) over diel cycles. Except for a few species, birds use coastal wetlands almost exclusively during the day, but mammals tend to be crepuscular or nocturnal, including the five dominant species (white‐tailed deer, raccoon, coyote, black‐tailed deer, and feral pig). Bottom: Examples of wildlife in wetlands at night (from top‐left: Raccoon at GTM NERR FL, black‐crowned night heron [ *Nycticorax nycticorax* ] at Mission‐Aransas NERR TX, bobcat and American alligator [ *Alligator mississippiensis* ] at North‐Inlet Winyah Bay NERR SC). Data and images are from a continent‐wide study using camera traps in 2022.

Wildlife used coastal wetlands over the full diel cycle, but different groups of species used the wetlands at different times of the day; there was a significant difference in wildlife community composition between day, night, and crepuscular periods (PERMANOVA, *p* < 0.001 for main test and each pairwise comparison). Almost all birds (except owls, yellow‐crowned night heron [ *Nyctanassa violacea* ], and a single wild turkey [ *Meleagris gallopavo* ]) used wetlands exclusively during the day, between dawn and dusk (Figure 10). Mammals also used the wetlands during the day, but to a lesser extent, and instead exhibited clear crepuscular or nocturnal activity patterns. Richness was significantly different among the three time periods (Kruskal–Wallis, *χ* ^2^ = 35.3, df = 2, *p* < 0.001) with richness higher during the day compared to night and night in turn higher than crepuscular (Dunn's test, *p* < 0.05). Total counts were also significantly different among the time periods (Kruskal–Wallis, *χ* ^2^ = 25.2, df = 2, *p* < 0.001) with counts higher during the day and at night compared to crepuscular (Dunn's test, *p* < 0.05). Overall, 66% of all wildlife (based on counts) were observed in wetlands during the day, 26% at night, and 8% were crepuscular.

**FIGURE 11:**
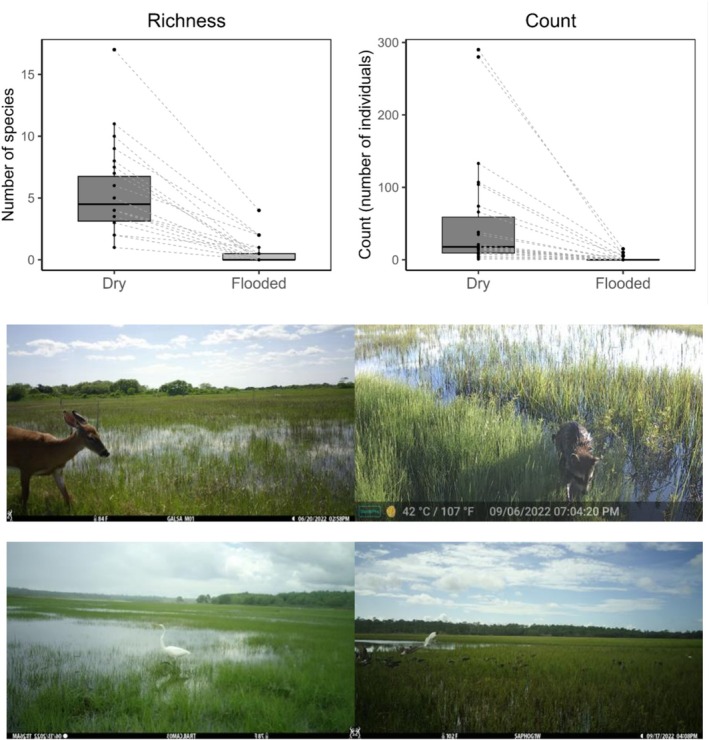
Wildlife use of coastal wetlands varies with tides. Top: Paired box plots for species richness and total wildlife counts in tidal marsh wetlands during periods of dry and flooded conditions across 26 sites. Dashed lines connect points from tidal conditions within the same site. Both parameters were significantly different between dry and flooded (paired Wilcoxon, *p* < 0.001 for both). Bottom: Images of wildlife using flooded wetlands (from top‐left: White‐tailed deer at Galilee Bird Sanctuary RI, raccoon at Sapelo Island NERR GA, great egret at Wells NERR ME, white ibis at GTM NERR FL). Data and images are from a continent‐wide study using camera traps in 2022.

Wildlife also used tidal salt marshes during all tide stages, but counts were significantly higher when marshes were dry vs. flooded (paired Wilcoxon, *V* = 351, *p* < 0.001; Figure 11), as was richness (paired Wilcoxon, *V* = 351, *p* < 0.001). Wildlife communities were also significantly different during the two tide levels (PERMANOVA, *p* = 0.01). At all salt marsh sites, only 324 animals were observed when the wetlands were flooded, compared to 5112 animals when wetlands were not flooded, with just two instances of wildlife using flooded wetlands along the entire west coast (snowy egret in Reserva Natural Punta Mazo, Canada goose in South Slough NERR OR). Overall, the most abundant species at high tides were raccoon (76 individuals), American white ibis ( *Eudocimus albus* ; 63), white‐tailed deer (46), European starling (43), great egret (40), great blue heron (9), and glossy ibis (8) (Figure 11).

## 4 Discussion

### 4.1 Camera Trapping in North America's Coastal Wetlands

We detected 146 species from 61 vertebrate families in vegetated coastal wetlands of North America. While there have been broadscale wildlife survey efforts targeting uplands (Cove et al. 2021; Swanson et al. 2016), as well as extensive surveys of migratory shorebirds in tidal mudflats (Clemens et al. 2014; Mathot et al. 2018), ours is the first extensive field characterization of wildlife in vegetated coastal wetlands. We detected a surprising diversity of species and higher taxa interacting with coastal wetlands, countering the impression that vertebrate diversity in wetlands is low or even “depauperate” (Greenberg 2006). Some taxa—deer, raccoons, coyotes, wading birds—were common in most geographic regions. Nevertheless, wildlife communities had distinctive geographic signatures. Our characterization of wildlife biodiversity was not comprehensive but provides an initial framework for understanding vertebrate communities in coastal wetlands and a baseline for detecting future changes. It illustrates the value of coordinated coastal monitoring networks such as that provided by the NERRS and of collaboration across international borders.

Camera trapping is a powerful non‐invasive approach for revealing the secret life of coastal wetlands, detecting species active by night, and thus not usually encountered by humans visiting them by day. Camera trapping was effective for characterizing the larger vertebrates of wetlands—we detected a range of taxa and body sizes, but did not adequately capture small ground‐dwelling species such as harvest mice, which need different camera deployments (Gracanin et al. 2019; Uhe et al. 2020) or secretive marsh birds which can be detected with playbacks (Conway and Droege 2006). The images of the hidden wildlife in North American wetlands (Appendix S3) are valuable for building public support for conservation and restoration (Herrera et al. 2021; Hsing et al. 2022). We detected wildlife‐wetland interactions that can inform coastal management. Our investigation also illustrated the value of camera trapping for testing hypotheses about temporal and spatial patterns of wildlife use of wetlands, as has been demonstrated for terrestrial ecosystems (Jenks et al. 2011; Kämmerle et al. 2018).

### 4.2 Wildlife‐Wetland Interactions

The abundance and diversity of wildlife we detected in North American wetlands highlight the strong role these species may be playing in shaping the functioning of these ecosystems. Deer were among the most abundant species across all coastal wetlands and are likely having significant effects on marsh vegetation, marsh food webs, and abiotic conditions, similar to what has been observed in terrestrial systems for this species (Bressette et al. 2012). Given their ubiquity, there are surprisingly few studies of deer effects on coastal wetlands (Hannaford et al. 2006; Keusenkothen and Christian 2004). In addition to the common herbivores, we detected two widespread and abundant mesopredators—raccoons and coyotes—which may also be shaping food web dynamics in North American coastal wetlands (Bertness et al. 2014; Gallagher et al. 2019). Ardeids (herons and egrets) were also common and can play strong roles as predators in coastal wetlands (Huang et al. 2015; Raposa et al. 2018). Finally, our study detected a variety of apex predators (e.g., mountain lions, bears) which may exert strong top‐down effects on coastal ecosystems through trophic cascades such as those that have been demonstrated for other species or ecosystems (Atwood and Hammill 2018; Hughes et al. 2013; Nifong and Silliman 2013). Future experimental studies can explore the role these species may play in shaping dynamics of wetland ecosystems. The abundance of predators that we documented in coastal wetlands underlines the need to incorporate top‐down perspectives in coastal wetland restoration (Gaskins et al. 2020; Silliman et al. 2024).

While wildlife may affect the wetlands, their populations may also be affected by them. Our images include clear evidence of coastal wetlands serving as nursery habitat for a variety of mammal and bird species. The role of coastal vegetation as fish nursery habitat is well known (Whitfield 2017), but our results highlight the potential nursery function for other taxa. We also found evidence of the value of coastal wetlands for foraging, with images showing both herbivory and predation. Our many images of animals resting highlight the role wetlands may play as refuges for wildlife species. Wildlife support is often cited as a value of coastal wetlands (Barbier et al. 2011; Peterson et al. 2008), and we provide empirical evidence for this assertion. This demonstration that coastal wetlands provide critical support for wildlife further builds the case for the need for global coastal wetland conservation and restoration (Adams et al. 2021).

### 4.3 Correlates of Wildlife Diversity and Abundance

We detected strong temporal patterns in wildlife use of coastal wetlands. Diel differences in abundance and community composition were distinct. Almost all bird species were observed exclusively during the day, but most mammals were much more abundant at night. Daytime surveys for wildlife would have dramatically underestimated wildlife diversity and abundance. Camera trapping is thus a powerful approach to capturing wildlife species that are active by night (Ikeda et al. 2016; Monterroso et al. 2013). Because most humans visit coastal wetlands by day, they may assume low use by mammals for this ecosystem. Our results can be used to change this perception, both by the public and by managers. We did not camera trap in adjacent uplands to determine whether some of the same species perhaps are more active by day in uplands, which can have higher canopy cover from predators than wetlands. Mammals are known to adjust diel patterns in response to human disturbance levels and cover (Gallo et al. 2022), and may thus show variation between terrestrial and wetland habitat use.

A second temporal pattern revealed by this investigation was the decrease in abundance and diversity of wildlife use of wetlands that are fully inundated. We found that a much more limited suite of animals uses these wetlands when they were flooded. Because acceleration in global sea‐level rise will lead to increased flooding of many existing tidal wetlands (Fagherazzi et al. 2020), they will likely become less valuable to terrestrial wildlife (Krebs et al. 2023), which means the service of wildlife support will decrease in areas without landward migration opportunities due to human infrastructure or other barriers. This highlights the importance of strategies to ensure wildlife continue to have access to at least some wetlands that are only rarely flooded, with climate adaptation strategies such as sediment addition or facilitating tidal marsh migration to higher lands (Wigand et al. 2017).

We also detected clear spatial patterns in wildlife use of coastal wetlands. We found that wetlands with greater landscape heterogeneity (mix of vegetated and unvegetated areas) supported disproportionately higher abundances of raccoons, saltmarsh sparrows, wading birds, and other species. Other studies have also identified the importance of salt marsh heterogeneity for wildlife (McKinney et al. 2009; Smith and Niles 2016). Additionally, we found that wetland‐upland ecotones sometimes have particularly rich wildlife communities. Taken together, these results indicate the value for wildlife of conserving and restoring diverse coastal wetlands, rather than focusing only on the dominant vegetation types.

### 4.4 Biological Invasions

Overall, native species dominated terrestrial vertebrate communities across North American coastal wetlands. Non‐native species were detected at only 12/32 sites. Feral domestic species such as pigs, cows, dogs, and cats are of course ubiquitous and found near all of the sites, but surprisingly they were relatively rare in our observations of continental wetlands of North America, with some exceptions, such as feral pigs that were abundant in various southeast sites. However, these patterns at continental sites are in stark contrast to those at the remote island sites included in this study. Almost all wildlife detected at Heʻeia NERR were non‐native, as were 60% of species in the U.S. Virgin Islands. Islands are well‐known to be highly invaded with devastating impacts to native flora and fauna (Courchamp et al. 2003; Russell et al. 2017), and our results extend this paradigm to their coastal wetlands. Therefore, the conservation and resource management strategies aimed at controlling invasions in terrestrial ecosystems will also benefit coastal wetlands on islands.

### 4.5 Landscape Dynamics: Wildlife at Land‐Sea Interface

Coastal wetlands lie between the land and sea, and as such, they support both terrestrial and aquatic species with overlapping distributions at the land‐sea interface. When the tide is out, terrestrial species use wetlands; when the tide is in, aquatic species move in. Our study detected only a few species known to be dependent on wetlands (rails, marsh sparrows), and some species that are commonly associated with wetlands (mostly birds, such as herons, egrets, geese, gulls, red‐winged blackbirds [ *Agelaius phoeniceus* ]). This is in line with two literature reviews that detected few vertebrates endemic to tidal marshes; most of the vertebrates documented were terrestrial species that occur in coastal wetlands but also in other ecosystems (Canepuccia et al. 2023; Greenberg and Maldonado 2006). Our study reveals how heavily terrestrial species use coastal wetlands. Local managers we worked with expressed surprise at the frequency of detections of species they had considered strictly terrestrial—species such as deer, turkey, mountain lions, and elk ( *Cervus elaphus canadensis* ). Consideration of coastal wetland use by wildlife species should inform management strategies in coastal areas. In some cases, coastal wetlands may support larger population sizes of wildlife species in an area than terrestrial ecosystems alone would provide. For example, small mammals may benefit from foraging seasonally in marsh when adjacent grassland is brown and dry, and vice versa (Canepuccia et al. 2010; Harding 2002). Small mammal abundance in the Southwest Atlantic was found to be related to marsh characteristics, but richness was related to upland features, highlighting the importance of considering adjacent wetlands and uplands (Canepuccia et al. 2015).

Some of the terrestrial species we detected were very common in coastal wetlands and could have important effects spanning upland‐wetland ecosystems. For example, raccoons were a commonly detected species, and their effects on wetlands could be intensified by conditions in adjacent uplands, or vice versa. Areas where adjacent uplands have appropriate daytime denning habitat for raccoons, their effects on aquatic populations such as clams or crabs could be stronger than in areas without such upland cover. Conversely, in areas where wetland food sources subsidize raccoon populations, their impacts on terrestrial ecosystems, such as by consuming songbird eggs and nestlings, could be magnified. Predation by terrestrial mammals is widespread globally in the intertidal zone, though rarely studied, and can result in transfer of energy from marine to upland ecosystems (Carlton and Hodder 2003). Marine–terrestrial linkages have been shown to play an important role in many systems (Harding and Stevens 2001; Helfield and Naiman 2006; Polis and Hurd 1996). Coastal wetlands, sandwiched between marine and terrestrial environments, may play an especially important role in such linkages. An earlier study found that tidal marsh restoration success was impacted by terrestrial herbivores, which could be controlled by management of weedy cover in adjacent uplands (Wasson et al. 2021). Likewise, marsh disturbance by feral pigs was related to the extent of adjacent hardwood forest (Sharp and Angelini 2019). Characterizing the role of coastal wetlands in marine–terrestrial linkages driven by wildlife is thus a rich area for future study and highlights the importance of a landscape approach to conservation and resource management planning, integrating multiple adjacent ecosystems and the wildlife species they support.

## Author Contributions

**Kenneth B. Raposa:** conceptualization (lead), data curation (lead), formal analysis (supporting), funding acquisition (lead), investigation (equal), methodology (equal), project administration (lead), supervision (lead), writing – original draft (equal), writing – review and editing (lead). **Kimberly Cressman:** formal analysis (lead), investigation (supporting), visualization (equal), writing – review and editing (supporting). **Danika vanProosdij:** investigation (supporting), writing – review and editing (supporting). **Jason Goldstein:** investigation (supporting), writing – review and editing (supporting). **Rachel A. Stevens:** investigation (supporting), writing – review and editing (supporting). **Megan Tyrrell:** investigation (supporting), writing – review and editing (supporting). **Brian DeGasperis:** investigation (supporting), writing – review and editing (supporting). **Kari St. Laurent:** investigation (supporting), writing – review and editing (supporting). **R. Kyle Derby:** investigation (supporting), writing – review and editing (supporting). **Scott Lerberg:** investigation (supporting), writing – review and editing (supporting). **Elizabeth Fox Pinnix:** investigation (supporting), writing – review and editing (supporting). **Jennifer Plunkett:** investigation (supporting), writing – review and editing (supporting). **Jessica Kinsella:** investigation (supporting), writing – review and editing (supporting). **Colby Peffer:** investigation (supporting), writing – review and editing (supporting). **Candace Killian:** investigation (supporting), writing – review and editing (supporting). **Jay Black:** investigation (supporting), writing – review and editing (supporting). **Katie Swanson:** investigation (supporting), writing – review and editing (supporting). **Christopher Biggs:** investigation (supporting), writing – review and editing (supporting). **Emily Kuzmick:** investigation (supporting), writing – review and editing (supporting). **Angel Dieppa‐Ayala:** investigation (supporting), writing – review and editing (supporting). **Kristin Wilson Grimes:** investigation (supporting), writing – review and editing (supporting). **Allie Durdall:** investigation (supporting), writing – review and editing (supporting). **Jacob Argueta:** investigation (supporting), writing – review and editing (supporting). **Thomas Reid:** investigation (supporting), writing – review and editing (supporting). **Roger Fuller:** investigation (supporting), writing – review and editing (supporting). **Jennifer Schmitt:** investigation (supporting), writing – review and editing (supporting). **Matthew C. Ferner:** investigation (supporting), writing – review and editing (supporting). **Mônica Almeida:** investigation (supporting), writing – review and editing (supporting). **Héctor Manuel Sánchez Márquez:** investigation (supporting), writing – review and editing (supporting). **Yoshimi M. Rii:** investigation (supporting), writing – review and editing (supporting). **A. Nālani Olguin:** investigation (supporting), writing – review and editing (supporting). **Maureen Dewire:** investigation (supporting), writing – review and editing (supporting). **Kerstin Wasson:** conceptualization (supporting), formal analysis (supporting), funding acquisition (supporting), investigation (equal), methodology (equal), project administration (supporting), supervision (supporting), writing – original draft (equal), writing – review and editing (supporting).

## Funding

This work was supported by the National Science Foundation (1930991, 1946412); National Oceanic and Atmospheric Administration, Office for Coastal Management (NA19NOS4190058).

## Conflicts of Interest

The authors declare no conflicts of interest.

## Supporting information


**Appendix S1:** ece372872‐sup‐0001‐AppendixS1.xlsx.

**Appendix S2:** ece372872‐sup‐0002‐AppendixS2.docx.

**Appendix S3:** ece372872‐sup‐0003‐AppendixS3.pdf.

## Data Availability

All the data used in the study are included and available in Appendix S1.
