# Supplementary material for: The Secret Life of Tidal Marshes and Mangroves: Camera Trapping as a Window Into Wildlife Using North American Coastal Wetlands
Source: Ecol Evol. 2026 Jan 15;16(1):e72872. doi: 10.1002/ece3.72872 (PMC12805223; doi:10.1002/ece3.72872)
Supplement: Supplementary file 2 — Appendix S2: ece372872‐sup‐0002‐AppendixS2.docx. [file ECE3-16-e72872-s002.docx]

# Appendix S2. Detailed camera trapping methods, other tables and figures, and additional site-specific funding acknowledgements.

**Detailed methods for camera trapping and image processing**

**Camera trapping**

Our project largely relied on pre-existing camera traps already available at each site, but to enhance sample size at sites with just one or two cameras, and to improve consistency among sites, the Narragansett Bay NERR loaned one or more cameras (Browning Strike Force HD Pro X model) to sites that needed them. In total, we used Browning cameras at 74 of 109 stations, Bushnell at 21, and other camera makes/models at the remaining 14 stations (**Table 1**). Most camera-trap models used in our study have very similar capture efficiencies (<https://www.trailcampro.com/pages/trail-camera-shootout>). Mixed-camera model surveys such as ours can be very effective, especially in large studies focused on examining general patterns in wildlife among major ecosystems or across broad spatial scales (Hedwig et al., 2018; Meyer et al., 2015).

Camera programming varied somewhat by make and model, but we used similar settings to improve data comparability. This included using a 5-minute delay between consecutive images to minimize numerous images of the same individual, medium sensitivity to balance between collecting very high numbers of false triggers (images with no animals) and missing animals passing within the viewfield, and medium flash power for improved images at night. Cameras were always deployed with date and timestamps to track wildlife use of wetlands over time. Example settings used for Browning Strike Force HD Pro X cameras are shown in **Table S2.1**; sites used similar settings when using any other camera.

In the field, cameras could be deployed on any stable structure, but most were deployed on wooden stakes sunk into the wetland soil. Cameras were mounted to face slightly down (~3-5 degrees) to help minimize glare. They were deployed facing the water (looking seaward across the coastal vegetation below them) and could be deployed in any ordinal direction. Some cameras were mounted in lockboxes if vandalism or theft was a concern. At some stations potentially prone to false triggers due to wind-blown vegetation, a small amount of vegetation was cut or removed in front of the camera, without altering overall vegetation composition at the station. Immediately after initial deployment, two pictures were taken of each camera trap (one ~20 m away, facing straight towards the camera face, and another from the side; **Fig. S2.1**). All deployment data (e.g., date, time, height, coordinates, etc.) were recorded on standardized field sheets.

Camera traps were checked periodically (target = every 2-3 weeks) during deployment to check and replace batteries as needed, swap or download collected images from SD cards, and conduct routine maintenance as needed.

**Image processing and management**

All collected images were stored and backed up locally at each site and then transferred to the Narragansett Bay NERR, where they were also stored on local computers and backed up on RI Department of Environmental Management servers. All pictures of the camera trap stations at deployment and all field sheets were also sent to the Narragansett Bay NERR.

When Narragansett Bay NERR staff could not identify wildlife on an image, staff and other wildlife experts from the individual sites were consulted. Individual animals were identified to species when possible, otherwise to the lowest possible taxonomic level. Individual animals were only counted if they were considered to be in the camera view frame, indicating they had directly triggered the camera. Wildlife outside the view frame (i.e., bycatch, in the distance but captured on an image triggered by moving vegetation) were also identified and counted but not included in this study. When camera traps were initially deployed at the Narragansett Bay NERR, PVC stakes were installed at distances to match most camera trap detection ranges used in this study to help staff doing image interpretation differentiate between animals within and beyond the view field.

# References

Hedwig, D., Kienast, I., Bonnet, M., Curran, B.K., Courage, A., Boesch, C., Kühl, H.S., & King T. (2018). A

camera trap assessment of the forest mammal community within the transitional savannah-forest mosaic of the Batéké Plateau National Park, Gabon. *African Journal of Ecology*, *56*, 777-790. https://doi.org/10.1111/aje.12497

Meyer, N.F.V., Esser, H.J., Moreno, R., van Langevelde, F., Liefting, Y., Oller, D.R., Vogels, C.B.F., Carver,

A.D., Nielsen, C.K., & Jansen, P.A. (2015). An assessment of the terrestrial mammal communities

in forests of Central Panama, using camera-trap surveys. *Journal for Nature Conservation*, *26*,

28-35. https://doi.org/10.1016/j.jnc.2015.04.003

**Table S2.1.** Programming settings for the Browning Strike Force HD Pro X cameras used in this study. Similar settings were used for other make/model camera traps.


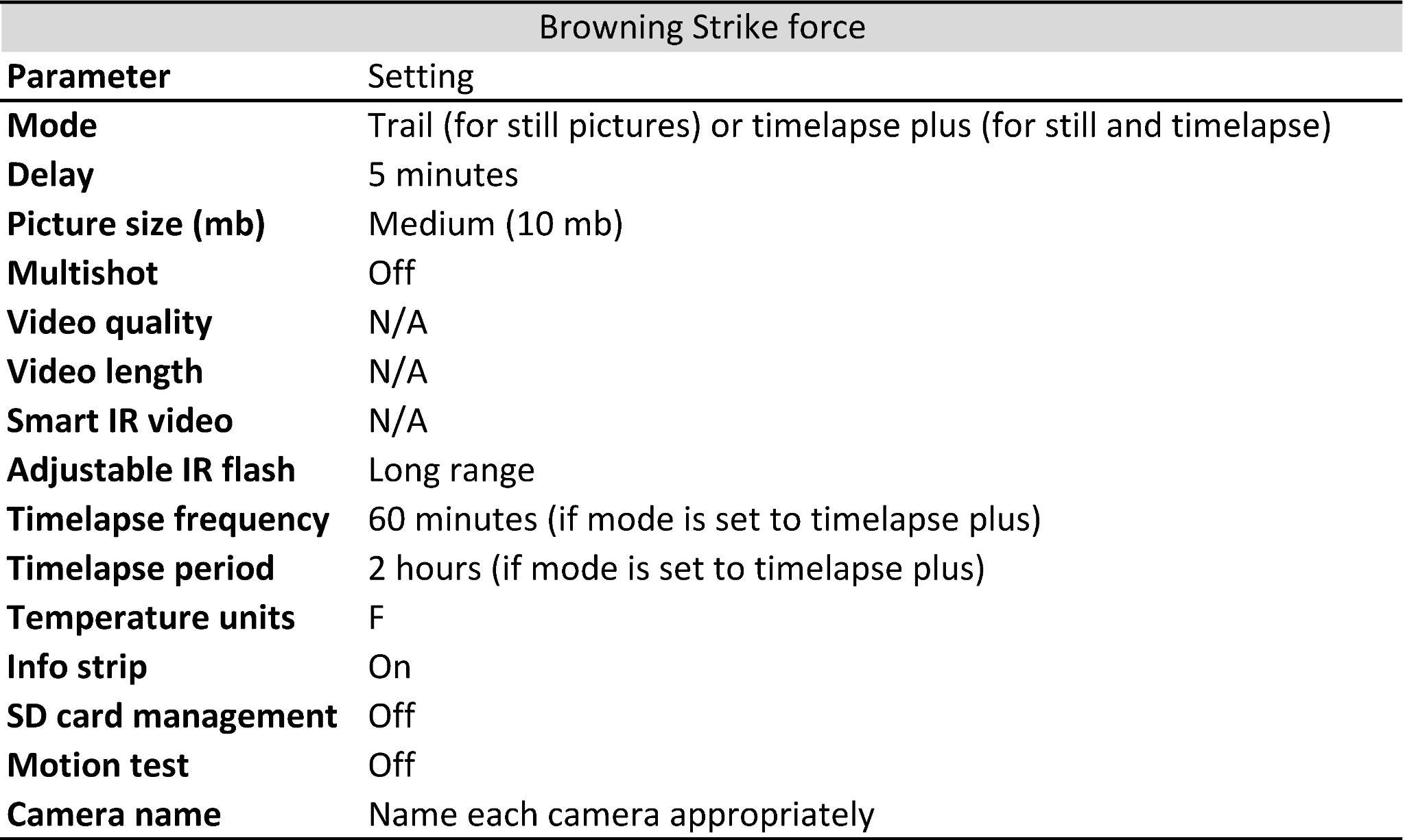


**Table S2.2**. Descriptions of main analyses used in this study. Data from ecotone stations were only included in some biodiversity analyses and the wetland vs ecotone comparisons; all other analyses used data only from wetland stations. All data were collected during summer 2022.


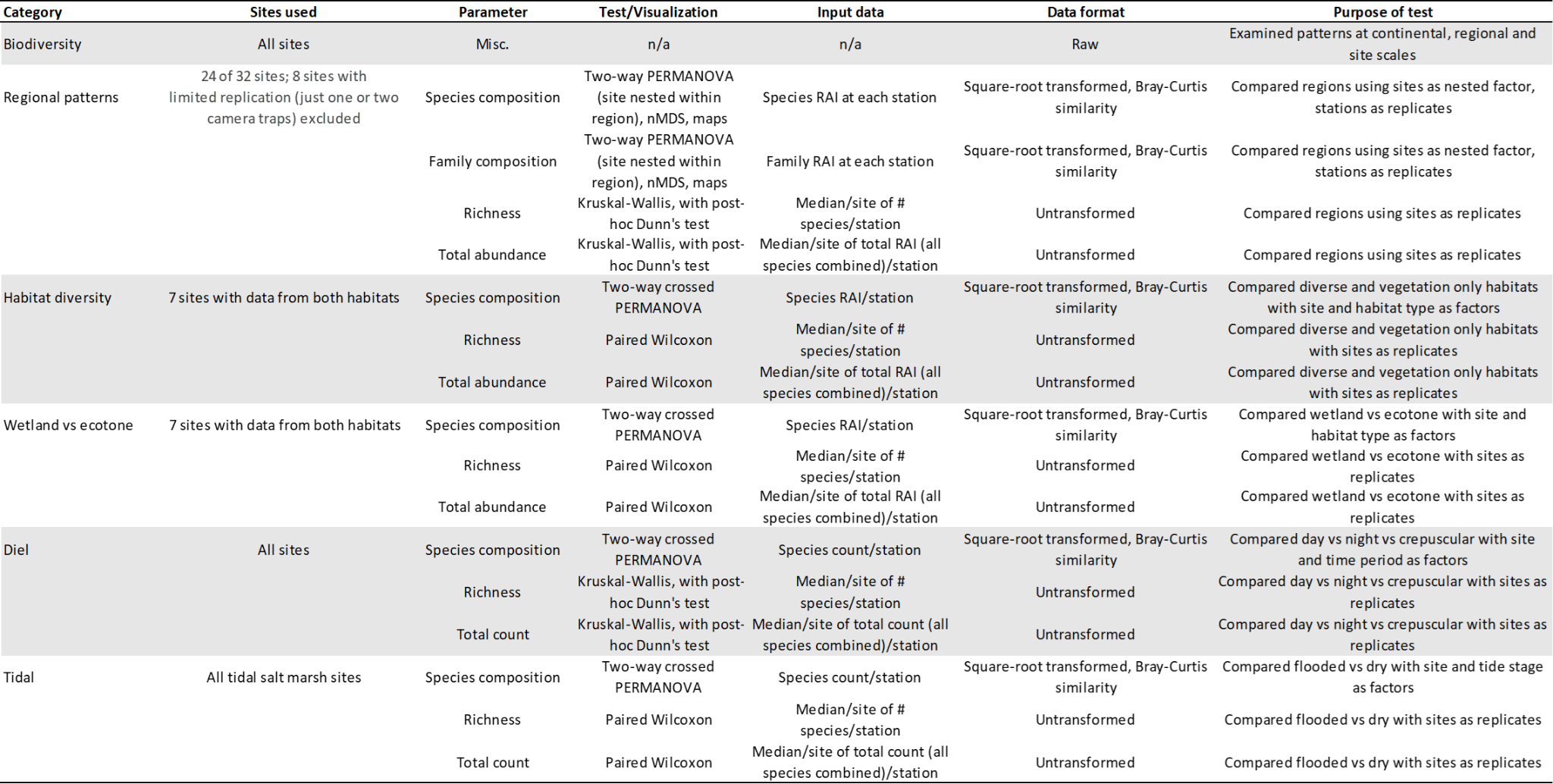


**Figure S2.1**. Examples of camera traps deployed in the field. Top left: two camera traps deployed on the same stake at the Narragansett Bay NERR RI, one facing into the wetland and the other facing in the opposite direction into the ecotone. Top right: one wetland and one ecotone camera mounted on separate stakes at the Elkhorn Slough NERR CA. Bottom left and right: wetland camera traps deployed at the Hudson River NERR NY and Chesapeake Bay VA NERRs, respectively.


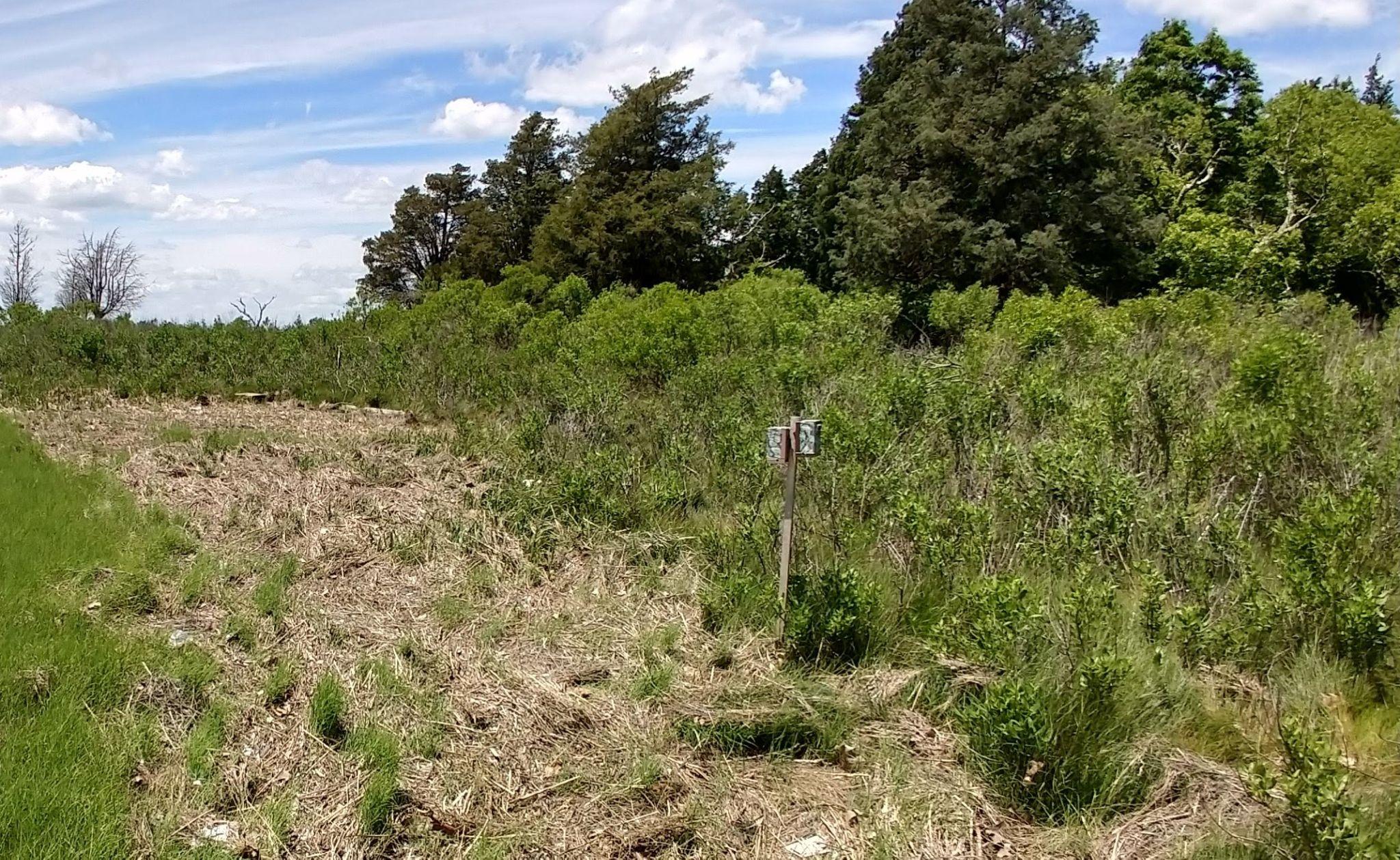

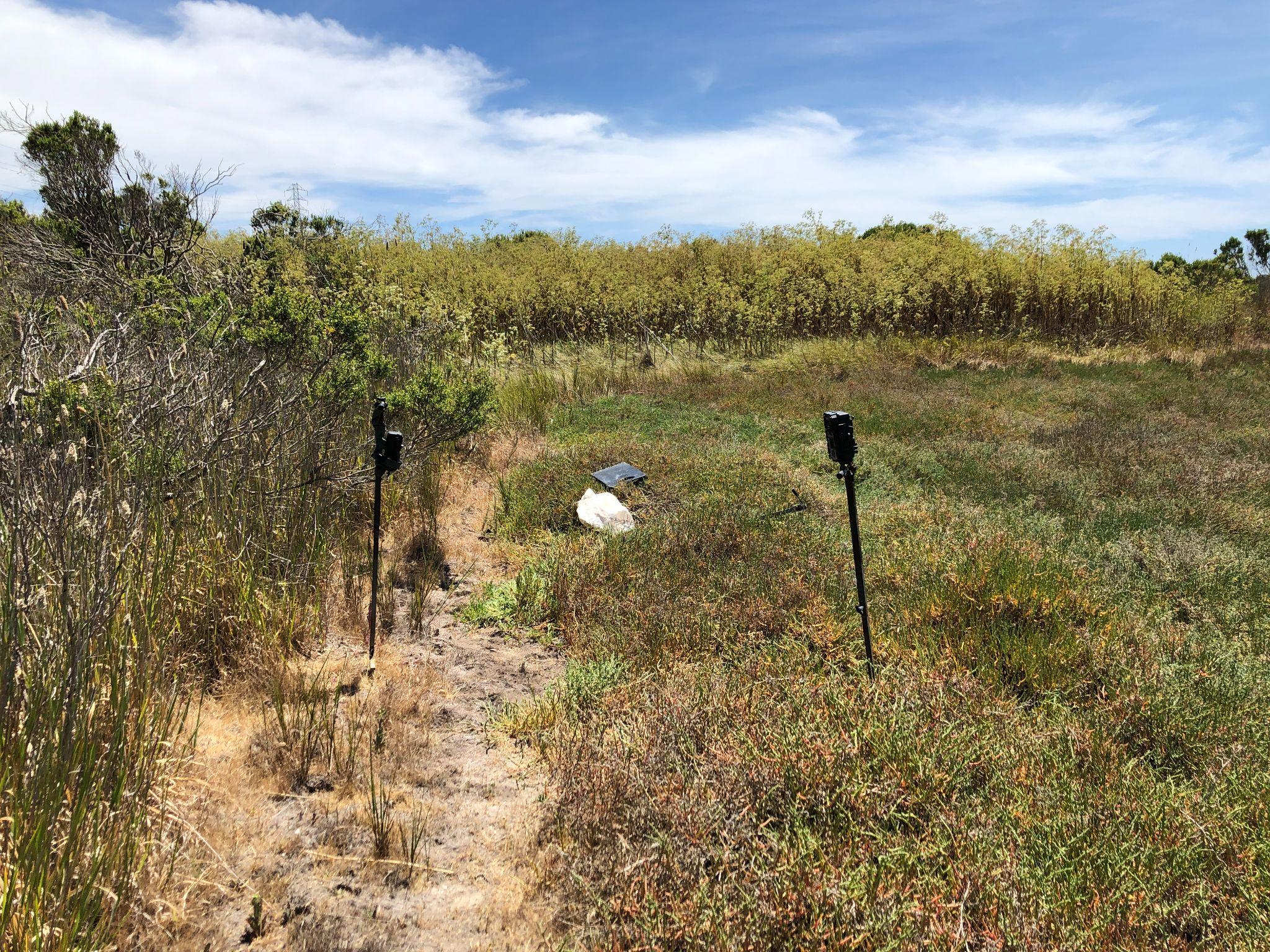


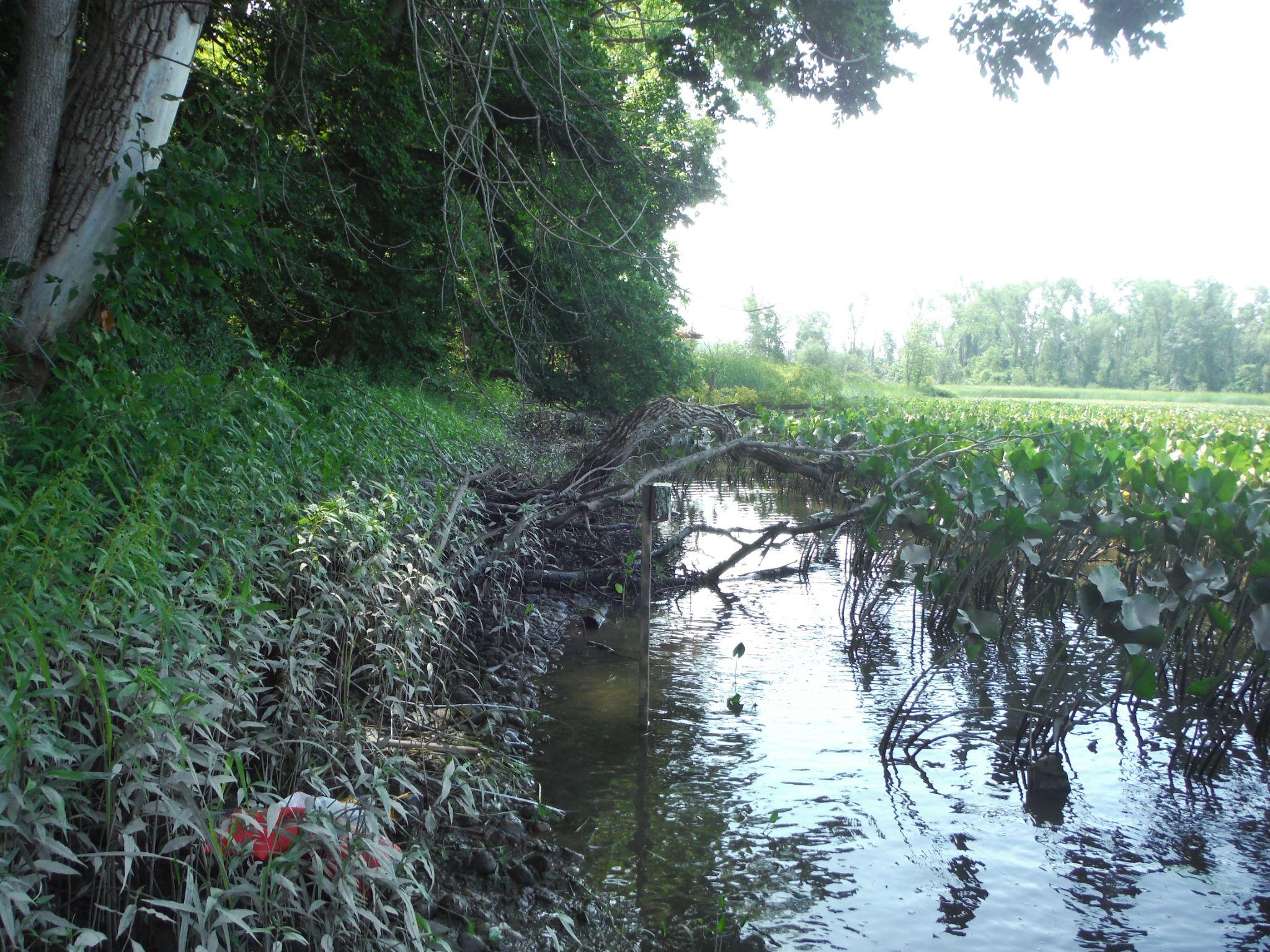

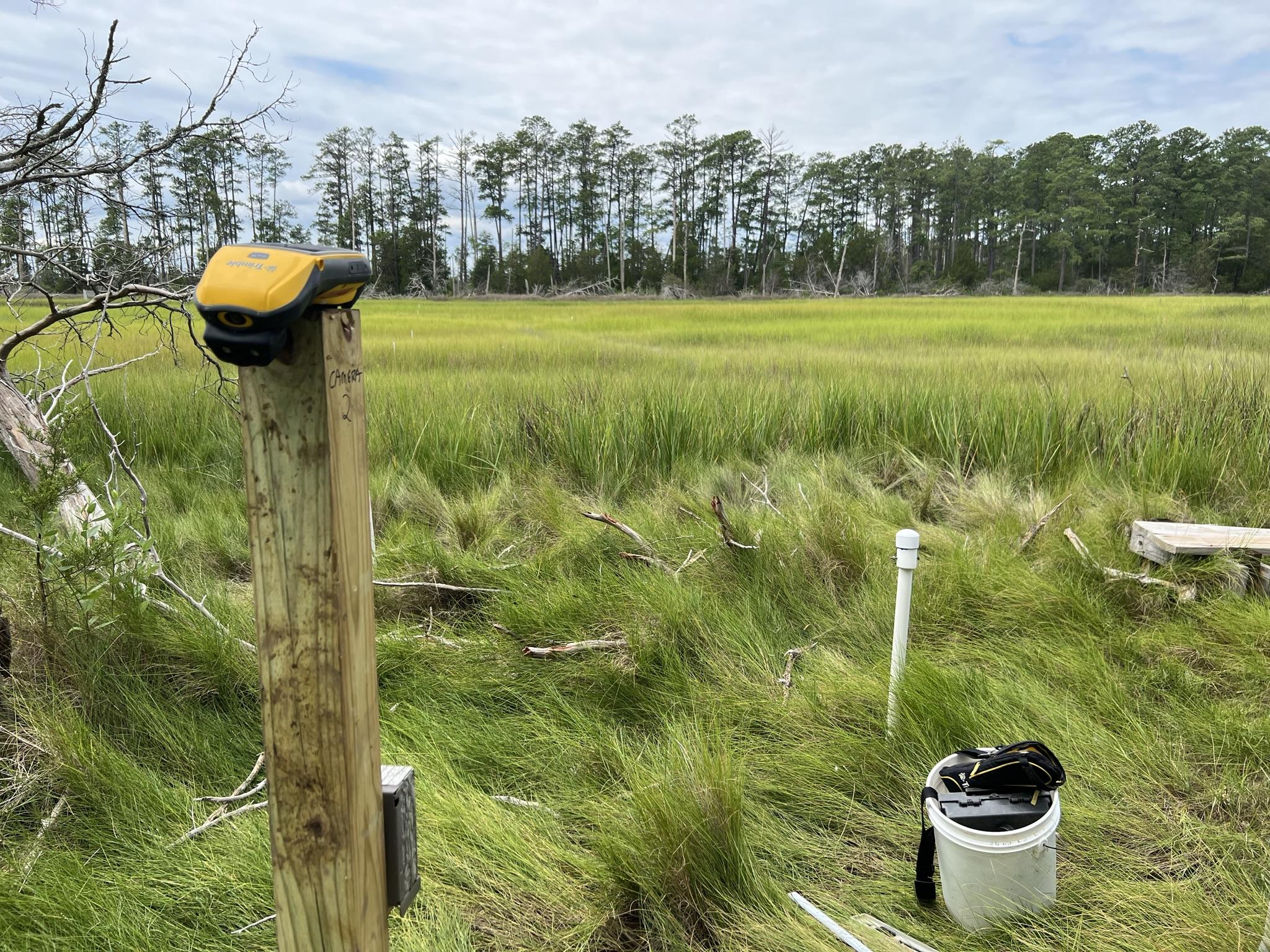


**Figure S2.2**. Paired box plots for species richness and total abundance (as RAI) in heterogeneous (vegetation with water or bare) and homogeneous (vegetation only) landscapes across seven sites that collected data in both. Dashed lines connect points from different landscapes within the same site. Neither parameter was significantly different between landscape types (paired Wilcoxon, *p* = 0.27 and 0.45 for richness and abundance, respectively).


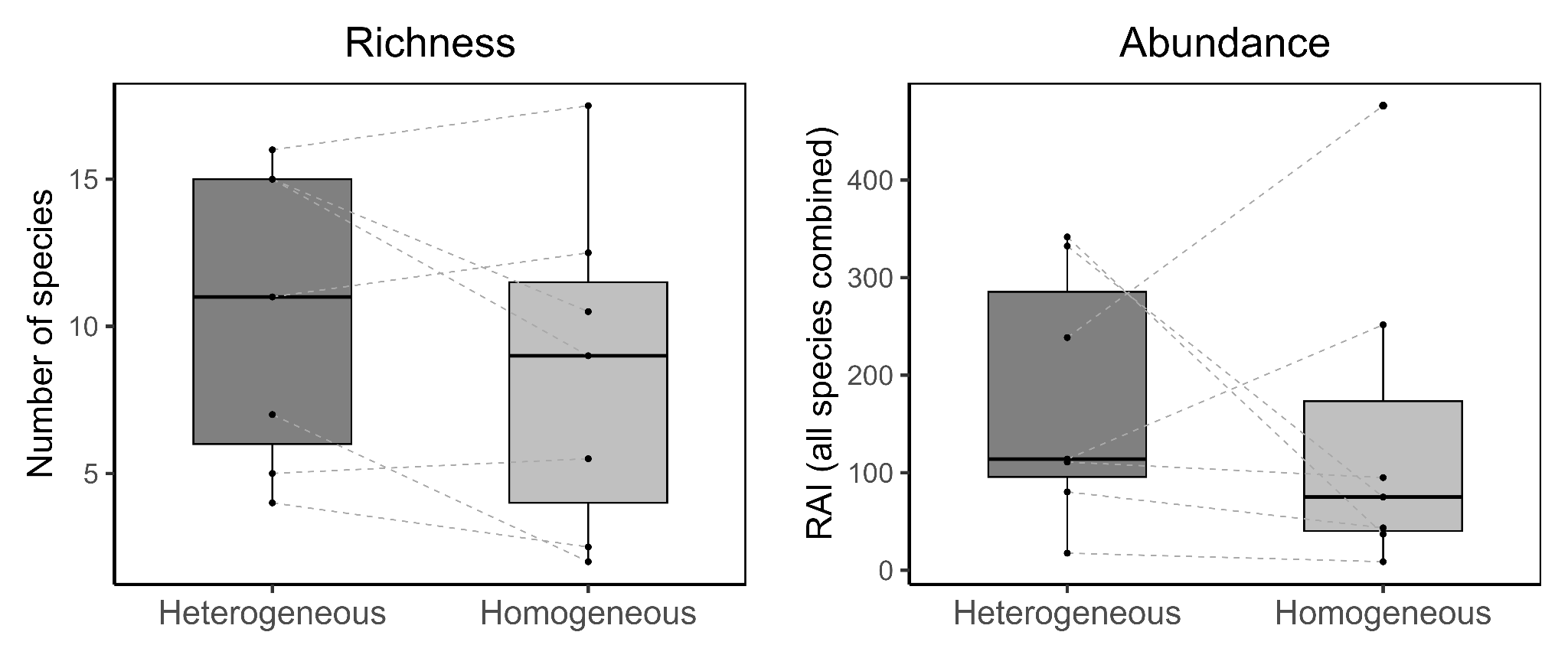


#

# Additional funding acknowledgements

# In the U.S. Virgin Islands, this work was conducted under permit DFW20059U. For KWG and AD, this material is based upon work supported by the U.S. National Science Foundation under award numbers (Awards 1930991, 1946412). Any opinions, findings, and conclusions or recommendations expressed in this material are those of the authors and do not necessarily reflect the views of funders. This manuscript is submitted for publication with the understanding that the United States Government is authorized to reproduce and distribute reprints for Governmental purposes. This is contribution 304 from the University of the Virgin Islands Center for Marine and Environmental Studies.
