# Supplementary material for: The Secret Life of Tidal Marshes and Mangroves: Camera Trapping as a Window Into Wildlife Using North American Coastal Wetlands
Source: Ecol Evol. 2026 Jan 15;16(1):e72872. doi: 10.1002/ece3.72872 (PMC12805223; doi:10.1002/ece3.72872)
Supplement: Supplementary file 3 — Appendix S3: ece372872‐sup‐0003‐AppendixS3.pdf. [file ECE3-16-e72872-s001.pdf]

Appendix S3. Additional photographs of wildlife in North American coastal wetlands, including examples of apex and mesopredators, raptors, non-native species, and nursery function. For even more images across the sites, see: https://www.flickr.com/photos/165674165@N07/albums/72177720309270386/

Apex predators in coastal wetlands

Note: these are example images from this study; they are not inclusive of all apex predators found in coastal wetlands.

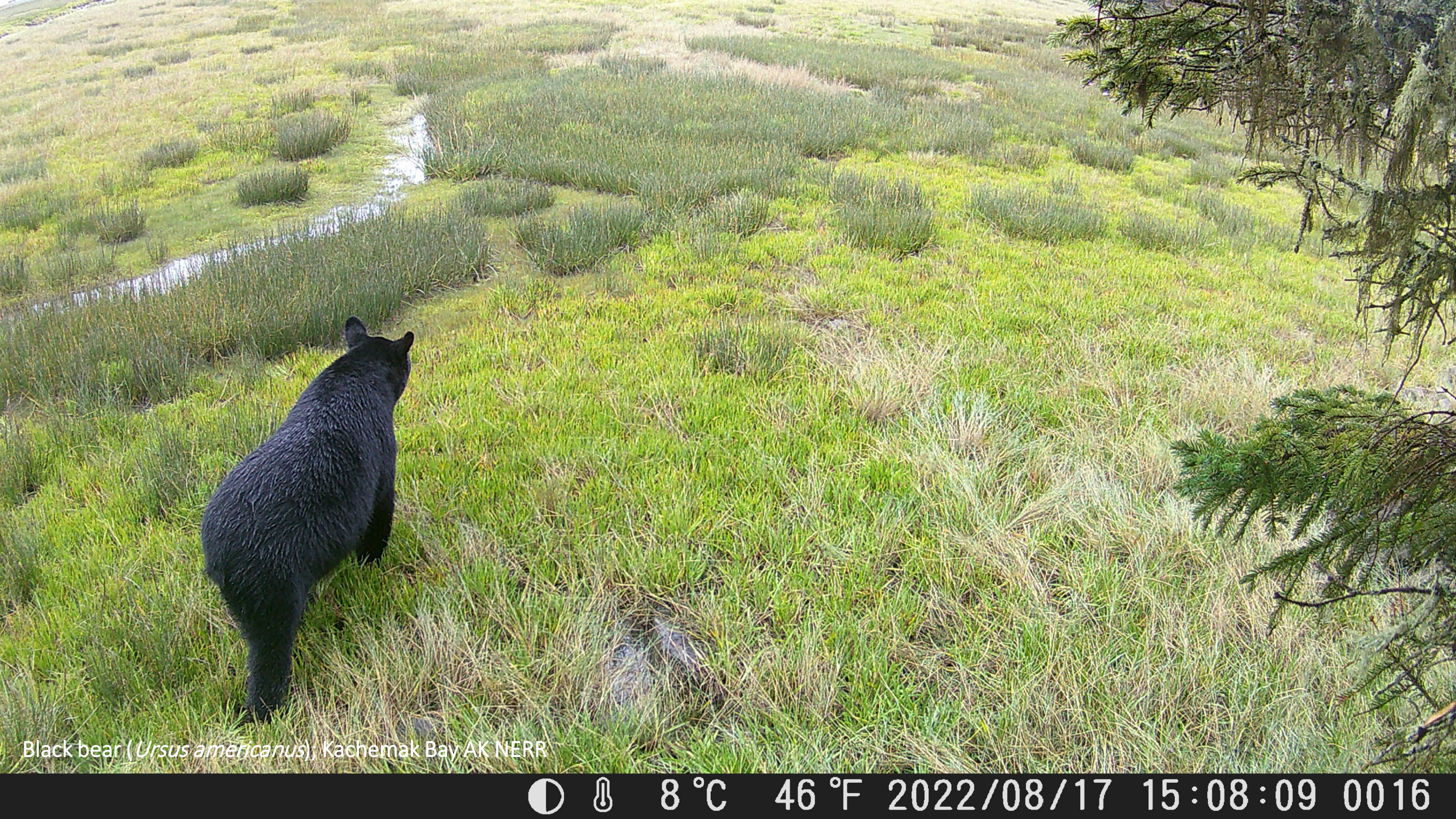

Black bear (*Ursus americanus*); Kachemak Bay AK NERR

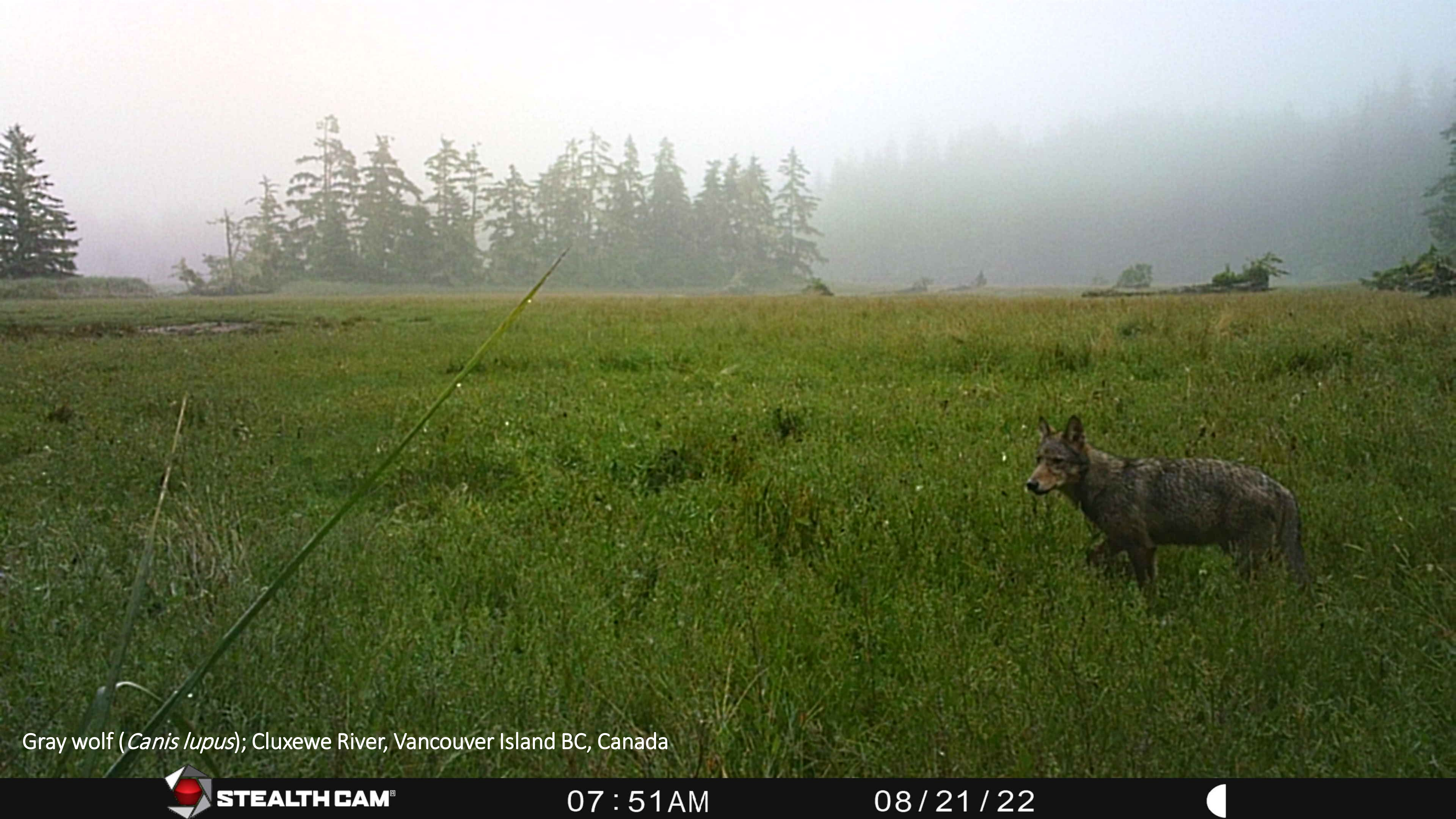

Gray wolf (*Canis lupus*); Cluxewe River, Vancouver Island BC, Canada

Mountain lion (*Puma concolor*); Salmon River, Vancouver Island BC, Canada

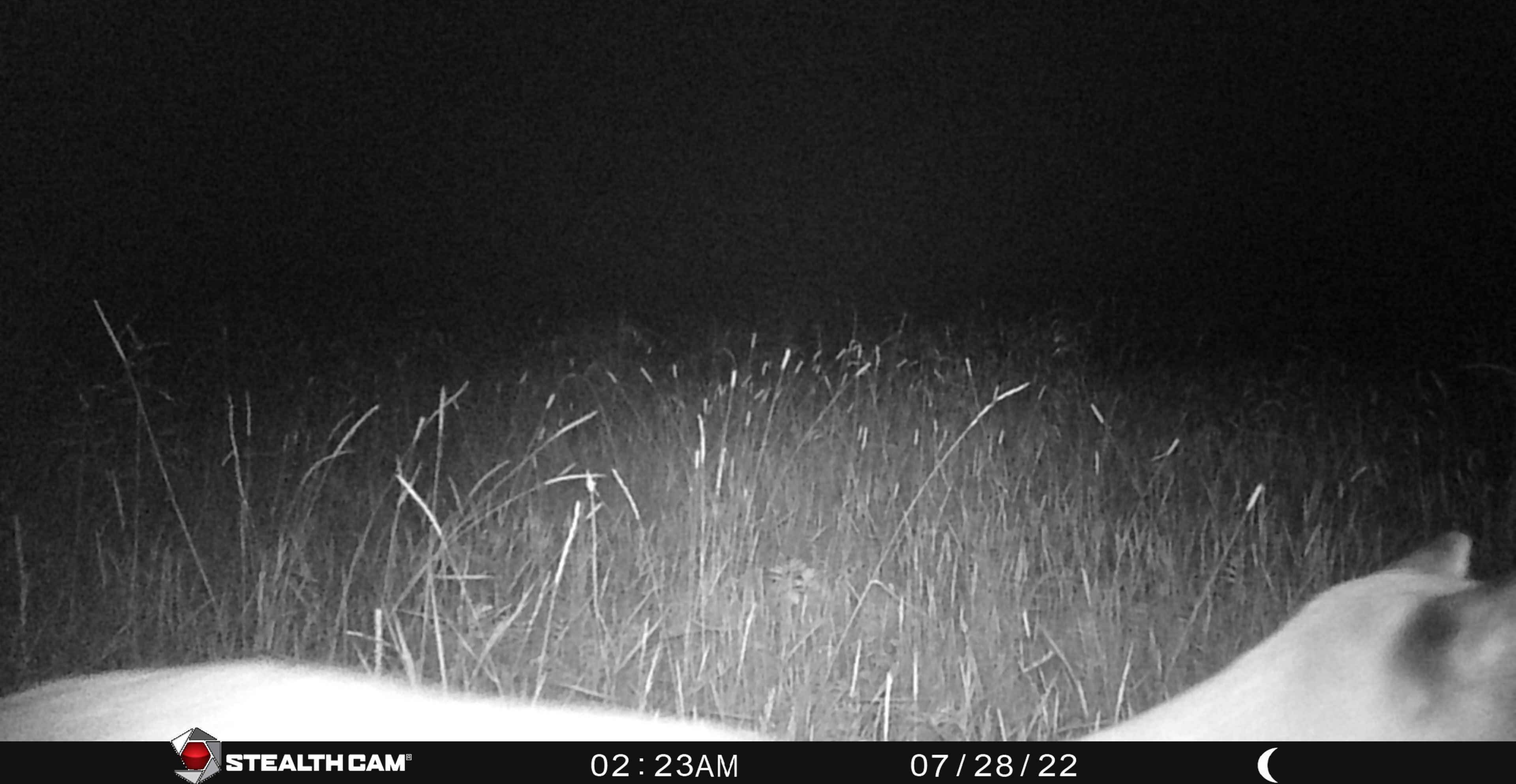

Mesopredators in coastal wetlands

Note: these are example images from this study; they are not inclusive of all mesopredators found in coastal wetlands.

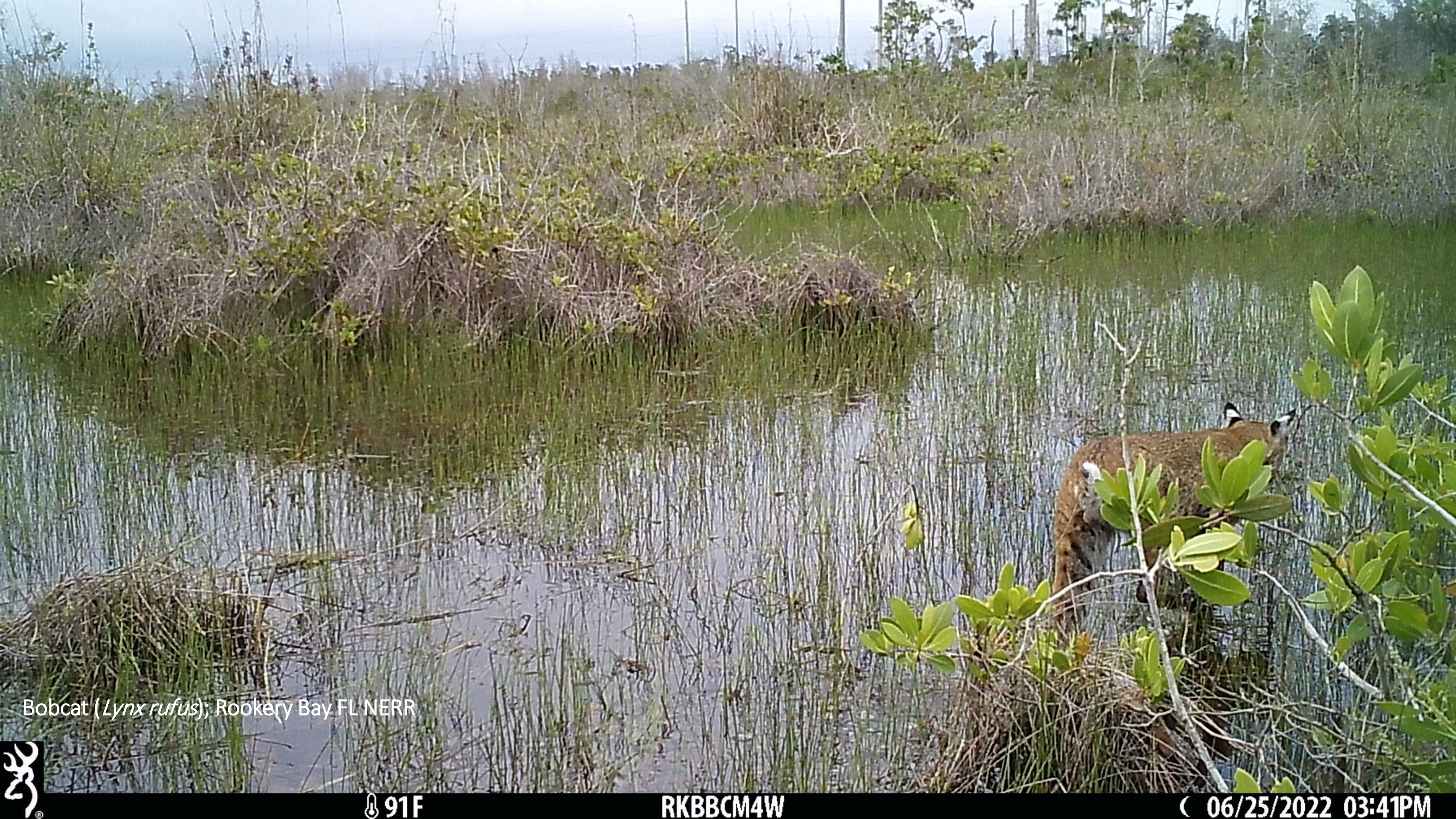

Bobcat (*Lynx rufus*); Rookery Bay FL NERR

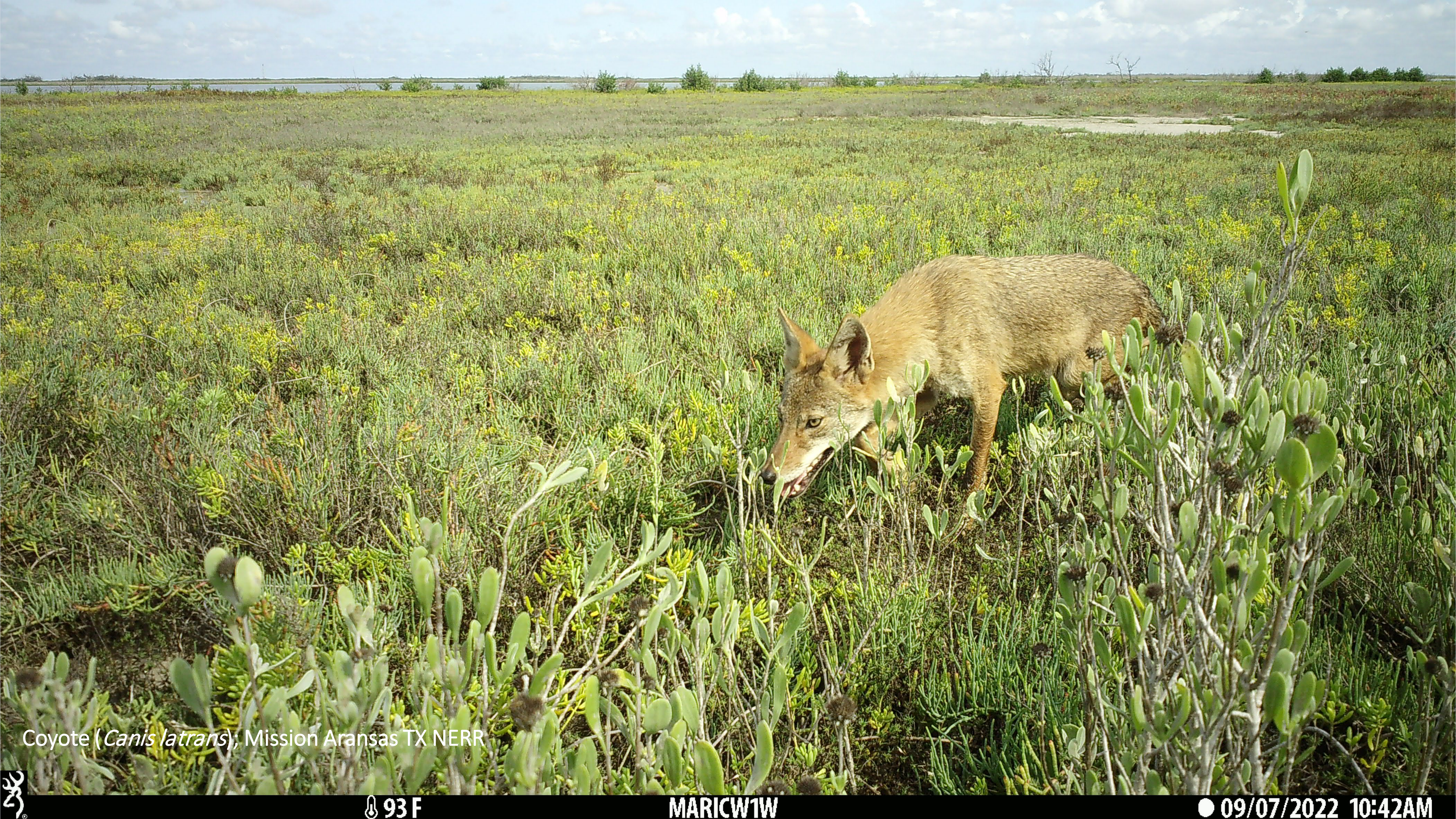

Coyote (*Canis latrans*), Mission Aransas TX NERR

Nine-banded armadillo (*Dasypus novemcinctus*); GTM FL NERR

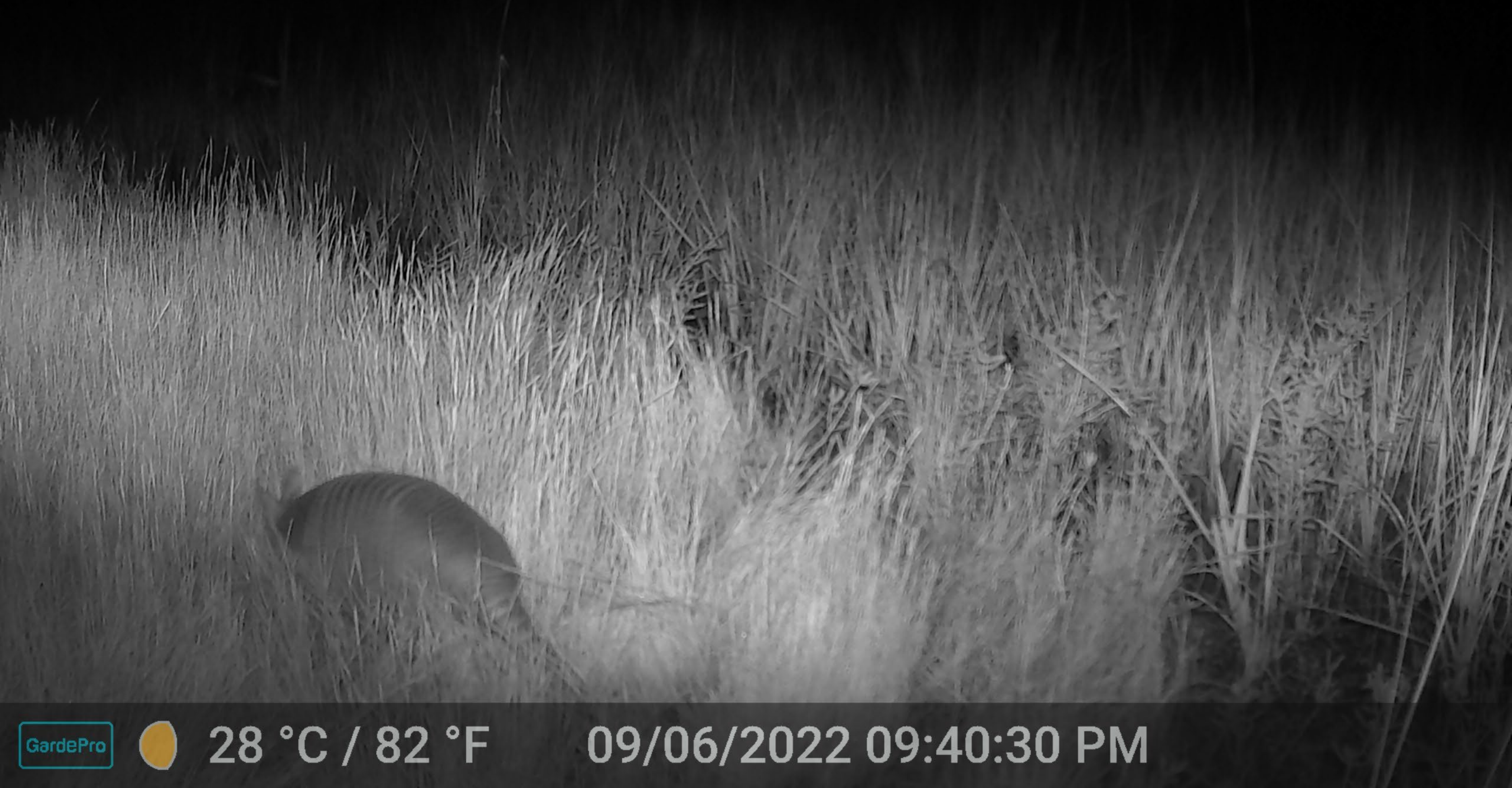

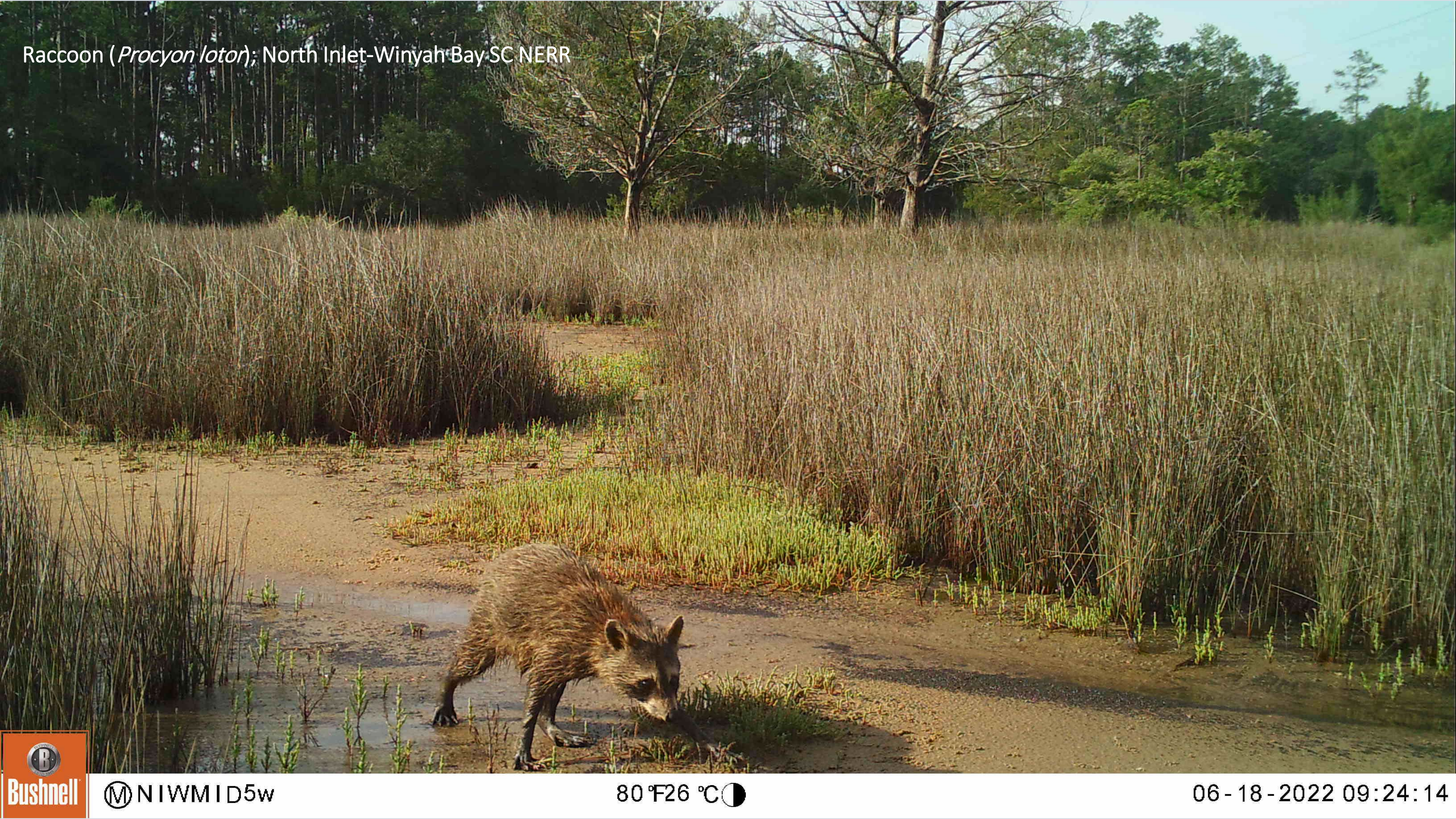

Raccoon (*Procyon lotor*); North Inlet-Winyah Bay SC NERR

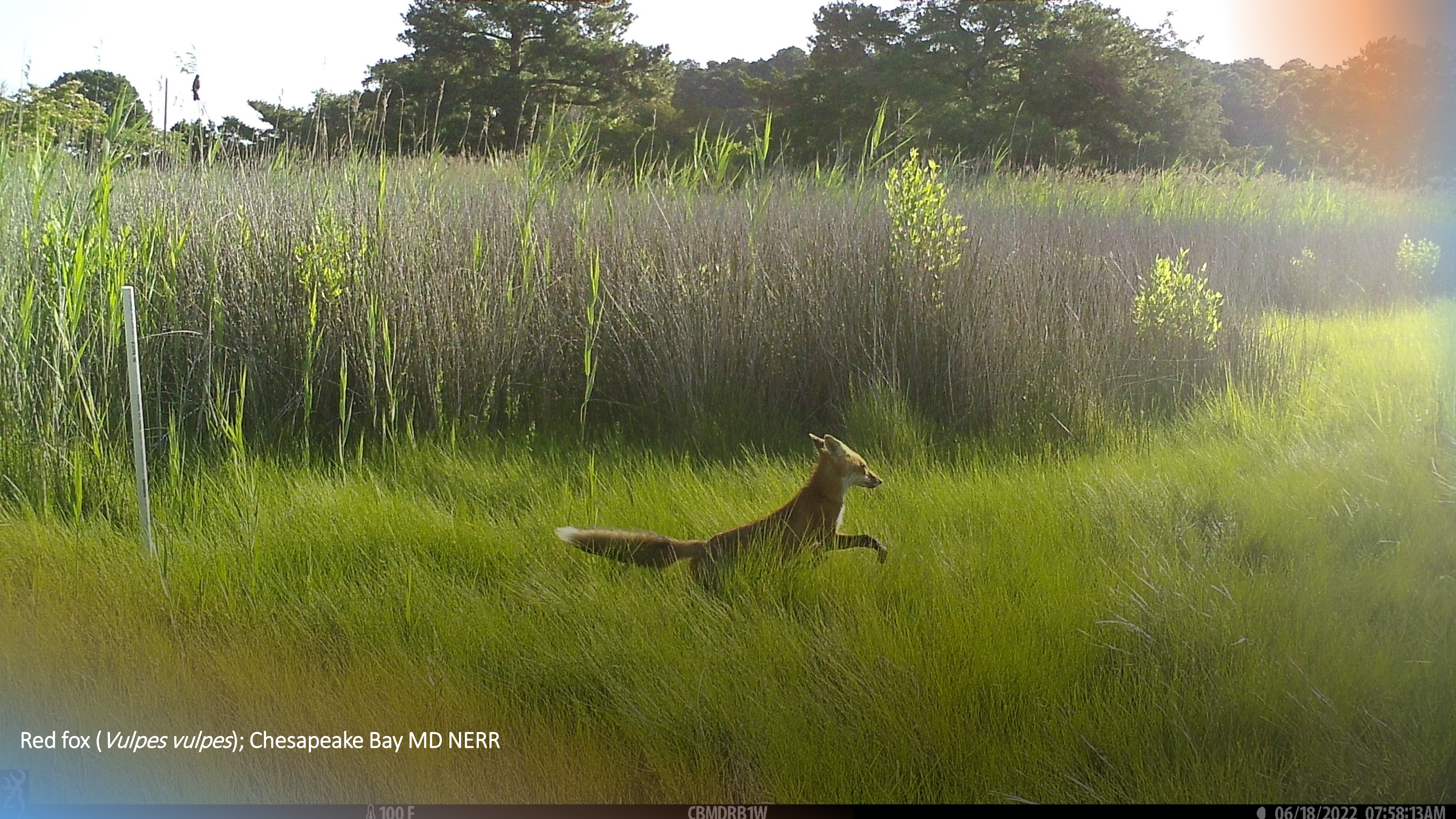

Red fox (*Vulpes vulpes*); Chesapeake Bay MD NERR

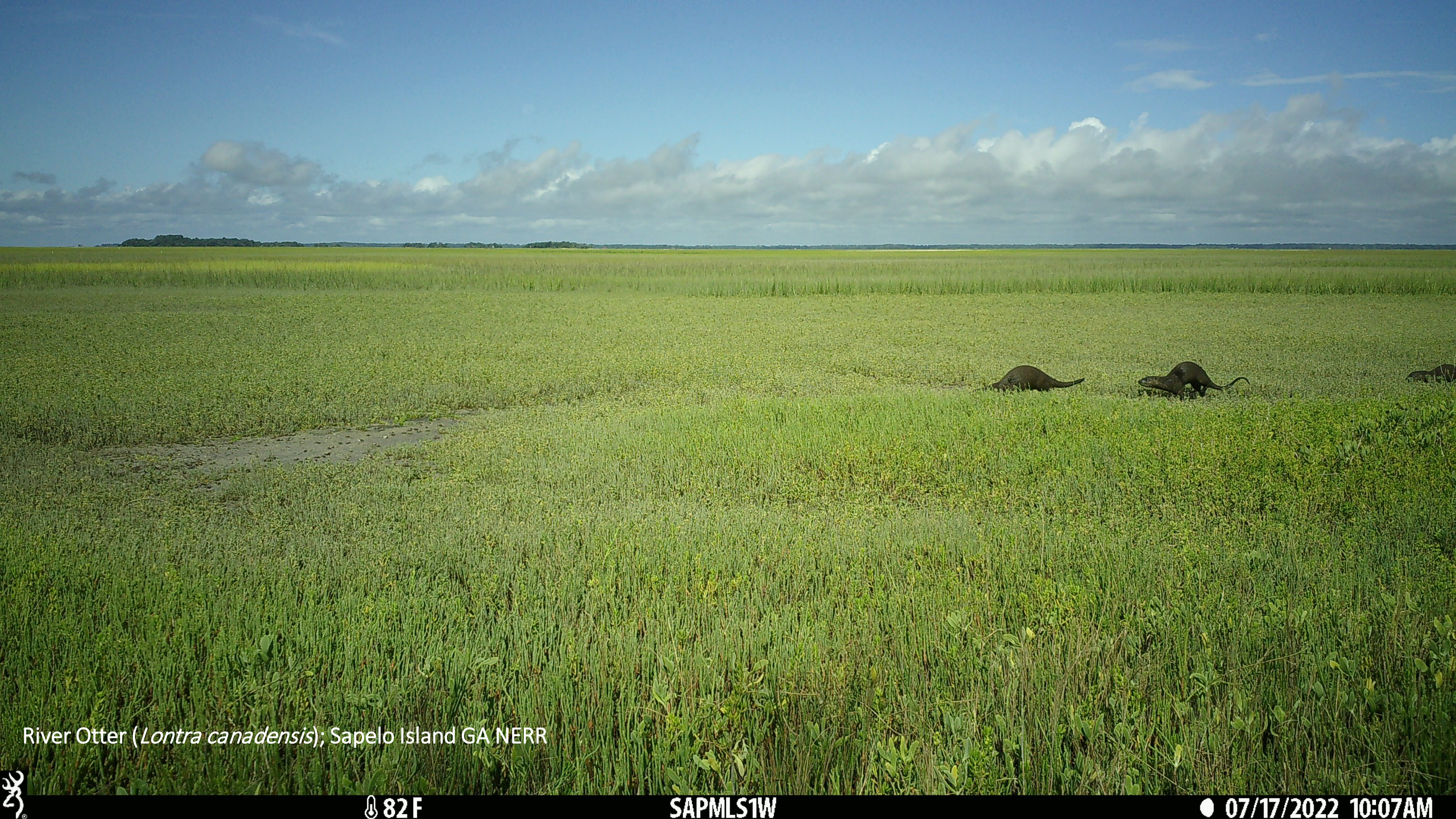

River Otter (*Lontra canadensis*); Sapelo Island GA NERR

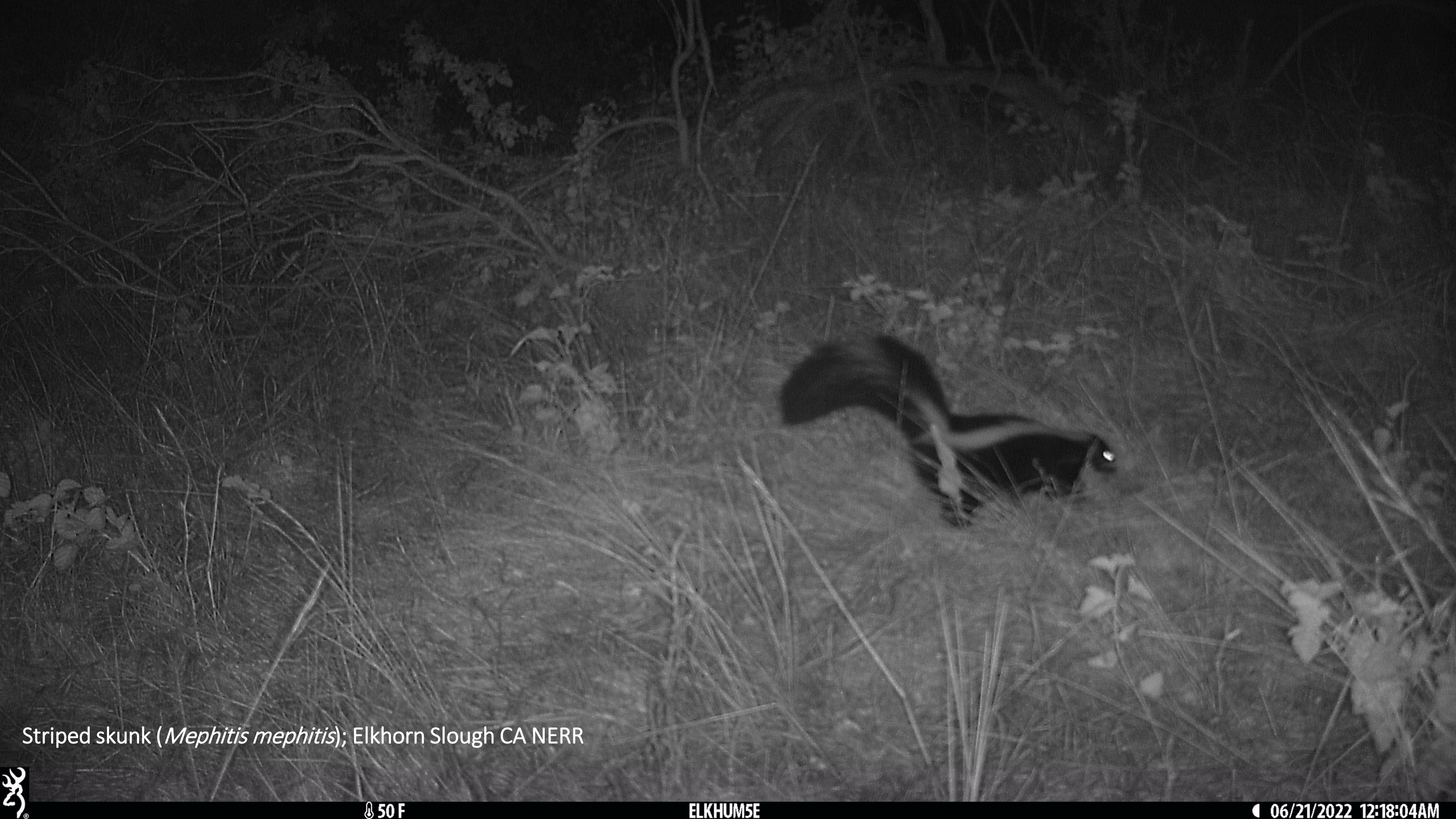

Striped skunk (*Mephitis mephitis*); Elkhorn Slough CA NERR

Raptors in coastal wetlands

Note: these are example images from this study; they are not inclusive of all raptors found in coastal wetlands.

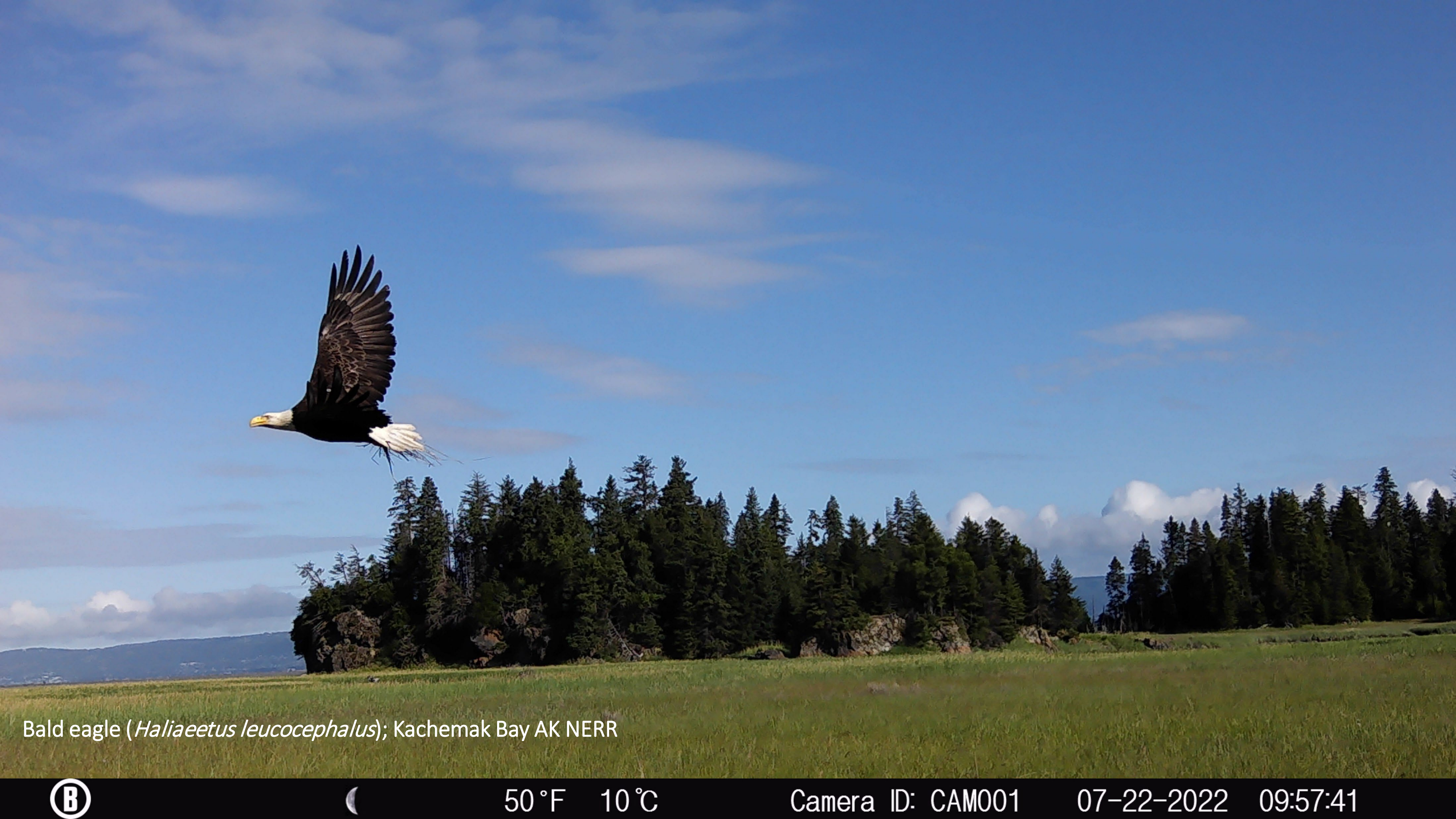

Bald eagle (*Haliaeetus leucocephalus*); Kachemak Bay AK NERR

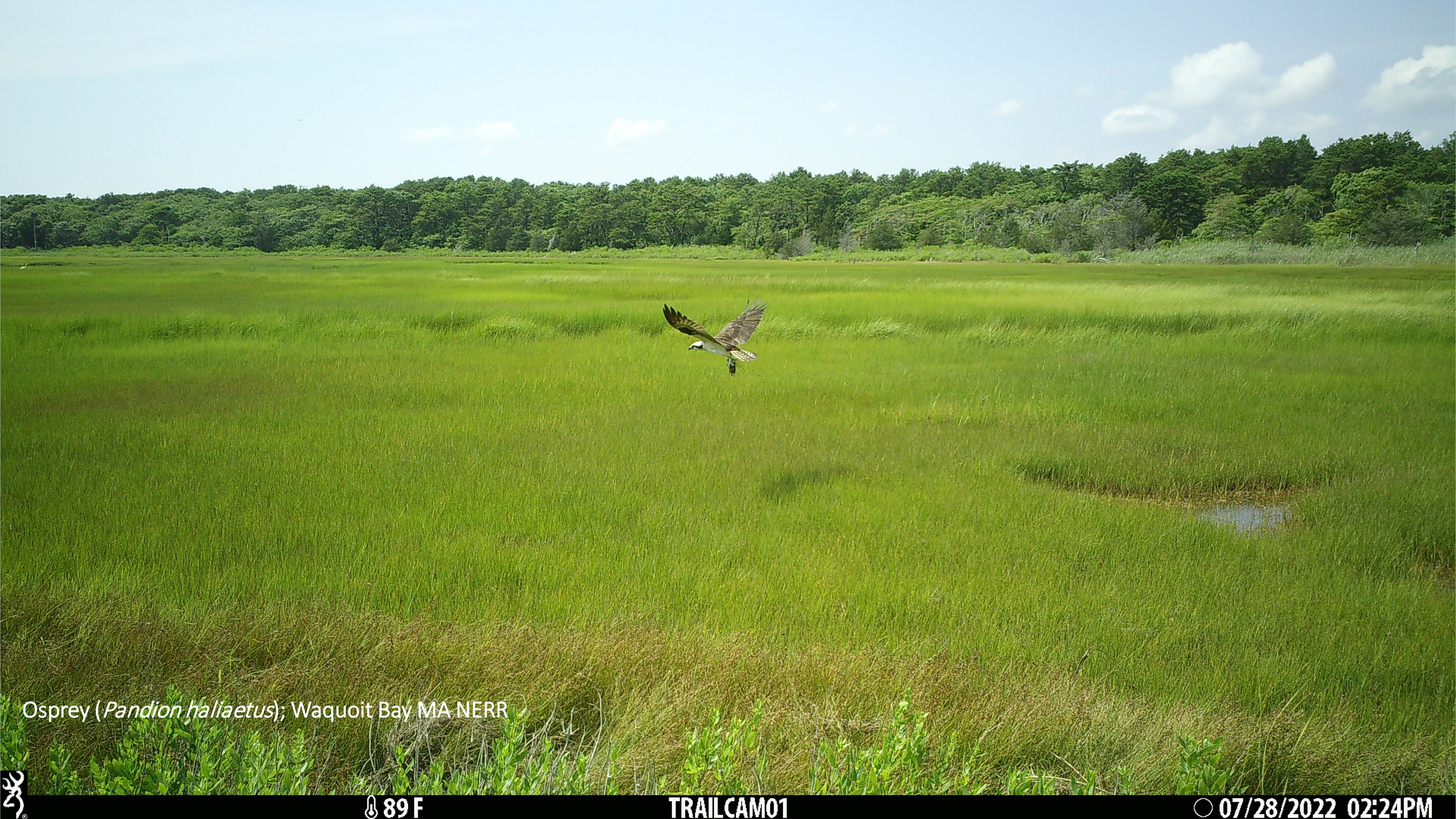

Osprey (*Pandion haliaetus*); Waquoit Bay MA NERR

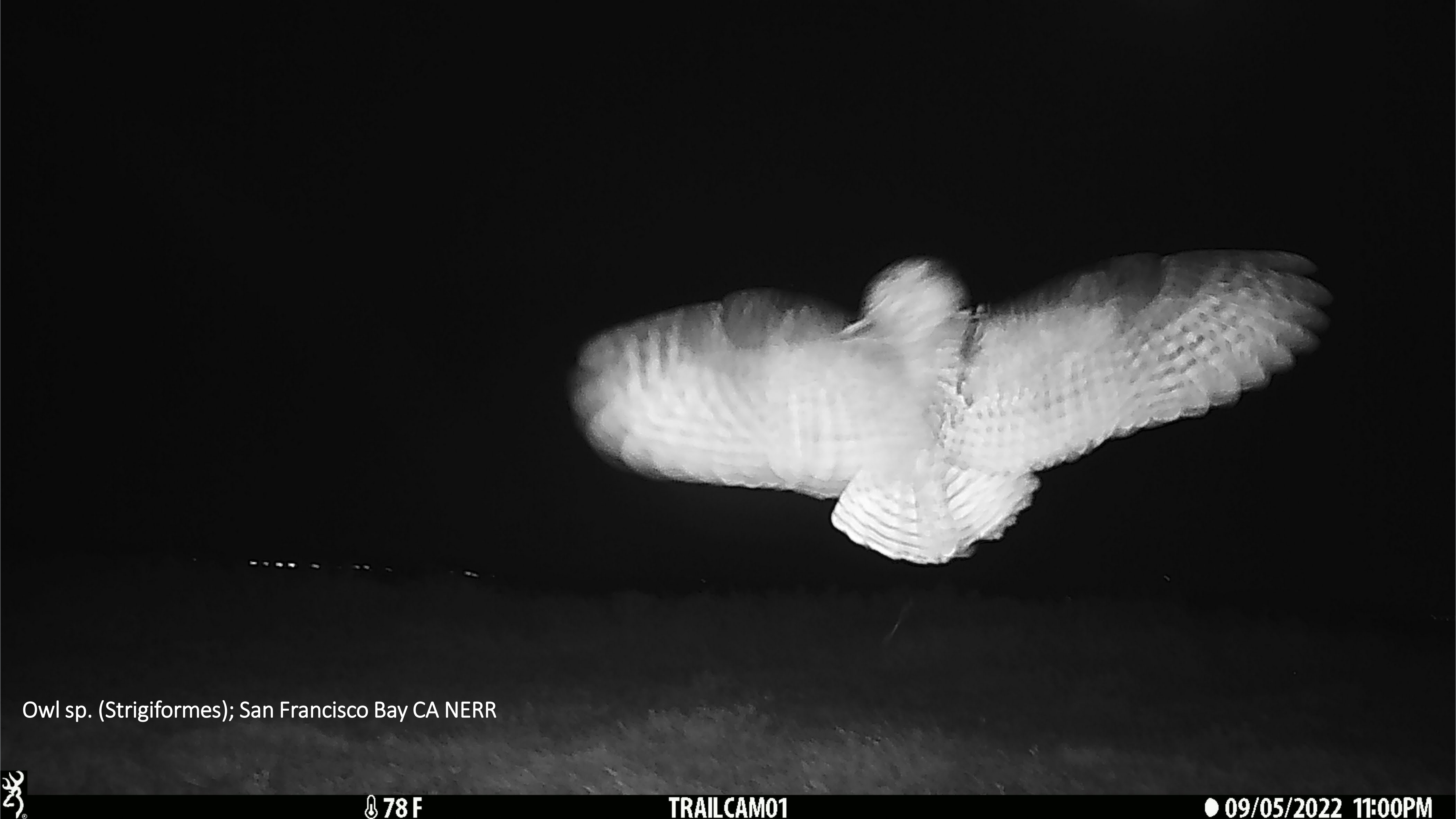

Owl sp. (Strigiformes); San Francisco Bay CA NERR

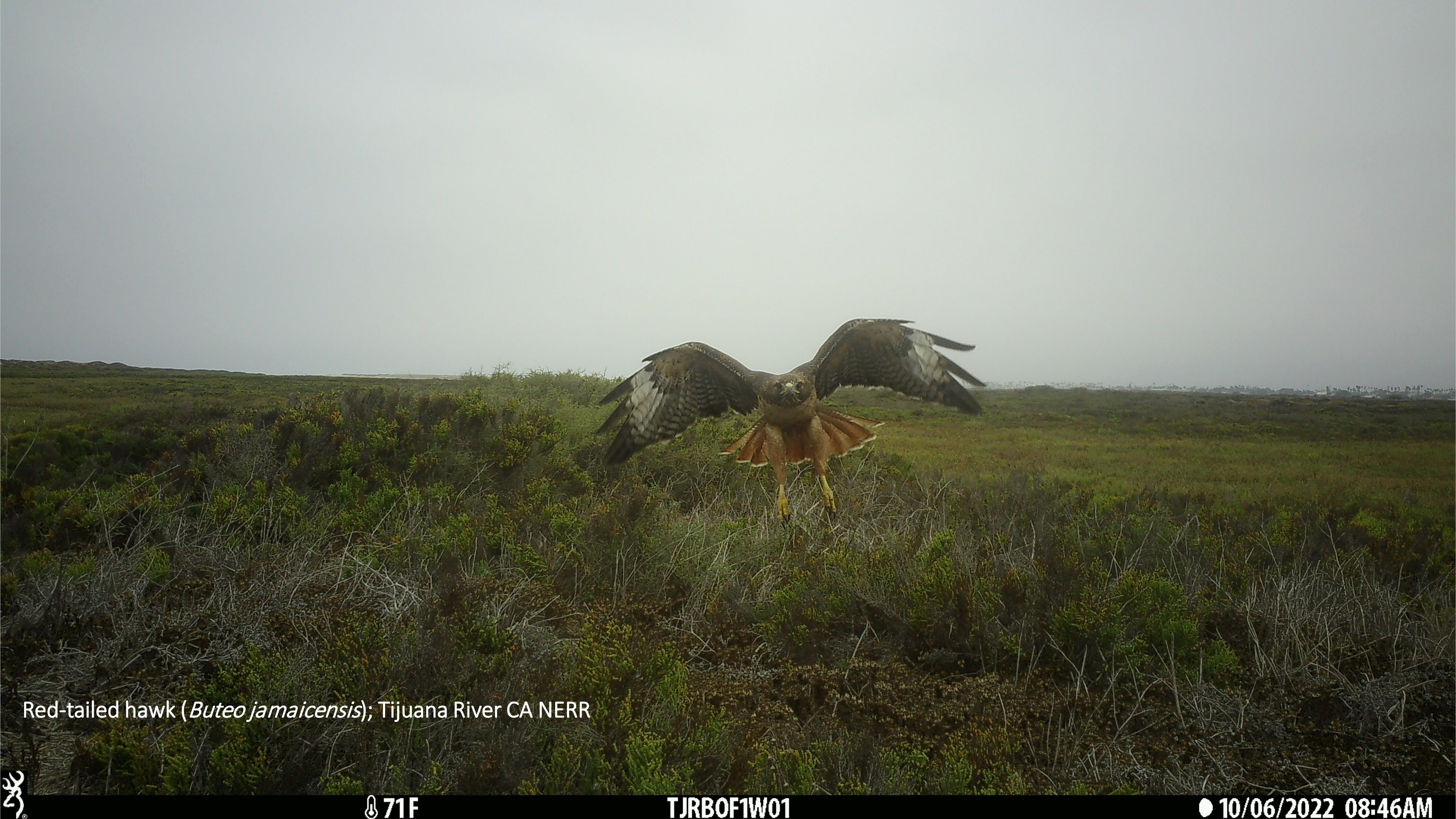

Red-tailed hawk (*Buteo jamaicensis*); Tijuana River CA NERR

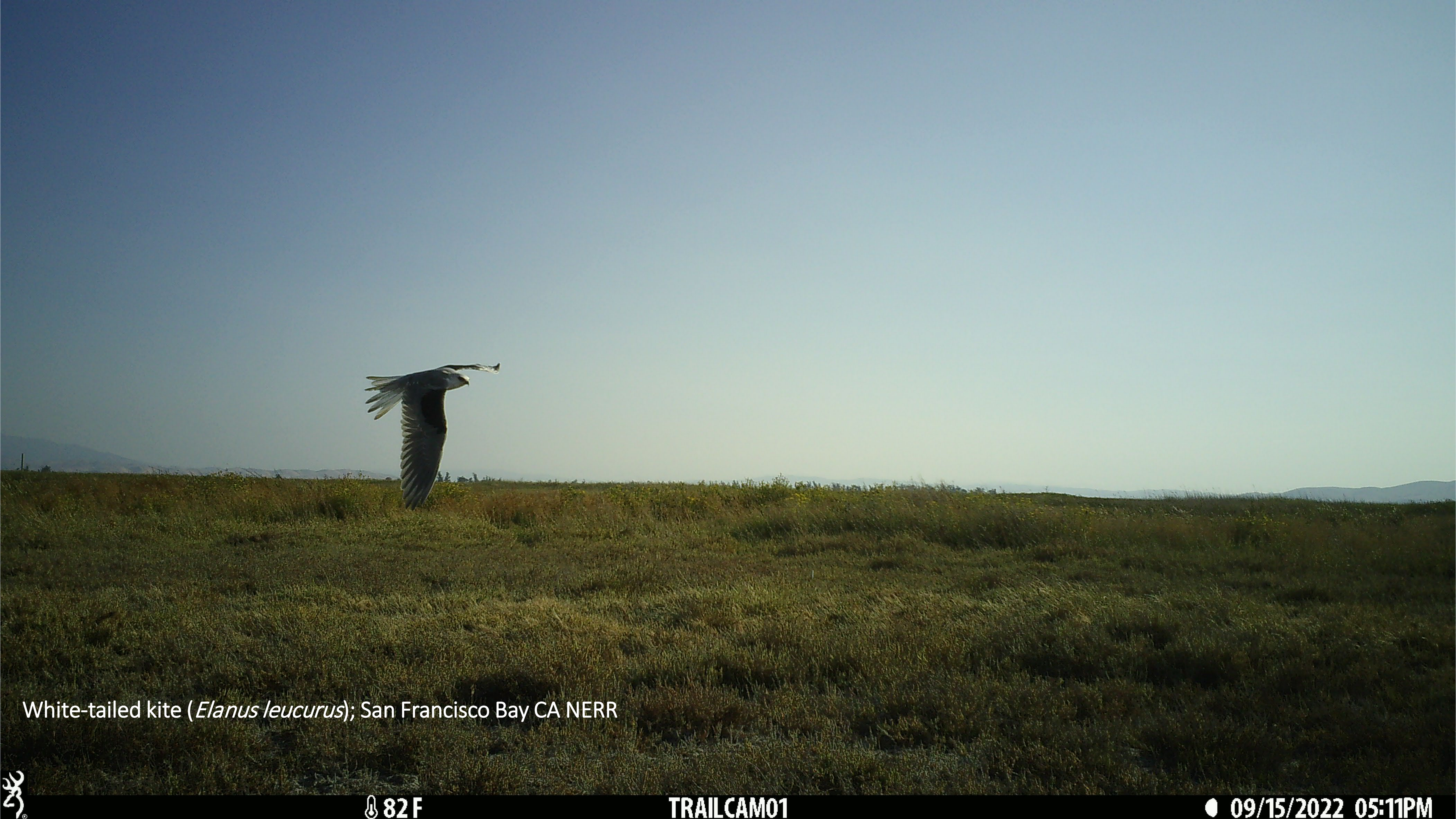

White-tailed kite (*Elanus leucurus*); San Francisco Bay CA NERR

Non-native and feral domestic species in coastal wetlands

Note: these are example images from this study; they are not inclusive of all non-native species found in coastal wetlands.

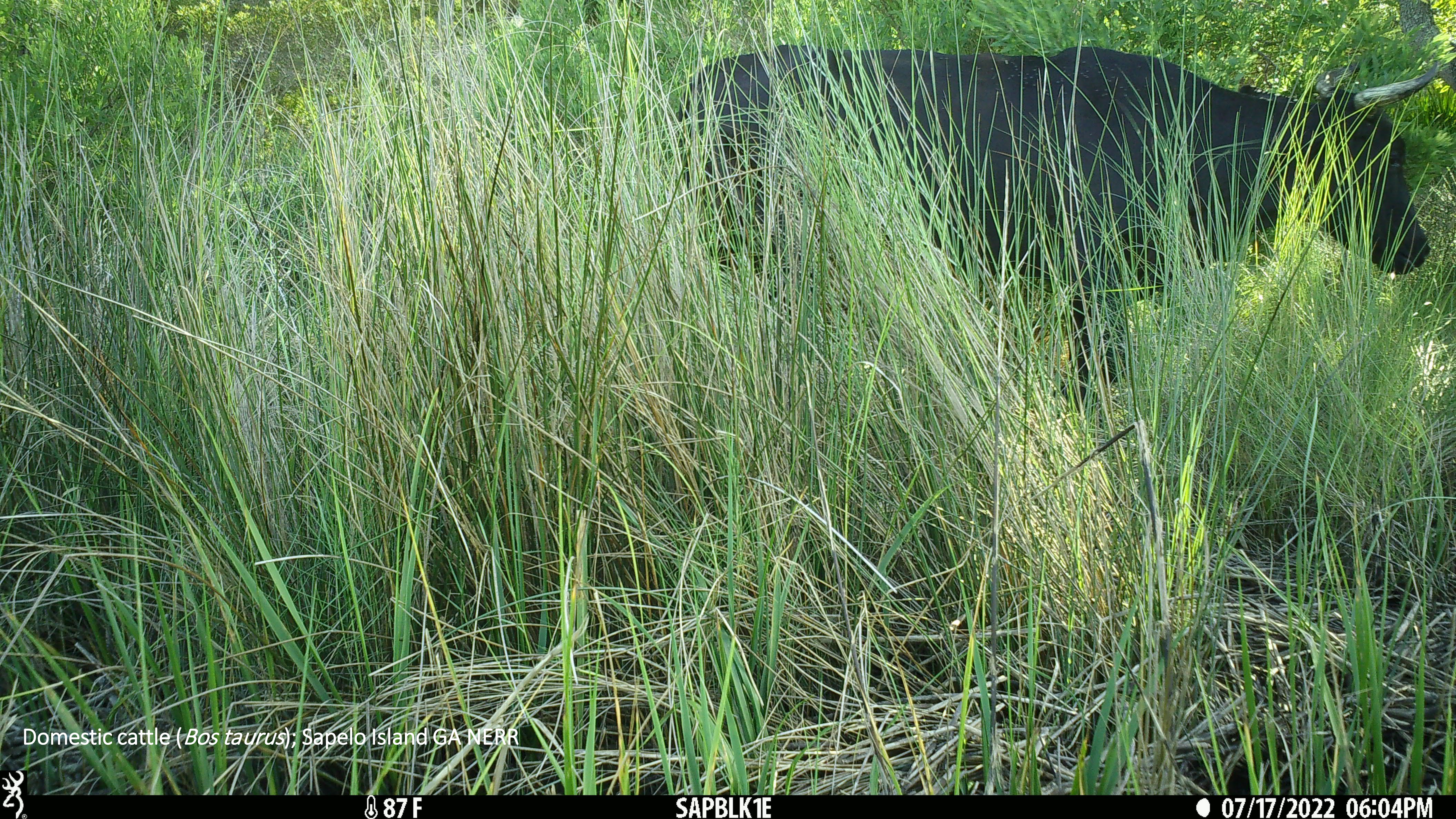

Domestic cattle (*Bos taurus*); Sapelo Island GA NERR

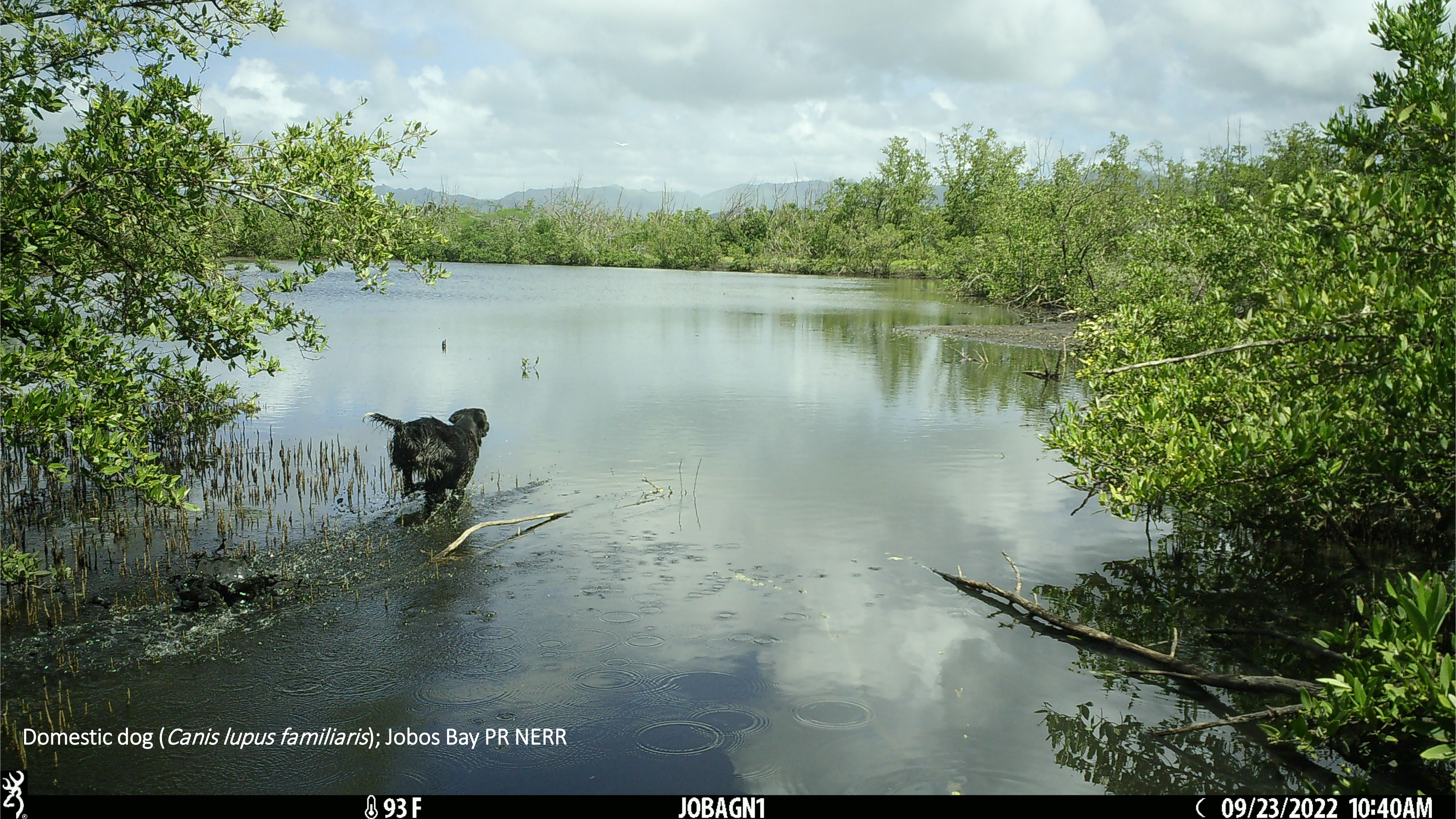
Domestic dog (*Canis lupus familiaris*); Jobos Bay PR NERR
93 F
JOBAGN1
09/23/2022 10:40AM

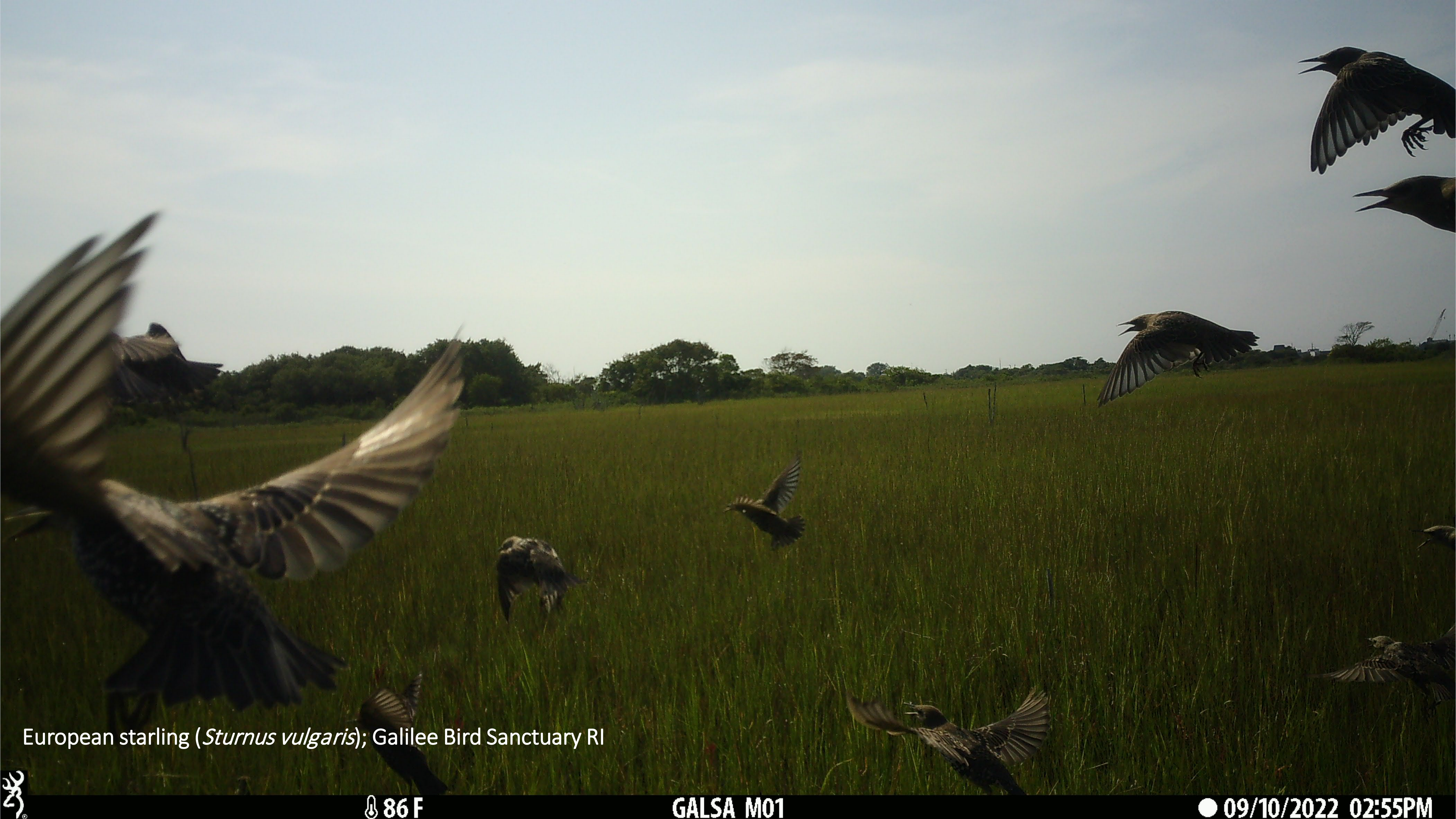

European starling (*Sturnus vulgaris*); Galilee Bird Sanctuary RI

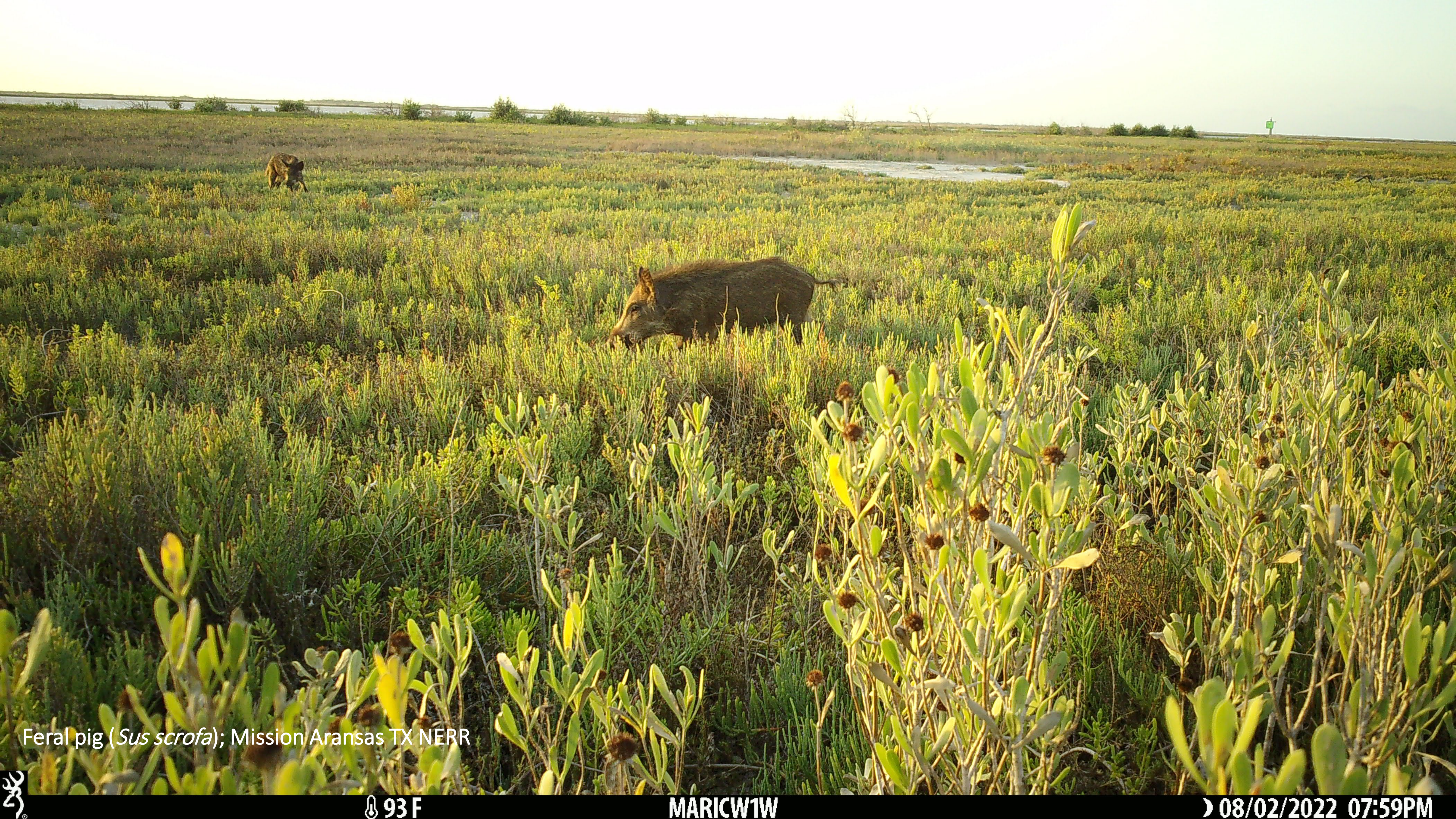

Feral pig (*Sus scrofa*); Mission Aransas TX NERR

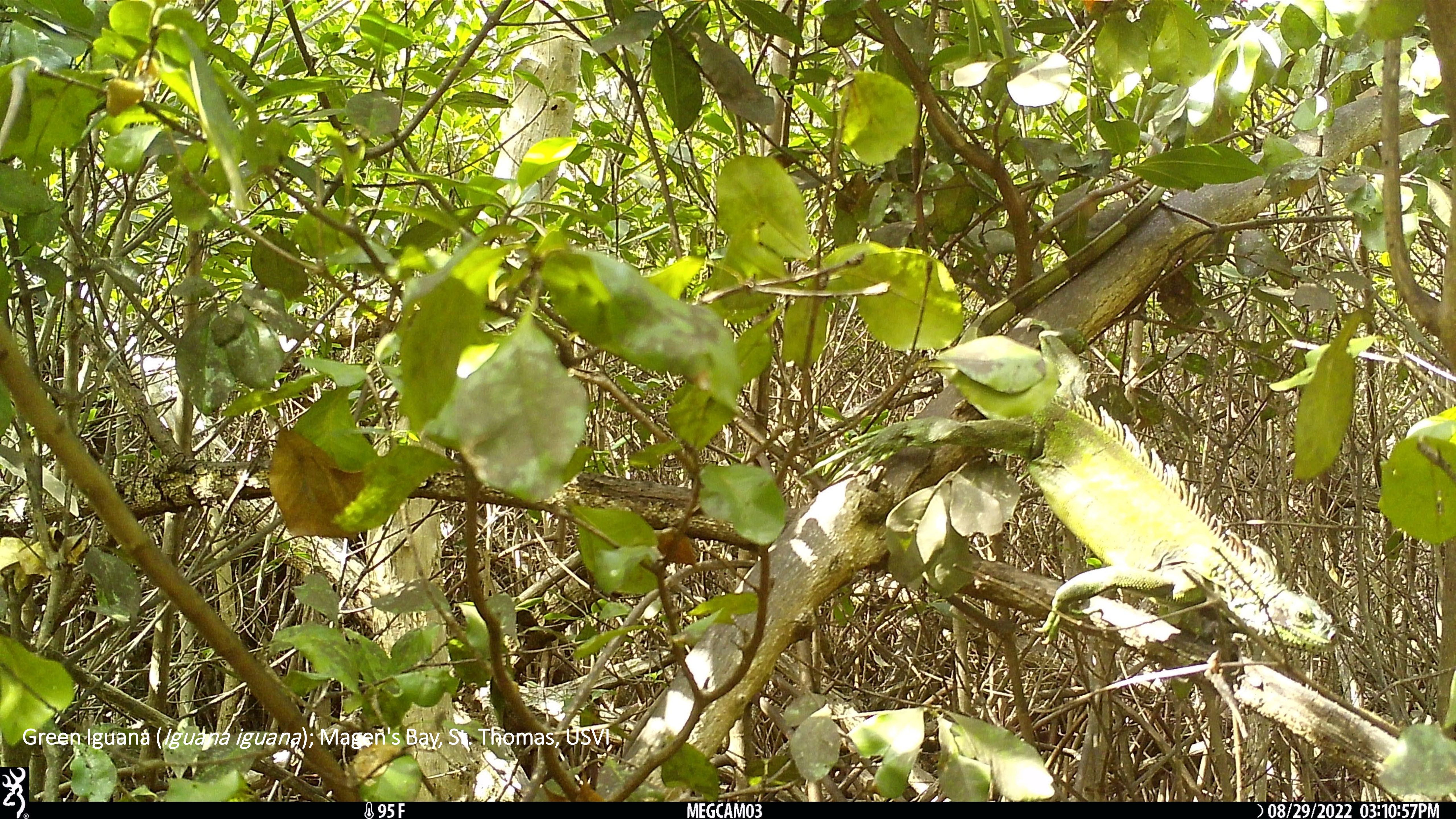
Green Iguana (*Iguana iguana*); Magen's Bay, St. Thomas, USVI
95 F
MEGCAM03
08/29/2022 03:10:57PM

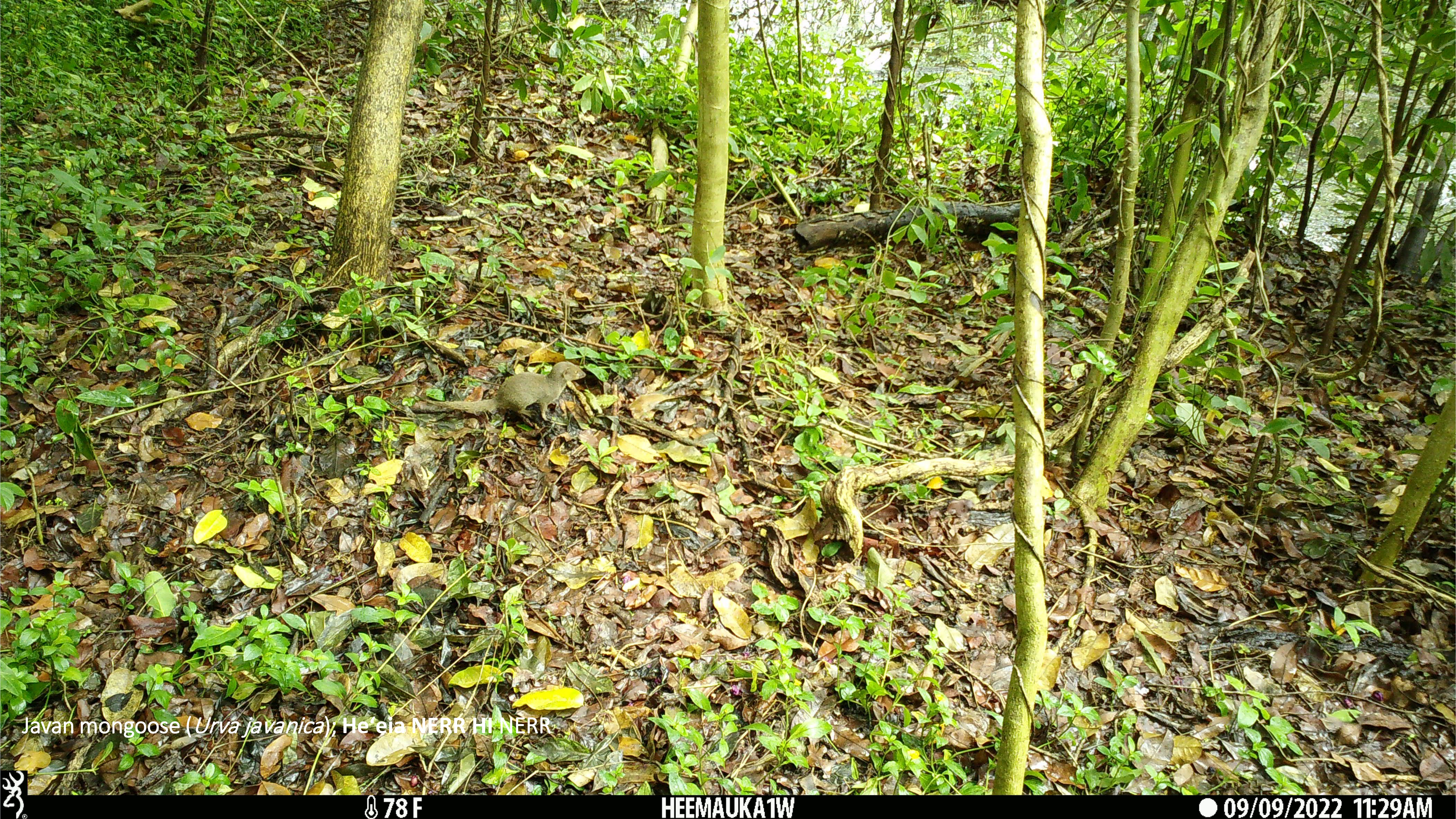
Javan mongoose (*Urva javanica*), He'eia NERR HI NERR
78 F
HEEMAUKA1W
09/09/2022 11:29AM

Nursery function of coastal wetlands for wildlife

Note: these are example images from this study; they are not inclusive of all instances of wildlife using coastal wetlands as a nursery.

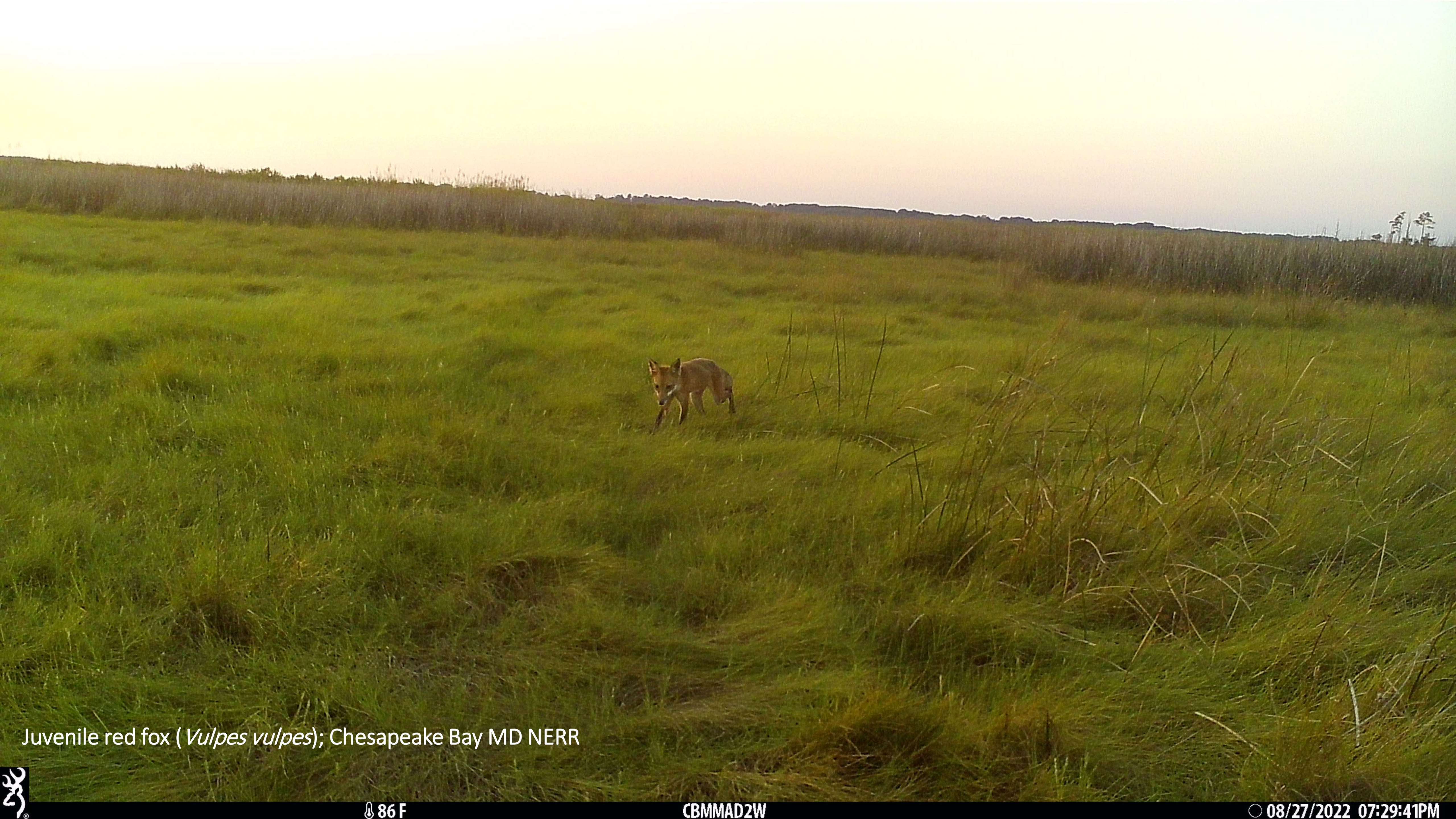

Juvenile red fox (*Vulpes vulpes*); Chesapeake Bay MD NERR

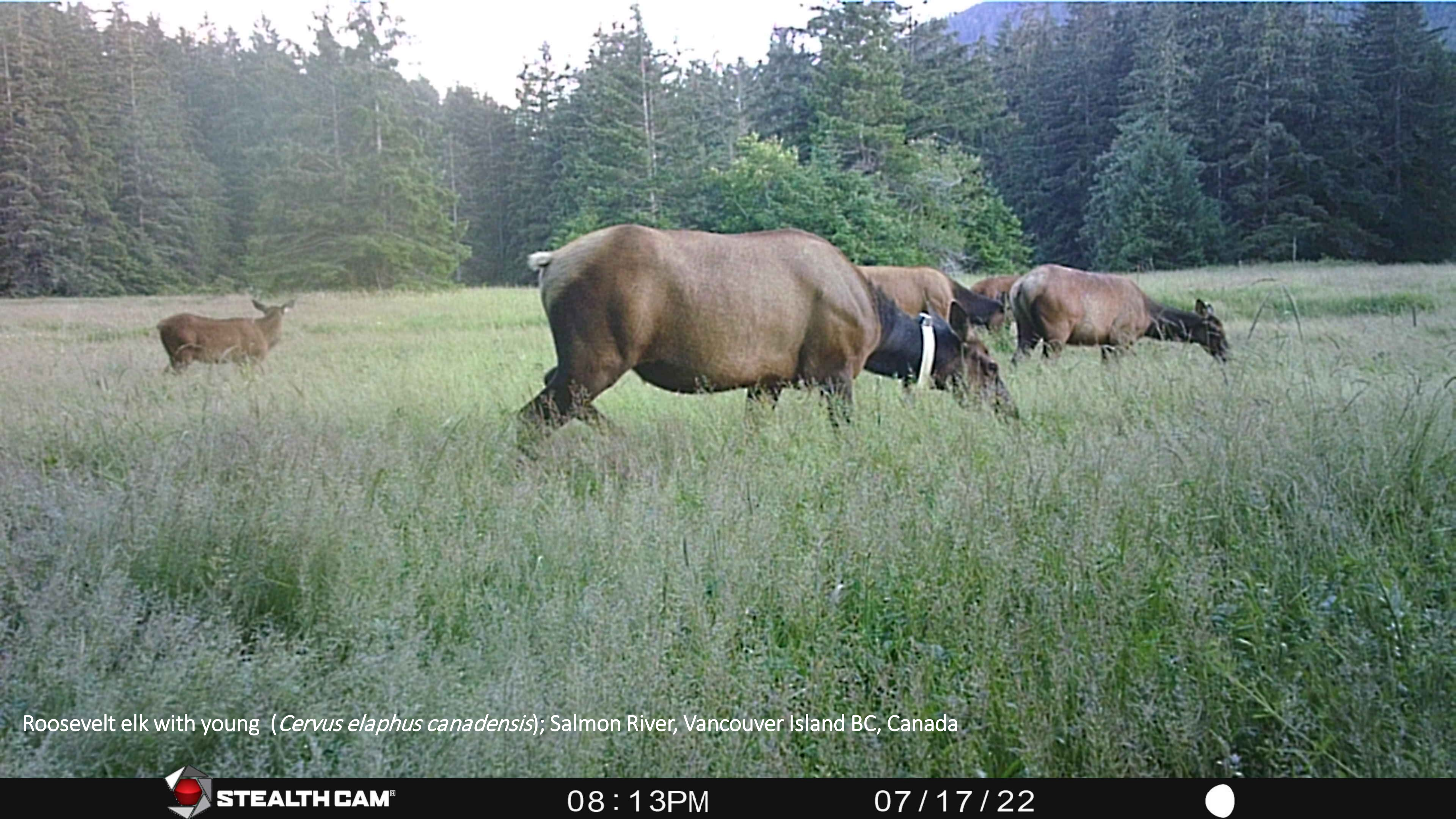

Roosevelt elk with young (*Cervus elaphus canadensis*); Salmon River, Vancouver Island BC, Canada

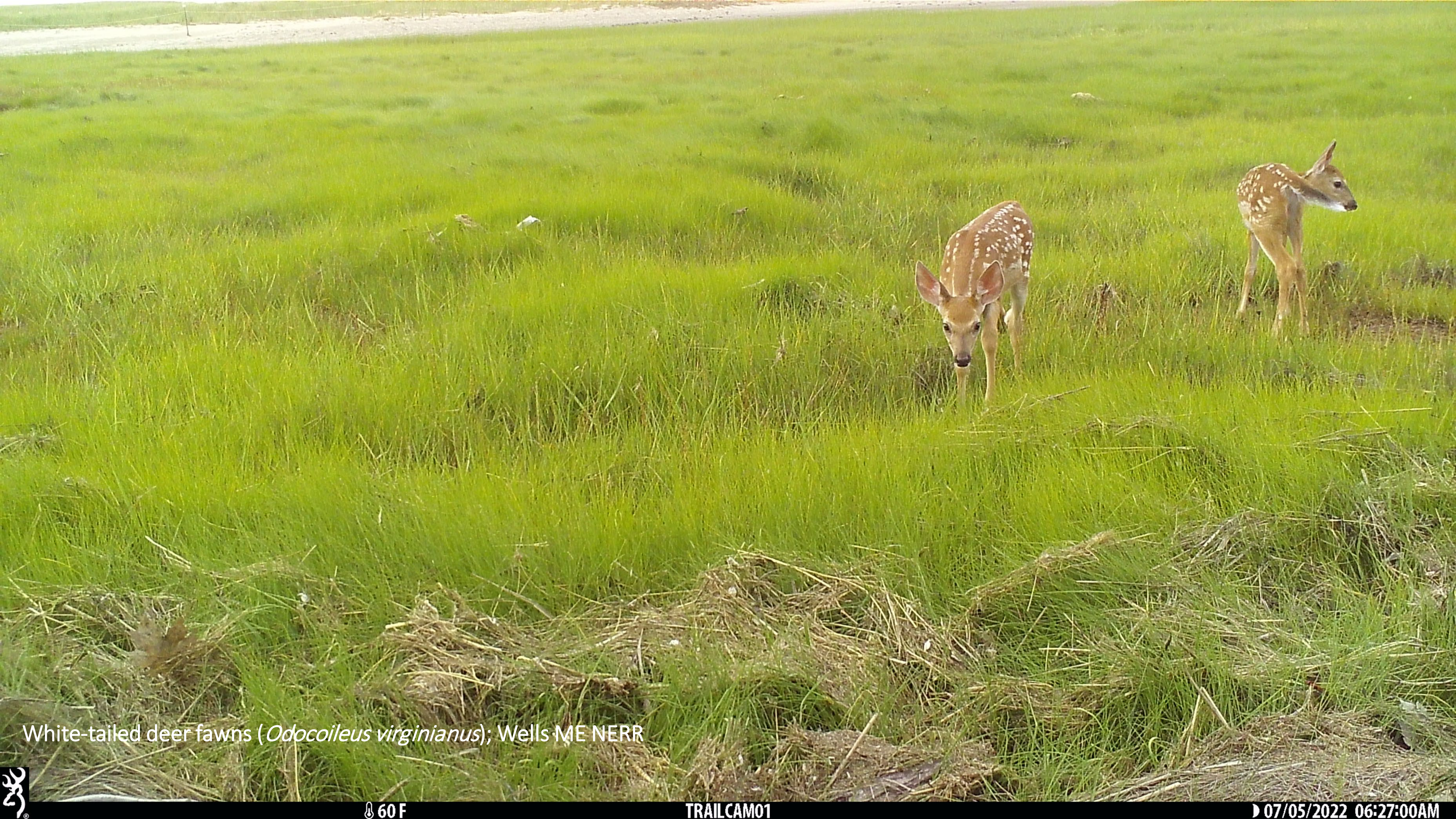

White-tailed deer fawns (*Odocoileus virginianus*); Wells ME NERR

Additional images of wildlife in wetlands across the study sites

Note: these are just a few of many images of wildlife using coastal wetlands documented in this study.

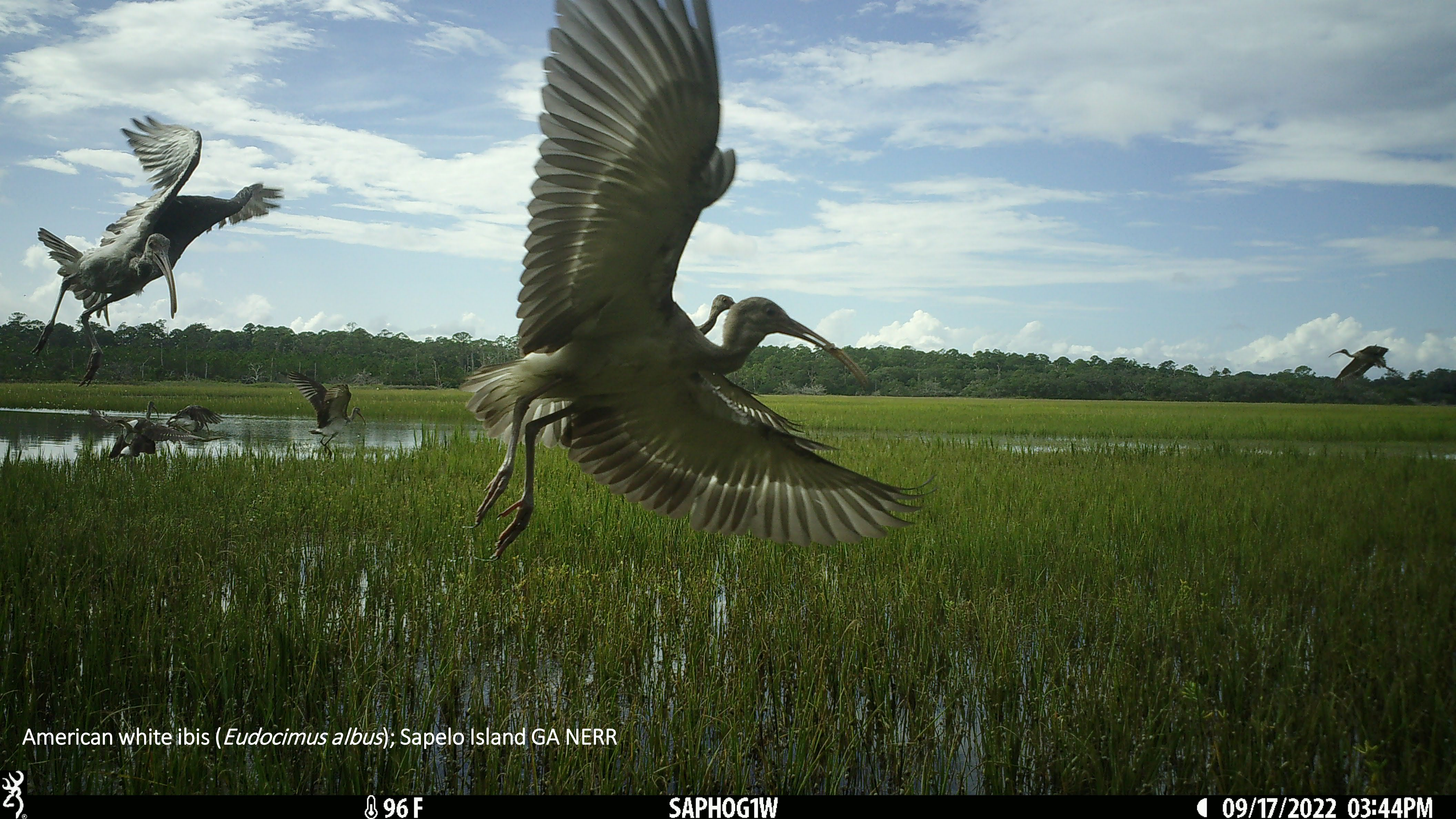

American white ibis (*Eudocimus albus*); Sapelo Island GA NERR

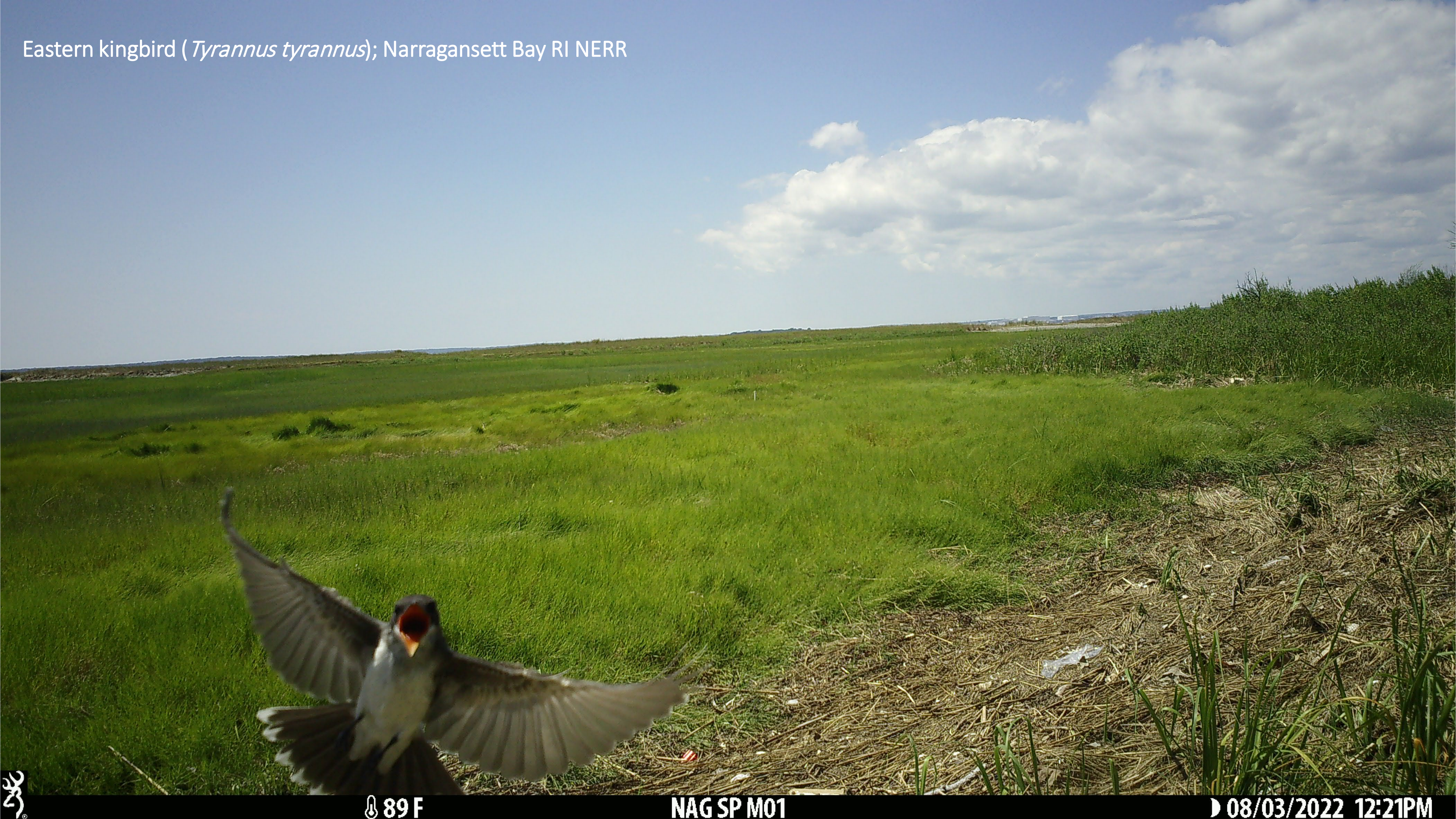

Eastern kingbird (*Tyrannus tyrannus*); Narragansett Bay RI NERR

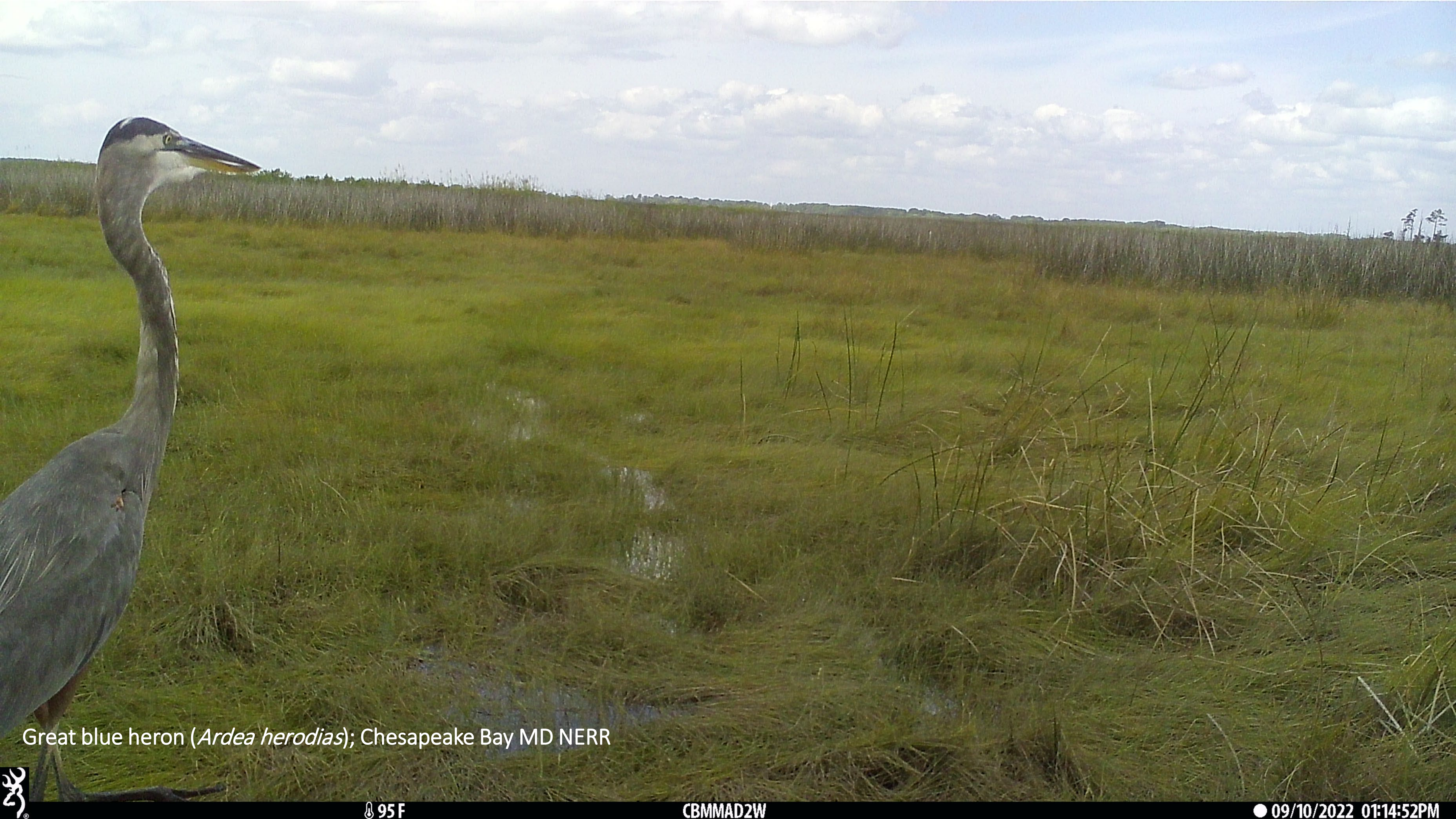

Great blue heron (*Ardea herodias*); Chesapeake Bay MD NERR

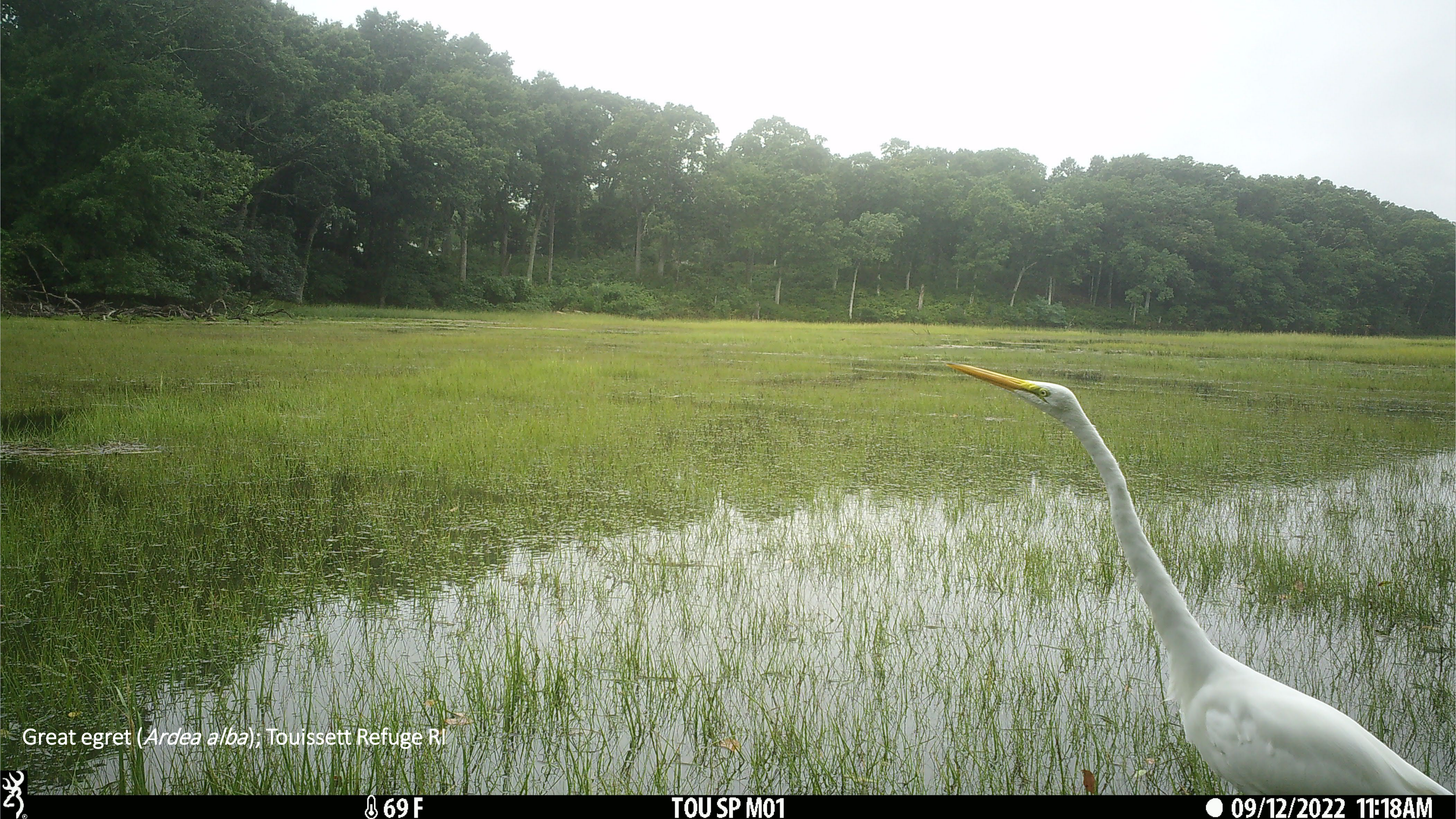

Great egret (*Ardea alba*); Touissett Refuge RI

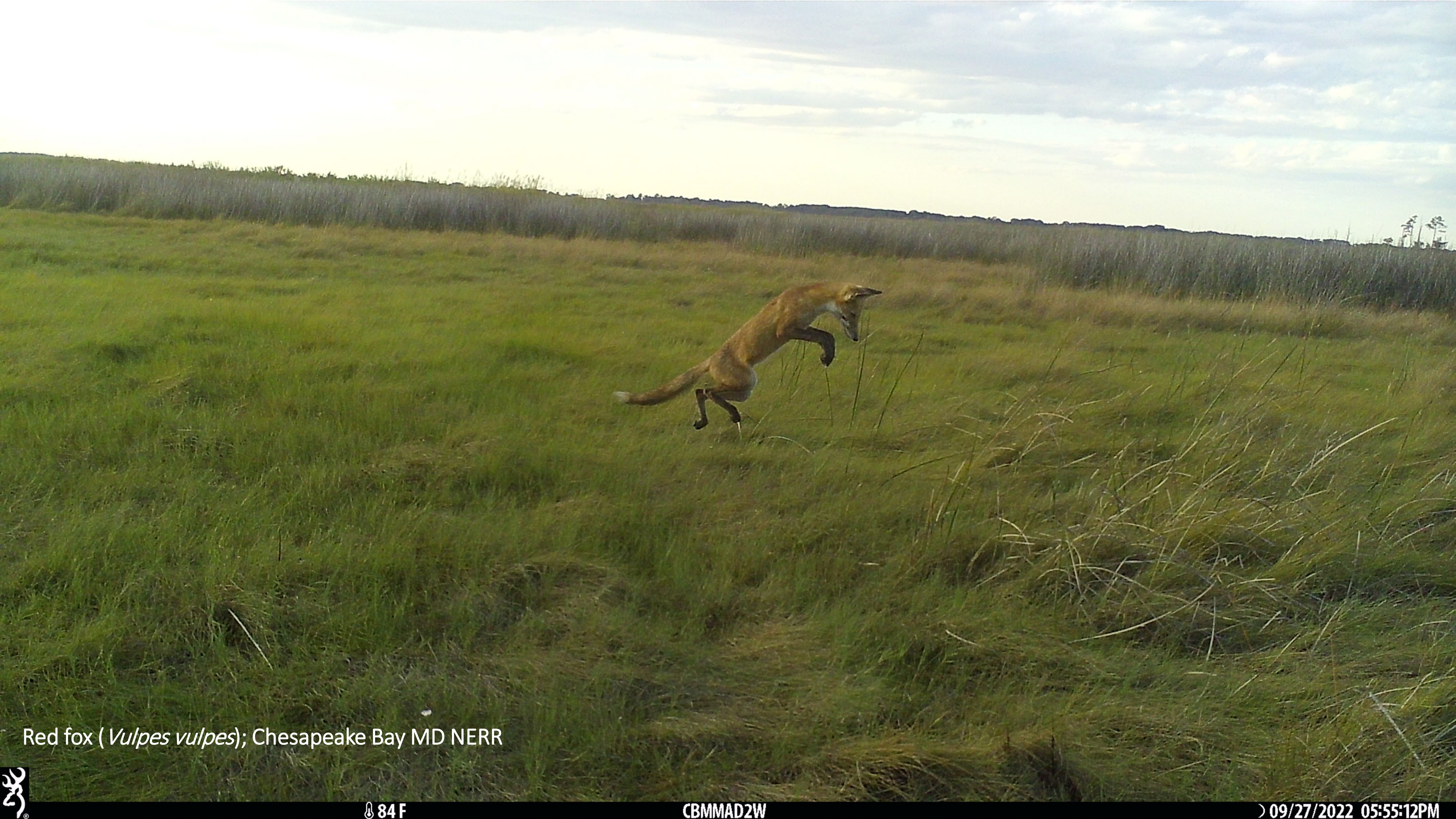

Red fox (*Vulpes vulpes*); Chesapeake Bay MD NERR

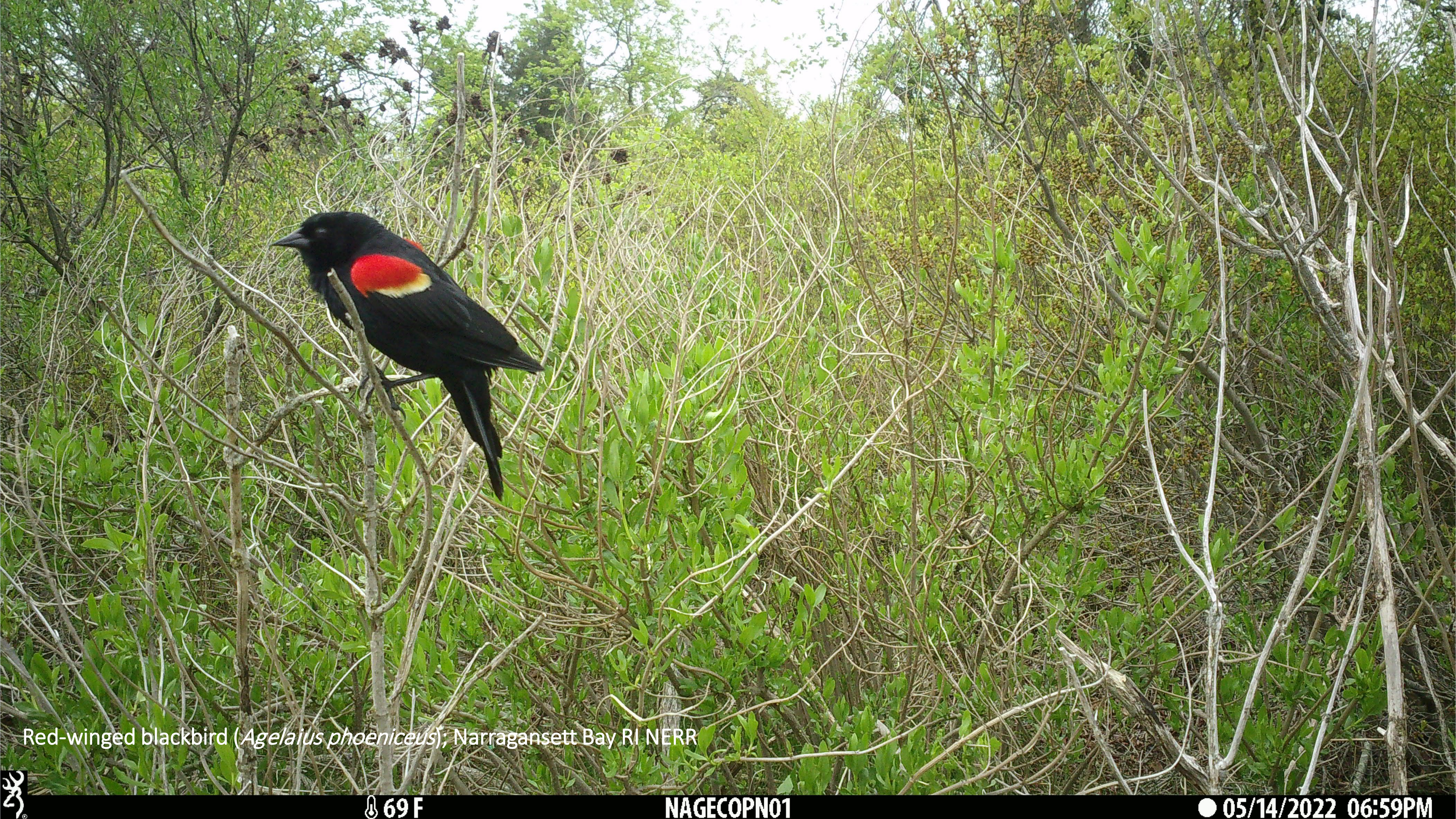

Red-winged blackbird (*Agelaius phoeniceus*); Narragansett Bay RI NERR

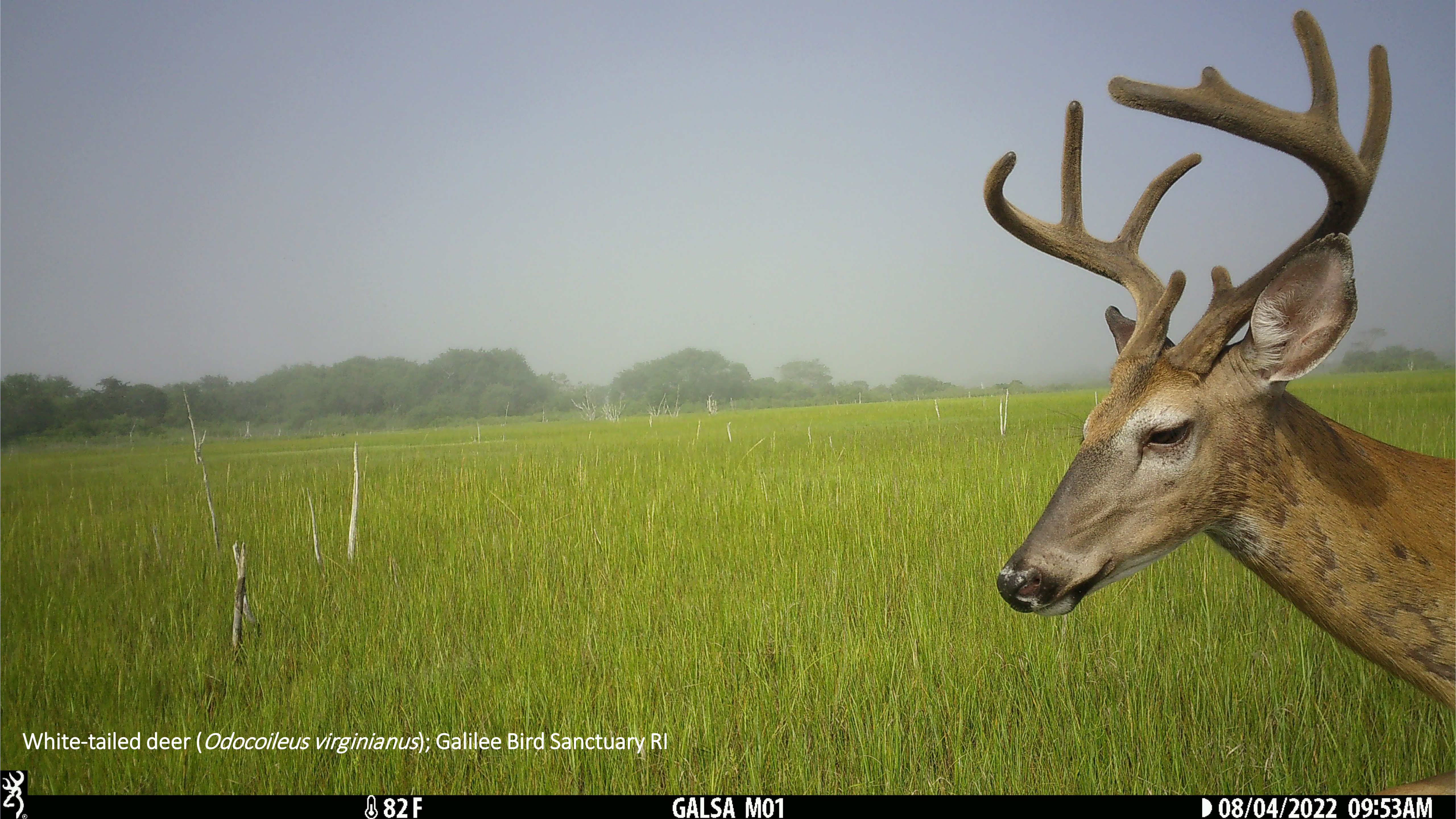

White-tailed deer (*Odocoileus virginianus*); Galilee Bird Sanctuary RI

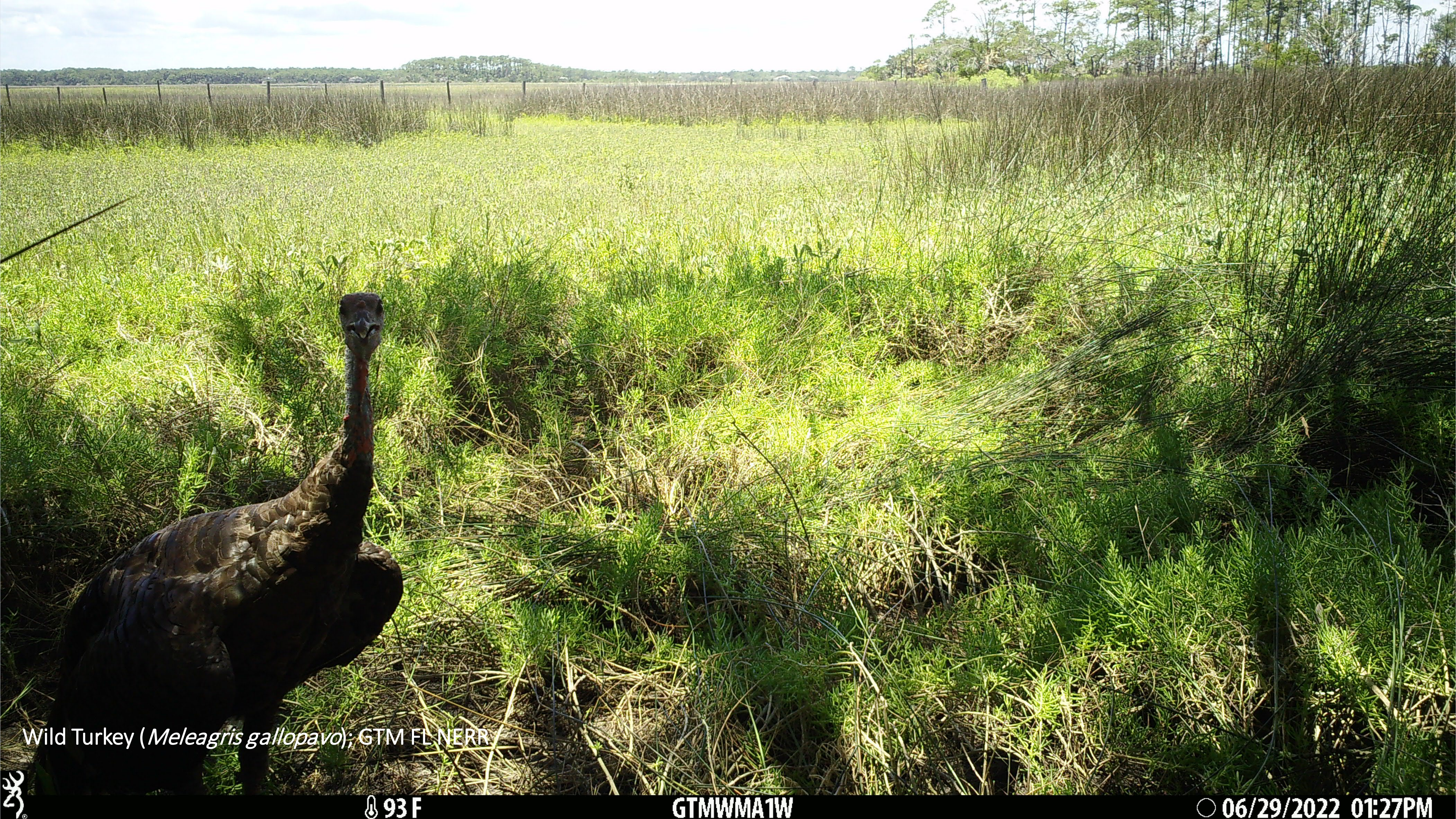

Wild Turkey (*Meleagris gallopavo*); GTM FL NERR

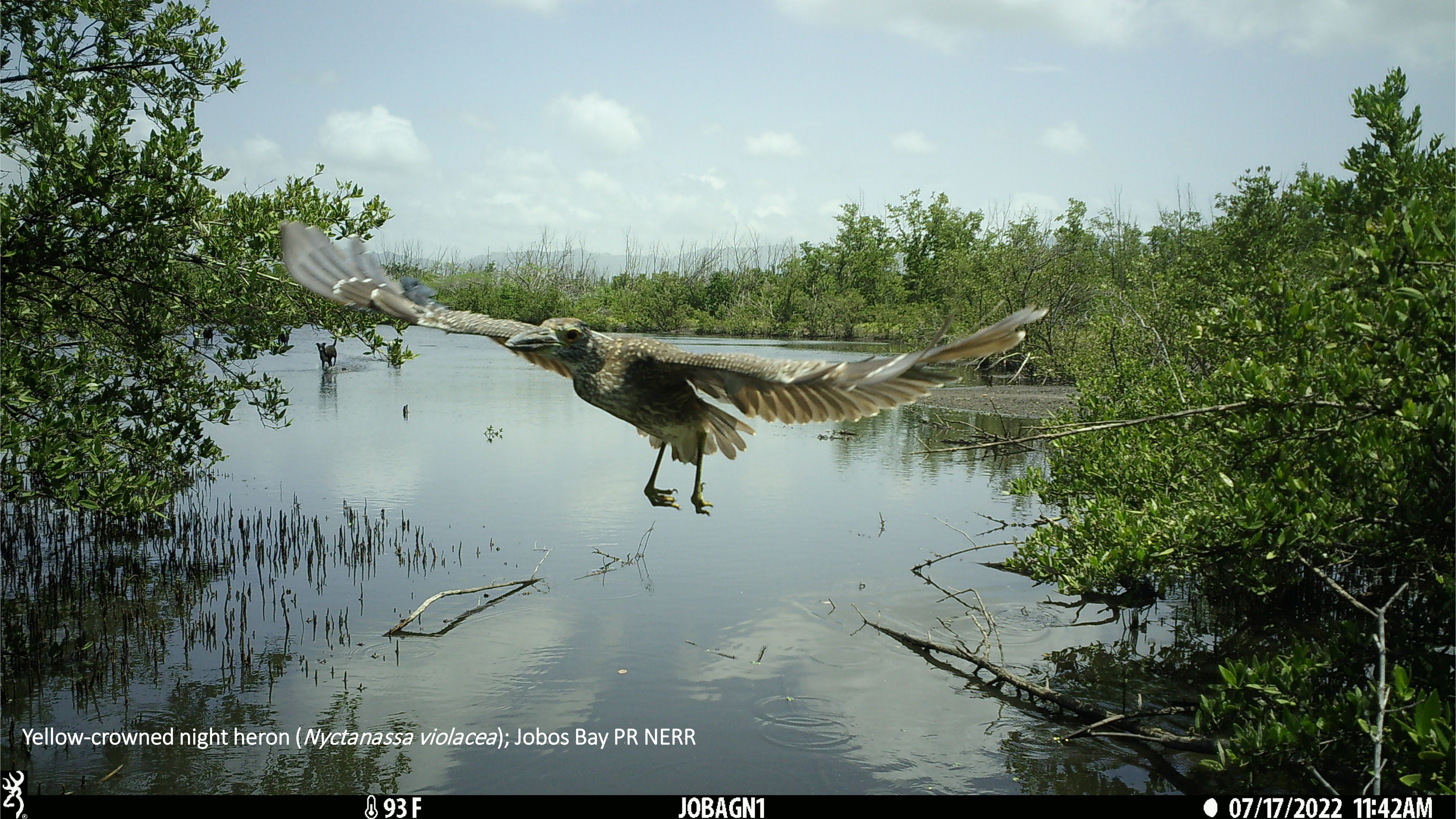

Yellow-crowned night heron (*Nyctanassa violacea*); Jobos Bay PR NERR
